# Discovery of Potent Inhibitors for the Large Neutral Amino Acid Transporter 1 (LAT1) by Structure-Based Methods

**DOI:** 10.3390/ijms20010027

**Published:** 2018-12-21

**Authors:** Natesh Singh, Mariafrancesca Scalise, Michele Galluccio, Marcus Wieder, Thomas Seidel, Thierry Langer, Cesare Indiveri, Gerhard F. Ecker

**Affiliations:** 1 Department of Pharmaceutical Chemistry, University of Vienna, Althanstrasse 14, 1090 Wien, Austria; singh.natesh@gmail.com (N.S.); marcus.wieder@univie.ac.at (M.W.); thomas.seidel@univie.ac.at (T.S.); thierry.langer@univie.ac.at (T.L.); 2 Department DiBEST, Unit of Biochemistry & Molecular Biotechnology, University of Calabria, Via P. Bucci 4C, 87036 Arcavacata di Rende, Italy; mariafrancesca.scalise@unical.it (M.S.); michele.galluccio@unical.it (M.G.); cesare.indiveri@unical.it (C.I.)

**Keywords:** LAT1, amino acid transporter, cancer, virtual screening, inhibitor, proteoliposomes

## Abstract

The large neutral amino acid transporter 1 (LAT1) is a promising anticancer target that is required for the cellular uptake of essential amino acids that serve as building blocks for cancer growth and proliferation. Here, we report a structure-based approach to identify chemically diverse and potent inhibitors of LAT1. First, a homology model of LAT1 that is based on the atomic structures of the prokaryotic homologs was constructed. Molecular docking of nitrogen mustards (NMs) with a wide range of affinity allowed for deriving a common binding mode that could explain the structure−activity relationship pattern in NMs. Subsequently, validated binding hypotheses were subjected to molecular dynamics simulation, which allowed for extracting a set of dynamic pharmacophores. Finally, a library of ~1.1 million molecules was virtually screened against these pharmacophores, followed by docking. Biological testing of the 30 top-ranked hits revealed 13 actives, with the best compound showing an IC_50_ value in the sub-μM range.

## 1. Introduction

The Large neutral Amino acid Transporter 1 (LAT1, or SLC7A5) is a transmembrane protein belonging to the Heteromeric Amino acid Transporter (HAT) family that is responsible for cellular uptake of hydrophobic amino acids (e.g., phenylalanine and tyrosine). LAT1 is substantially expressed at the Blood-Brain Barrier (BBB), where it permits the influx of essential amino acids that are indispensable for brain metabolism. At the BBB, LAT1 also mediates the uptake of thyroid hormones, prescription drugs, (e.g., melphalan, gabapentin, and L-DOPA), and metabolites [1,2,3]. For these reasons, LAT1 is a target of interest for brain drug delivery in the treatment of neurological disorders [4]. Moreover, drugs that show reduced or suboptimal BBB penetrability can be altered to achieve efficient LAT1 mediated transport into the brain, thus empowering their biological effect [5]. Importantly, the anomalous upregulation of LAT1 activity has been demonstrated in several types of cancer, such as breast cancer, pancreatic cancer, prostate cancer, non-small cell lung cancer, esophageal squamous cell carcinoma, colorectal, head, and neck cancer and gliomas [6,7,8,9,10,11,12,13,14,15].

**Figure 1:**
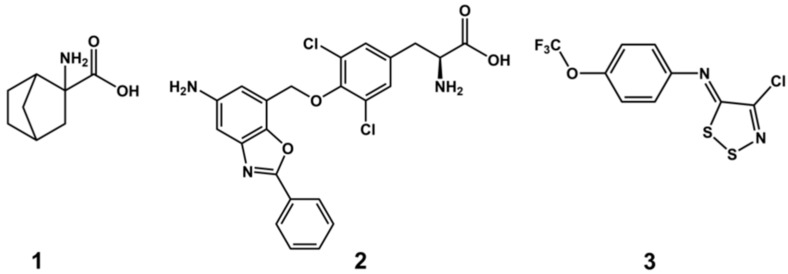
Chemical structures of Large neutral Amino acid Transporter 1 (LAT1) inhibitors 2-Amino-2-norbornanecarboxylic acid (BCH) (**1**), KYT-0353 (**2**), and (Z)-4-chloro-N-(4-(trifluoromethoxy)phenyl)-5H-1,2,3-dithiazol-5-imine (**3**).

Since LAT1 expression is crucial for the sustenance, growth, and proliferation of cancer [16], its inhibition could offer a therapeutic approach to suppress malignant tumors. LAT1 inhibitors act by thwarting the supply of amino acids to cancer cells, thus impeding the protein synthesis and cellular proliferation. In addition, protein synthesis and cell growth have been shown to be regulated by the amino acid leucine through stimulation of the mechanistic/mammalian target of the rapamycin (mTOR) pathway. Hence, LAT1 inhibition has been considered as a propitious strategy to suppress mTOR signalling, and subsequently cancer growth [16]. Despite the profound pharmacological interest, up to now only a few potent LAT1 inhibitors are available, necessitating the identification of novel compounds in this area. Prominent examples include 2-Amino-2-norbornanecarboxylic acid (BCH) (**1**) (Figure 1), which is deemed as a standard inhibitor of all large amino acid transporters (LAT1−4, or System L) and it is described to induce the suppression of cancer growth and apoptosis [6,17,18]. However, BCH is a low-affinity and non-selective substrate of LAT1, and a very high concentration (>10 mM) is required to attain antiproliferative effects [19,20,21]. The novel tyrosine analog, KYT-0353 (JPH-203) (**2**) is a potent and selective inhibitor of LAT1, with an IC_50_ of 0.14 μM in S2 cells and 0.06 μM in human colon cancer (HT-29) cells. It also suppressed the growth of cancer cells and xenograft tumors [22]. Additionally, a recent publication reported 1,2,3-dithiazole based irreversible covalent inhibitors (**3**) that showed potent inhibition of human LAT1 reconstituted in proteoliposomes [23]. 

With the determination of the X-ray structures of the prokaryotic homologs [24,25,26], homology models of LAT1 have been constructed to explain the substrate binding site [5,27], protein-ligand interaction [5,28], and structure-function relationship [27,29]. Moreover, the use of a homology model combined with docking-based *in silico* screening of a large chemical library has led to the discovery of novel ligands of LAT1 [5]. Also, past efforts utilizing ligand-based methods, such as three-dimensional quantitative structure-activity relationship (3D QSAR) [30,31] and pharmacophore modeling [32], have been successful in designing high-affinity ligands of LAT1.

Nitrogen mustard (NM) derivatives of phenylalanine show high affinity for LAT1 [33,34], however, their molecular basis of inhibition has remained elusive for decades due to the lack of any experimental evidence for the putative binding hypothesis. In the present study, we combined homology modeling, molecular docking, molecular dynamics (MD), and virtual screening with experimental testing to identify novel inhibitors of LAT1. A homology model of human LAT1 was employed for docking studies of the NMs to derive a common binding mode (CBM). Docking poses were subjected to MD simulations to investigate the stability and interaction patterns of the ligands with the protein. Subsequently, a combined dynamic pharmacophore- and docking-based virtual screening of commercially available “lead-like” libraries was performed to predict previously undiscovered LAT1 ligands. From among the top-ranking 0.01% compounds of the database, 30 molecules were experimentally tested for LAT1 inhibition in proteoliposomes to determine their biological efficacy.

## 2. Results

### 2.1. Homology Modeling of LAT1

**Figure 2:**
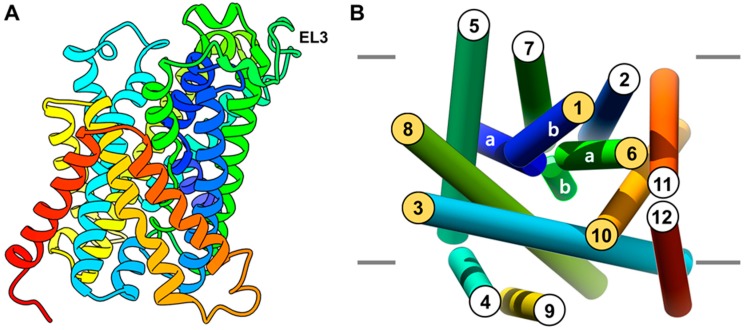
(**A**) Front view of the homology model of LAT1; (**B**)Perpendicular periplasmic view of the model showing the structural organization of the transmembrane segments (TMs).

The homology model of LAT1 was built on the outward-occluded conformation of the arginine/agmatine antiporter AdiC from *Escherichia coli* (PDB ID: 3L1L) [24] and on the inward-open, occluded structure of amino acid/polyamine/organocation (APC) transporter ApcT (PDB ID: 3GIA) from *Methanocaldococcus jannaschii* [26] (see Methods). The constructed models were assessed using MODELLER’s internal scoring function, called Discrete Optimized Protein Energy (DOPE) [35]. The top-ranked model was based on AdiC (Figure 2A) and showed a Z-DOPE score of −0.39, implying that 60% of its Cα atoms are within 3.5Å of their accurate positions, thus indicating a reliable model [36]. Furthermore, the quality of this model was found satisfactory using PROCHECK [37] (Table S2, Figures S3 and S4) and QMEAN [38] (Figure S5). The predicted structure of LAT1 consists of 12 transmembrane segments (TMs) that are comparable in architecture to other prokaryotic members of the APC family that embrace a “LeuT-like fold” [5,39]. The TM1 and TM6 α-helices have a discontinuous structure and they are disentangled at the center (1a-1b; 6a-6b) to harbor the ligand binding site (Figure 2B). TM1a and 1b are linked by the residues I64-S66, and TM6a and 6b by Y254-N258. The most extended loop in the model is EL3, which is 26 residues long from V217-N242 connecting TM5 and TM6 (Figure 2A). Modeling loops of this length are comparatively imprecise, and hence, the modeled loop structure must be regarded as uncertain. As the loop is ~26 Å from the active site, it may not significantly impact the results of subsequent docking studies.

The presence of a well-defined binding pocket in LAT1 was probed through SiteMap [40,41]. The substrate binding site is surrounded by TM1, TM3, TM6, TM8, and TM10 (Figure 2B). The TMs encircling the binding site are amphiphilic helices, comprising both hydrophobic and hydrophilic residues (Figure S6). The predicted binding site residues T62, I63, I64, S66, G67, F252, A253, and G255 were consistent with previously reported studies [5,27]. The Sitescore of the pocket is 1.18, where a score greater > 1 suggests a site of particular promise. The pocket is predicted to be druggable with a Dscore of 1.07 indicating reasonable size, enclosure, and hydrophobicity with modest hydrophilicity. The total surface area of the pocket is ~538Å^2^, of which hydrophobic and hydrophilic regions are ~168Å^2^, and ~285Å^2^, respectively. In contrast to LAT1, the surface area of the AdiC pocket is ~324.58Å^2^, of which hydrophobic and hydrophilic regions are ~75.1Å^2^ and ~216.3Å^2^, respectively. The presence of a large non-polar area in LAT1 may explain the preferential transport of neutral and hydrophobic amino acids with a decent affinity (K_m_ = 15−50 µM) [42], while AdiC primarily exchanges charged arginine and agmatine [25,43]. 

The multiple sequence alignment indicated that that F394 of LAT1 is the putative upper front door residue that is equivalent to F350 of AdiC [44,45]. S66 and F252 of LAT1 are the proximal gate residues that correspond to S26 and W202 of AdiC. The middle layer of LAT1 is S342, corresponding to W293 of AdiC. E136, N258, and A409 are the distal gating residues of LAT1, corresponding to Y93, E208, and Y365 of AdiC [24]. Similar to AdiC, where distal residues are involved in the hydrogen bond network [24], N258 is interacting with the carboxyl of E136 *via* a hydrogen bond in the predicted model of LAT1 [45] (Figure S7) possibly indicating a conserved transport mechanism between LAT1 and AdiC. Additionally, molecular dynamics (MD) simulations as outline below showed that the hydrogen bond between N258 and E136 was consistent (∼92% occupancy), indicating a closed distal gate throughout the simulation (Figure S8).

**Figure 3:**
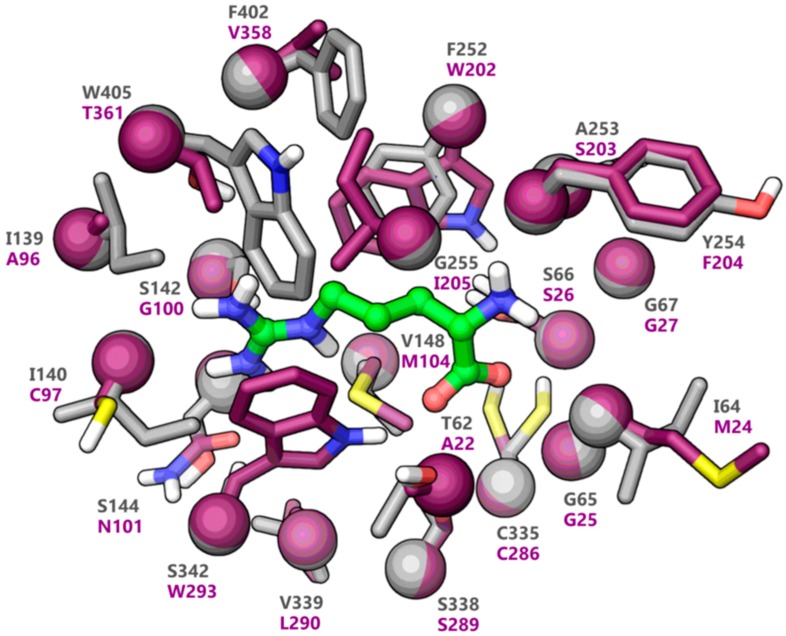
The binding site of LAT1 (grey) superposed to the binding site of AdiC (maroon). The Cα atoms and the side chains are shown in space-filling and stick style, respectively. The arginine (green) bound to AdiC is depicted in stick and ball. Cα root mean square deviation (rmsd): 0.62Å.

**Figure 4:**
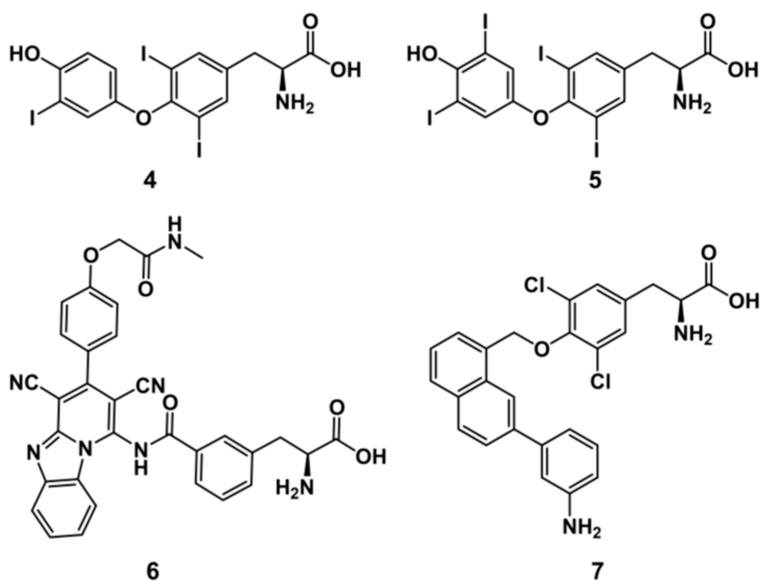
Chemical structures of LAT1 inhibitors. 3,3′,5-triiodothyronine (L-T_3_, **4**), tetraiodothyronine (L-T_4_, **5**), KMH-233 (**6**), and SKN103 (**7**).

The active site alignment of LAT1 and AdiC further revealed that LAT1 has numerous hydrophobic residues, such as aliphatic I139, I140, V148, and aromatic F252, F402, and W405, which seems to foster substrate binding through van der Waals forces, and hydrophobic interactions (e.g., π−π and alkyl) (Figure 3). A few of these aromatic residues are substituted by non-aromatic residues in AdiC. For example, W405 and F402 of LAT1 correspond to T361 and V358 of AdiC. The hydrophobic I140 and A253 of LAT1 correspond to polar C97 and S203 of AdiC. The large residues, like N101, M104, W202, I205, and W293 in AdiC are exchanged by smaller S144, V148, F252, G255, and S342 in LAT1 [5], generating a massive volume in LAT1 (~198Å^3^) as compared to the AdiC (~95Å^3^) (Figure 3). This might explain why LAT1 is proficient in accommodating bulky ligands, such as **2** [22] (Figure 1), **4** [46], **5** [46,47], **6** [31], and **7** [48] (Figure 4).

### 2.2. Docking and Binding Mode Hypothesis

**Figure 5:**
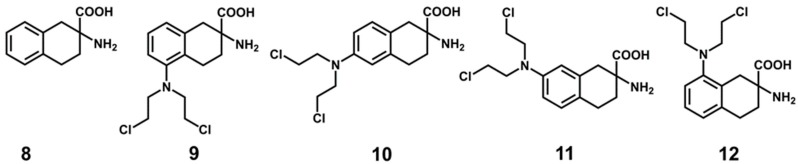
Chemical structures of the ligands used for docking. DL-ANA (2-Amino-1,2,3,4-tetrahydro-2-naphthoic acid, **8**), DL-2-NAM-5 (2-amino-5-bis[(2-chloroethyl)amino]-1,2,3,4-tetrahydro-2-naphthoic acid, **9**), DL-2-NAM-6 (2-amino-6-bis[(2-chloroethyl)amino]-1,2,3,4-tetrahydro-2-naphthoic acid, **10**), DL-2-NAM-7 (2-amino-7-bis[(2-chloroethyl)amino]-1,2,3,4-tetrahydro-2-naphthoic acid, **11**), and DL-2-NAM-8 (2-amino-8-bis[(2-chloroethyl)amino]-1,2,3,4-tetrahydro-2-naphthoic acid, **12**).

For the elucidation of a common binding mode (CBM), flexible molecular docking was performed using five conformationally restricted analogs of phenylalanine **8**–**12** [33] (Figure 5) displaying variation in the ligand efficiency and fit quality [49] in LAT1 (Table 1). The ligands **9**–**12**, although chemically reactive due to the bis(2-chloroethyl)amino moiety, were not described to cause alkylation or impairment of the transport activity of LAT1. The replacement of the chlorine in **11** by hydroxy resulted in similar low V_max_ but increased K_m_, indicating that the NM was not alkylating LAT1 [34]. Thus, **9**–**12** are a motivating set of ligands to investigate LAT1 inhibition, represented by the common core scaffold **8.**

**Figure 6:**
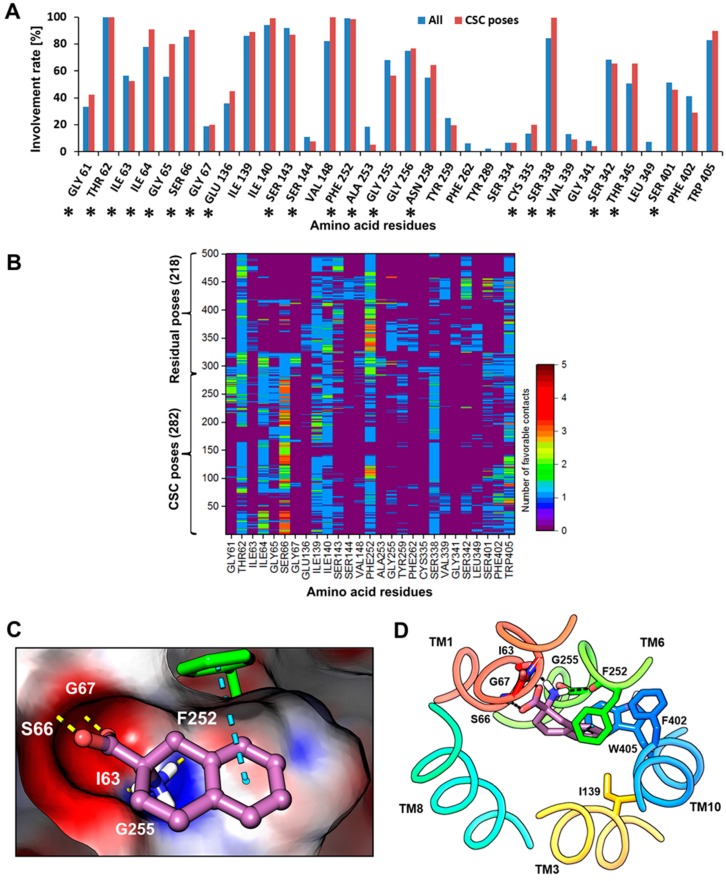
(**A**) The structural interaction fingerprint (SIFt) for all contacts in the docking poses, calculated with Schrödinger; All: 500 poses after docking, CSC poses: common scaffold cluster poses; The residues showing direct interaction with the docking poses are marked with an asterisk; (**B**) The SIFt showing the favorable interaction count (hydrogen bond + hydrophobic) in CSC and residual poses, excluding the polar interactions of chlorine; (**C**) Close-up front view of the predicted binding mode of **8** (violet) in LAT1. The ligand is depicted in stick-ball style; (**D**) periplasmic view of the binding mode of **8** (violet) in LAT1. The ligand and interacting residues are shown in stick representation. The dotted yellow lines in (**C**) and the black dotted lines in (**D**) show hydrogen bonds, while the dotted blue line in (**C**) and (**D**) indicates π−π interaction.

The ligands were prepared and docked into the model using Glide XP [50] (see Methods), and 100 poses per ligand were collected after post-docking minimization. Altogether, 500 poses were clustered according to the rmsd of the heavy atoms of **8**, resulting in 30 clusters (Figures S9 and S10). To follow the concept of a CBM, only those clusters were considered that comprise poses of at least four out of the five ligands used for docking (common scaffold clusters, CSCs) [51,52,53]. This led to the identification of 12 CSCs and 18 residual clusters corresponding to 282 poses (Figure S11) and 218 poses (Figure S12), respectively. The structural interaction fingerprint (SIFt) [54] analysis of the 500 poses showed that the residues located on TM1, TM3, TM6, TM8, and TM10 were primarily mediating the binding of the ligands (Figure S13). This tool identifies the amino acid residues that show polar or non-polar interactions with the docking poses or are situated in close proximity (5Å) to the ligand. The SIFt revealed that T62, I64, S66, I139, I140, S143, V148, F252, G256, S338, and W405 were the main residues showing >75% involvement in all 500 poses and 282 CSC poses (Figure 6A, Figures S14 and S15). The residues G61, I64, G65, S66, I139, S338, S401, F402, and W405 offered more favorable contacts in the CSC poses when compared to the residual poses (Figure 6B). After careful inspection of CSCs, docking poses with small average distance to the centroid from the highly populated and evenly distributed cluster 1 were selected for analyzing the protein-ligand interactions. The Spearman’s rank correlation coefficient between docking scores and pIC_50_ of **8**–**12** was 0.90. Alternatively, we also generated a network projection of the docking poses to visualize the global pose space. The poses from the densely populated cluster of the plot were examined by performing an interactive pharmacophore clustering (see SI, Figure S16). Interaction energy calculation for residues within 12Å of the grid center during docking provided a per-residue energy contribution to the binding, which is a combination of electrostatic and van der Waals energy. Among all amino acid residues considered, T62, I63, G65, S66, G67, S143, F252, A253, G255, G256, and W405 contributed >1 kcal mol^−1^ to the total interaction free energies in the docking poses of **8**–**12** (Figure S17). On the contrary, residues T71, P72, S96, S144, A201, K204, L208, I284, V285, L287, V288, V290, T292, F330, V331, and G332 offer unfavorable positive interaction free energies in the binding. The significant negative contributions to the binding through electrostatic interactions were attributed to residues positioned on the TM1 and TM6 helix-break with exposed carbonyl and amide groups forming the polar region or Amino acid Binding Site (ABS) of LAT1. Among the non-polar residues I139, I140, V148, F252, F402, and W405 stimulated the binding of the side chain through hydrophobic interactions. In the predicted binding mode of **8** (Figure 6C,D), the positively charged α−amino is donating hydrogen bonds to I63 in TM1, and F252 and G255 in TM6, while the negatively charged α−carboxyl group is accepting hydrogen bonds from S66 and G67 in TM1. The aromatic ring is occupying the Side-chain Binding Site (SBS) or Hydrophobic Sub-Pocket (HSP) of LAT1 (Figure 6D, Figure S19A) enclosed by the side chains of I139 (TM3), F252, F402 (TM10), and W405 (TM10), where it is engaged in an π−π interaction (edge to face) with the side chain of F252 (Figure 6D). In addition, the tetrahydronaphthalene moiety is engaged in favorable van der Waals interactions with residues of the HSP. Interestingly, MD simulation revealed an additional residue Y254 (TM6) involved in the HSP (Figure S18). Similar to **8**, **9**–**12** displayed conserved polar interactions of the amino acid moiety (Figure S19B–E), but a significant difference was noticed in the hydrophobic interactions of **9**–**12**. In **11**, the HSP is occupied entirely by the tetrahydronaphthalene and chloroethyl 1 moiety (Figure S19D), resulting in substantial hydrophobic interactions that may explain the high affinity of this isomer. In **9** and **10**, moderate to low interaction of the side chain with the HSP (Figure S19B,C) is consistent with the *in vivo* affinity trend of **9** > **10**. One chloroethyl in **12** is oriented towards the polar region and is in close contact with S338, which seems energetically unfavorable. In combination with the unfulfilled HSP and the steric clash of the NM with S338, this altogether provides an explanation for the low affinity of **12**. The other chloroethyl in **9**–**12** is oriented towards the open and wide region of moderate hydrophobicity and hydrophilicity where chlorine is engaged in a hydrogen bond interaction with N258 in **9**, T345 in **10** and **12**, and T345 and S143 in **11** (Figure S19B–E). This region is relatively large in size and located opposite to the SBS, indicating that it might function as an auxiliary binding site of LAT1. Overall, the results from the experimental data guided docking are in agreement with the SAR trend in **9**–**12**, indicating that the optimal position of the NM for affinity is C-7 (**11**) of the tetrahydronaphthalene ring [33], while isomers having the NM at C-6 (**10**) and C-8 (**12**) have more than 1000-fold, and with the NM at C-5 (**9**) have 100-fold lower affinity than the C-7 isomer.

### 2.3. Dynamic Pharmacophore Modeling

**Figure 7:**
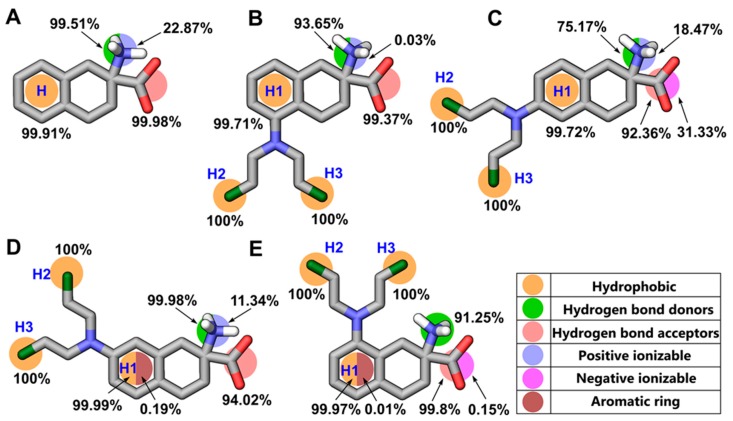
Dynamic pharmacophore map of **8** (**A**), **9** (**B**), **10** (**C**), **11** (**D**), and **12** (**E**) showing the overall occurrence percentages of pharmacophoric features calculated from 10000 equidistant snapshots of the corresponding Molecular dynamics (MD) trajectories.

**Figure 8:**
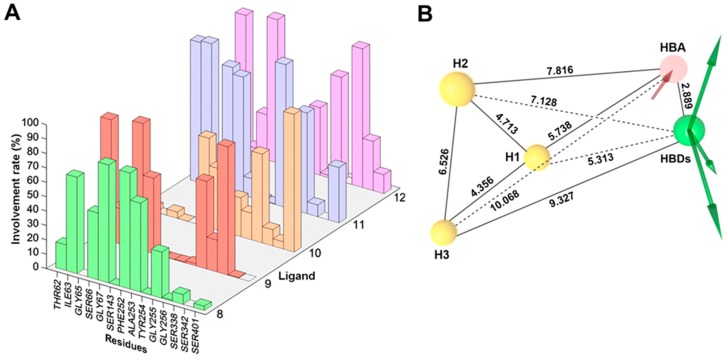
(**A**) Dynamic interaction fingerprint (DIFt) showing the percent involvement of residues in hydrogen bond interactions of **8** (green), **9** (red), **10** (orange), **11** (blue), and **12** (violet); (**B**) Three-dimensional (3D) spatial arrangement of pharmacophoric features of the dynamic pharmacophore model of **11** showing inter-feature distance (Å) constraints. The yellow spheres represent the hydrophobic features (H1, H2, and H3), while the green vectors are Hydrogen bond donor (HBDs) and the red vector is Hydrogen bond acceptor (HBA).

An integrated approach combining MD simulations and structure-based pharmacophore modeling was performed to investigate the real-time dynamics of the protein-ligand interaction. In the initial step, the conformational stability of the predicted structure of LAT1 and of the docking poses of **8**–**12** was assessed by analyzing the trajectories obtained in 20 ns simulations. For all five systems, the average rmsd of Cα and ligand was in a reasonable range (Figure S20). The MD trajectories were visually scrutinized to ensure that no substantial movement occurred and that the ligand stayed within the active site until the end of the simulation. In the next step, novel dynamic pharmacophores were generated for each 2 ps frame saved (see Methods). The plot of pharmacophore features evolution as a function of simulation time for **8**–**12** is shown in Figure S23. The dominant features discerned from the dynamic pharmacophores analysis include one hydrophobic feature, two Hydrogen bond donor (HBD), and three Hydrogen bond acceptor (HBA) in **8** (Figure S23A); three hydrophobic, one HBD, and three HBA in **9** (Figure S23B); three hydrophobic, one HBD, and one HBA in **10** (Figure S23C); three hydrophobic, two HBD, and two HBA in **11** (Figure S23D); and, three hydrophobic, two HBD, and three HBA in **12** (Figure S23E). The frequency count of pharmacophore features was assessed to determine the importance of individual features contributing to the active site interactions. Figure 7 shows the percent occupancy of different pharmacophore features observed in the MD simulation of LAT1 bound to **8**–**12**. The hydrophobic feature H in **8** corresponds to the aromatic ring, while hydrophobic features H1, H2, and H3 in **9**–**12** correspond to the aromatic ring and two chlorines of the NM side chain. The HBDs and HBAs correspond to the positively charged α−amino and the negatively charged α−carboxyl in **8**–**12**. The minor or infrequent features encountered in the simulation consists of an aromatic ring, a positive ionizable and a negative ionizable group. All major features (hydrophobic and hydrogen bond features) in **8**–**12** showed very high involvement rate (>70%), indicating a consistent interaction of the ligand with the residues throughout the simulation. The flexibility of the active site was monitored by computing the number of excluded volume of spheres (EVS) per frame that represents the positions sterically claimed by the protein residues. The average number of EVS did not vary significantly in **8**–**12** (17, 21, 18, 19, and 20, respectively), indicating that the curvature of the active site was conserved in all five systems. The dynamic interaction fingerprint (DIFt) of the MD trajectories was computed to determine the involvement rate of residues in hydrogen bond and hydrophobic interactions of **8**–**12**. The DIFt revealed that the major interaction partners involved in the manifestation of hydrogen bond features consist of G67 (82%) and F252 (80.5%) in **8**; T62 (84.5%) and S66 (88.2%) in **9**; F252 (42.9%) and G256 (61.6%) in **10**; S66 (86.5%), and G67 (82%) in **11**; S66 (99.5%) and S338 (96.3%) in **12** (Figure 8A). The DIFt for hydrophobic contacts showed that I147 (26.67%) and F252 (43.62%) were the major interaction partners in **8**; I139 (45.5%) and F252 (10.7%) in **9**; I139 (47.2%) and V148 (21.6%) in **10**; I140 (28%), and F252 (53.71%) in **11**; and, I139 (68.3%) and Y259 (38.8%) in **12**. Overall, the data obtained from the DIFt calculations were in agreement with the features occupancy rate determined from the dynamic pharmacophores.

### 2.4. Virtual Screening

**Figure 9:**
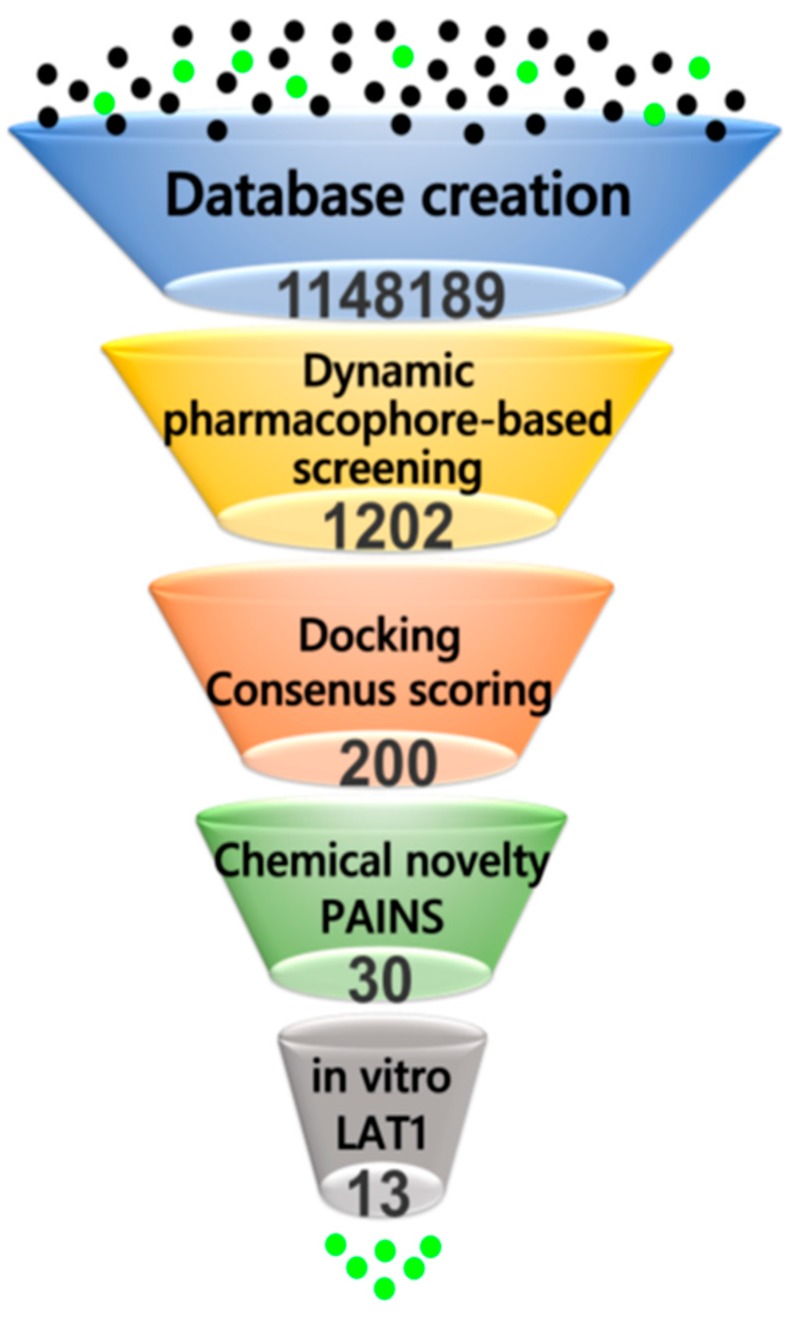
Schematic depiction of the virtual screening procedure.

**Figure 10:**
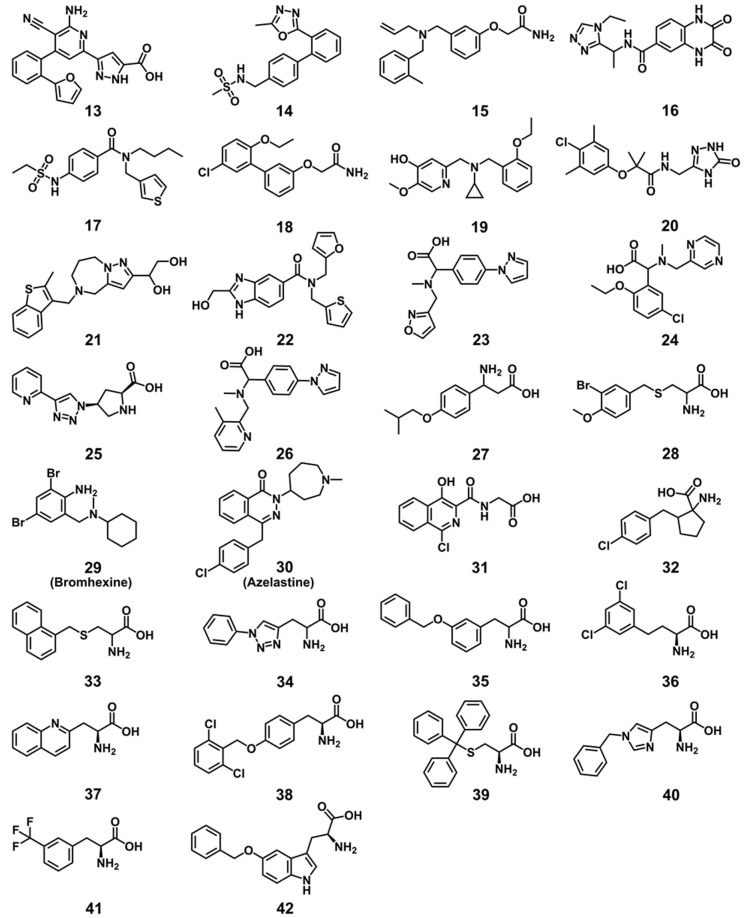
Compounds tested for LAT1 inhibition: Chembridge (**13**–**28**), DrugBank (**29**–**31**), Enamine (**32**–**36**), Sigma-Aldrich (**37**–**42**), and reference compound **1** (**Figure 1**).

To predict new small molecule ligands of LAT1, and to experimentally validate the binding poses and pharmacophore models obtained, we performed a combined pharmacophore- and docking-based virtual screening of libraries from DrugBank [55], Chembridge [56], Enamine [57], and Sigma-Aldrich (Figure 9). Databases were prepared and screened against the dynamic pharmacophore model of **11** (Figure 8B), and against 130 unique and distinct dynamic models of **8**–**12** (see Methods). The pharmacophore screening reduced the database to a large extent, because molecules had to match the pharmacophore features and they had to satisfy the spatial constraints of the binding site. In total, 1202 hits or 0.1% of the entire database were retrieved as a consequence of the pharmacophore search, which were then docked to LAT1 using GOLD [58]. 10 poses per hit were collected. The ChemPLP function was used as the primary scoring function, while the Goldscore function was utilized for rescoring. All of the compounds were successfully docked, and poses were sorted based on ChemPLP. The consensus score of hits was computed using three scoring functions: ChemPLP, GoldScore, and Pharmacophore fit score (see Methods). The hits were ranked from best to worst based on their consensus score, and from the top-ranked 200 hits or 0.01% of the database, 30 compounds **13**–**42** (Figure 10) were finally selected for experimental testing based on a visual analysis, pose similarity to the CBM, and chemical diversity of the compounds. In this process, we ignored many known ligands and the natural substrates of LAT1 that scored better than the final selected compounds. All of the compounds selected for the testing passed the pan assay interference (PAINS) filter for recurrent hitters [59]. 

### 2.5. Experimental Testing

**Figure 11:**
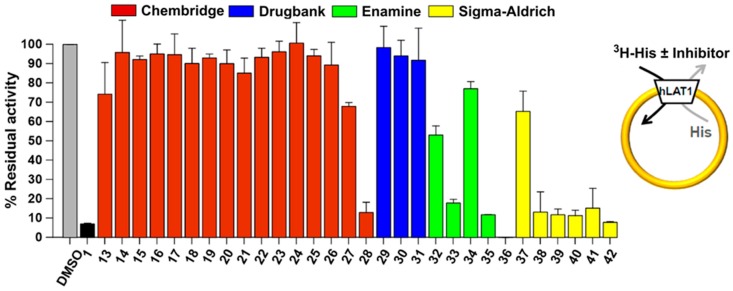
Inhibition by compounds of recombinant human LAT1 in proteoliposomes shown as percent residual activity of [^3^H]-His uptake at 100 μM concentration of the compound. Results are mean ± S.D. from three independent experiments.

**Figure 12:**
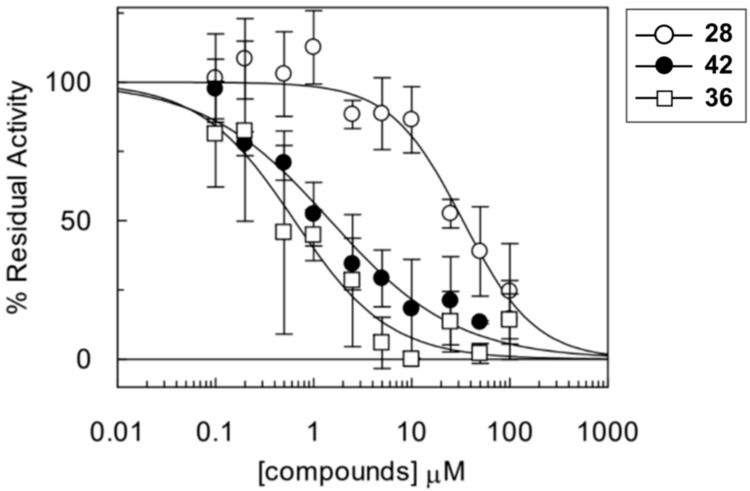
Dose-response curves of compounds **28**, **42**, and **36** for inhibition of the recombinant human LAT1 in proteoliposomes. Transport was assessed by adding 5 μM [^3^H]-His to proteoliposomes containing 10 mM His in the presence of different concentrations of the compounds. Results are mean ± S.D. from three independent experiments.

All 30 compounds were evaluated for their potency to inhibit LAT1 mediated histidine transport in proteoliposomes reconstituted with recombinant purified human LAT1 (see Methods) [43]. Compounds were measured at a concentration of 100 μM against **1** (BCH) as a positive control and dimethyl sulfoxide (DMSO) as a negative control. As illustrated in Figure 11, nine compounds, **28**, **33**, **35**, **36**, **38**–**42**, are highly active; one compound, **32**, is moderately active; and, three compounds, **27**, **34**, and **37**, are slightly active. Of these, two compounds, **36** and **42**, showed the complete inhibition of LAT1 (>90%) at 100 µM. We did not notice any effect in the presence of DMSO that was also used as a solubilizing agent, signifying that the inhibition was exclusively triggered by the compounds. The most effective compounds **28**, **36**, and **42** were chosen for dose-response analysis. As expected, compounds **36** and **42** led to a complete and robust inhibition with half maximum inhibitory concentration (IC_50_) values of 0.64 ± 0.12 and 1.48 ± 0.27 μM, respectively, and compound **28** yielded an IC_50_ of 33.2 ± 4.5 μM (Figure 12). Among these compounds, **36** is ~10 times and **42** is ~four times more potent than **1,** which has an IC_50_ of 6.8 ± 0.27 μM in the same test system, as determined previously by Napolitano and co-worker [43].

**Figure 13:**
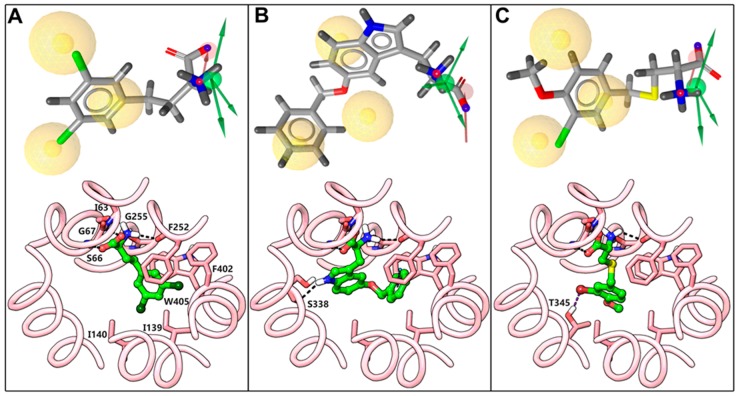
Pharmacophore fits and the predicted binding modes of **36** (**A**), **42** (**B**), and **28** (**C**). The pharmacophore features are colored according to Figure 8B, and the aligned ligands (grey) are depicted in stick style. In the docking pose below, ligand (green) and residues (red) are depicted in stick-ball and stick style, respectively. The dotted black lines indicate hydrogen bond interactions.

Figure 13 shows the pharmacophore fits, and the predicted docking poses of inhibitors **36**, **42**, and **28**. All three compounds fit perfectly with the HBA and the HBDs of the dynamic mode of **11**. However, they exhibited disparity in satisfying the hydrophobic features, H1, H2, and H3, of the dynamic model. Compound **36**, a butanoic acid derivative, owing to the presence of an extra carbon that increases the length of the side chain, overlaps almost perfectly with the aromatic ring (H1) and the extended chlorines (H2 and H3) of **11**, which could hardly be inferred through chemical resemblance measures. In **42**, the hydrophobic features, H2 and H3, correspond to two lipophilic aromatic rings that are equivalent to two chlorines of the NM side chain of **11**, signifying that the hydrophobic features need not necessarily to be halogens. Despite lacking the central hydrophobic feature H1, **42** is a potent ligand of LAT1. Hence, **42** offers unique chemistry, which could barely be inferred through chemical similarity techniques. Similarly, the pharmacophore depiction of compound **28** shows one missing hydrophobic element corresponding to H2, whereas H1 and H3 correspond to the aromatic ring and bromine. Though considerably less potent than **36** and **42**, **28** is a cysteine-based LAT1 inhibitor that would be difficult to retrieve through simple similarity-search using **11** or phenylalanine (for example). All three compounds showed conserved polar interactions of the α−amino and the α−carboxyl group. On the other hand, a pronounced difference was observed in the magnitude of their hydrophobic interactions. The 3,5-dichlorophenyl moiety in **36** is entirely occupying the HSP, resulting in considerable hydrophobic interactions that may explain the high potency of this compound. Additionally, the *meta*-substituted chlorine (3-Cl) seems to be involved in a halogen bond interaction with the backbone oxygen of N398. Similar to **36**, the benzyl ring of **42** is extended to the HSP and it interacts with the hydrophobic residues. Also, the ring is engaged in an π−π interaction with the side chain of F252 and the indole nitrogen of the tryptophan donating a hydrogen bond to the backbone of S338. In contrary to **36** and **42**, the benzylcysteine analog **28** is not interacting significantly with the HSP in the docking pose. The *meta*-substituted bromine is engaged in a hydrogen bond interaction with T345 and the methoxy at the *para* position is in good contacts with I139 and I140. The reduced hydrophobic interactions of **28** may explain the low activity of this compound as compared to **36** and **42**. Since pharmacophore models indicated that the amino acid moiety or charges at the α−carbon might not be necessary for binding, compounds **13**–**22** and **29**–**31** were tested. Albeit ranked within the top 50 molecules, these compounds did not show inhibition, indicating that the amino acid moiety is crucial for LAT1 recognition. This observation is consistent with previous studies, demonstrating that compounds lacking this chemical motif do not bind to LAT1 [4,5,30,32,60]. In line with this, the amino substituted compounds **23**–**26**, with a free α−carboxyl group, also failed to show inhibition, indicating the importance of a free α−amino group [4,46,60,61]. The α−amino group seems to be more crucial than the α−carboxyl group, as demonstrated by studies where substitution on the α−amino group completely abolished the activity [46,60,61]. We hypothesise that substitutions at the α−amino or α−carboxyl are sterically disallowed and they interfere with the binding of the polar head group, resulting in a low-affinity ligand binding (α−carboxyl substitution and a free α−amino group) or a complete failure of the ligand to exhibit binding (α−amino substitution and a free α−carboxyl group or substitutions on both groups). This hypothesis, supported by the docking poses, is in line with a CoMFA model of LAT1, which demonstrated that the addition of steric features beyond the amino acid terminal decreases the affinity [30]. However, studies have shown that the amino acid moiety is not a stringent requirement and that it is feasible to replace the alkoxy oxygen of the α-carboxyl group with a hydroxamic acid moiety, or to modify the α-carboxyl group to carboxylic esters, though with a decreased affinity for LAT1 [61,62]. Compound **27** with the amino group located at the β−carbon is slightly active, indicating that the optimal position of the amino group is at the α−carbon atom. This observation is consistent with previously reported results that the spacing between the positive and the negative charge should not surpass ~3Å. If the amino group is positioned distant from the carboxyl group, then the compound loses affinity [32]. The rigid amino acid **32** showed modest affinity, indicating that the conformational restriction or a substitution at the α−carbon is tolerated, which is consistent with the previously reported results [46,61,63]. In terms of rigidity, the conformational restraint that is induced by the cyclohexane seems more favorable than the cyclopentane or other carbocycles for LAT1 activity, as compound **8** has been reported to be superior to the related indane and the bicyclo heptane analogs [33]. Compound **34** was slightly active, possibly because of the polar triazole ring, resulting in diminished hydrophobic interactions. This suggests that hydrophobicity is playing an important role in the binding of the side chain [64,65]. Interestingly, a recent study demonstrated that LAT1 is capable of binding and transporting ligands with polar and ionizable moieties substituted at the *meta* position of phenylalanine, suggesting the promiscuous nature of the binding site [63]. The quinoline compound **37** performed better than **34**. Other compounds **33**, **35**, **38**–**41** were highly active and showed inhibition in the range of 80–90%. The docking geometries of all active compounds were consistent with the putative CBM. To further assess the novelty of the compounds that were retrieved in this study, the chemical similarity or dissimilarity of the compounds was calculated by comparing their chemical fingerprints (MACCS, Radial ECFP, and FP2) with those of the reference compound **1** and the potent LAT1 inhibitors **2** and **11** using the Tanimoto coefficient (Table S6). The similarity calculations revealed that all compounds are chemically unrelated to **1**, **2**, and **11,** except compound **32,** which showed a Tanimoto similarity of 0.75 with **1**, on the basis of MACCS keys. The results that were obtained demonstrate that the pharmacophore-based screening was impartial and performed autonomously from the chemical resemblance.

## 3. Discussion

**Figure 14:**
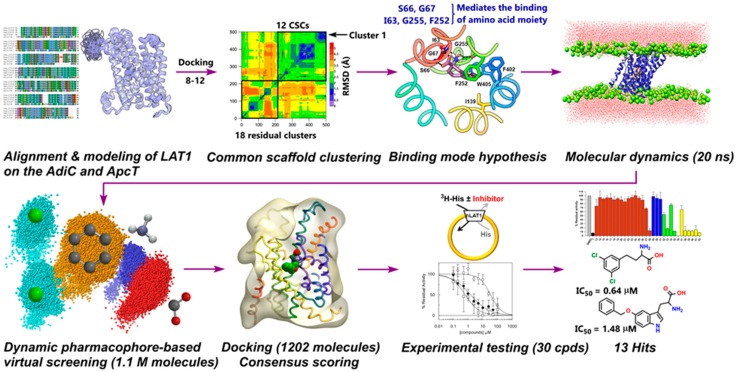
Structure-based workflow for discovering potent inhibitors of LAT1. Workflow shows LAT1 homology model construction (top left) followed by docking of **8**–**12** into the model. The common binding mode (CBM) was investigated using an in-house developed method called “common scaffold clustering”. The putative binding modes of **8**–**12** were subjected to MD simulation (20 ns) followed by generation of dynamic pharmacophores. Then, ~1.1 million molecules were virtually screened against the dynamic models to predict new ligands. Further docking of hits and subsequent testing of the 30 top-ranked molecules led to the identification of 13 hits. The most potent molecules discovered in this study show IC_50_ values of 0.64 μM and 1.48 μM, respectively.

Among L-type amino acid transporters, LAT1 is noticeably expressed in human cancers, where it provides vital amino acids for growth and proliferation. Therefore, the inhibition of LAT1 activity is considered as an attractive goal for the treatment of cancer. With the increasing availability of the X-ray structures of transmembrane transporters, homology modeling, in combination with molecular docking, has been successfully used in the discovery of novel ligands for various protein targets of the SLC family [66,67,68,69,70,71]. Additionally, these computational models have shown their significance in the elucidation of the molecular basis of drug transporter interaction for targets of therapeutic interest [51,52,53,72]. We describe here a successful structure-based design approach that led to the identification of potent new inhibitors of LAT1, with an unprecedented hit-rate. However, it should be noted that the different performance of the compound libraries in providing active hits might be due to a different abundance of amino acids in the Enamine and Sigma-Aldrich libraries. An outline of the procedure followed in this study is shown in Figure 14.

The homology model of LAT1 was developed in two different conformations: outward-occluded and inward-open. The predicted structure of LAT1 provided intriguing insights into the substrate binding site. LAT1 shares no more than 20% sequence identity with AdiC and 23% with ApcT, and its binding site differ significantly from its closest homologs and canonical system L members. However, the sequence identity for residues around the substrate binding site is 40%, which seems reasonable for investigating transporter-ligand interactions and performing structure-based screening while using a homology model. The binding site of LAT1 is centrally located and well organized with favorable hydrophilic and hydrophobic regions that mediate the binding of the amino acid moiety and the side chain. Docking poses of **8**–**12** disclosed that the hydrogen bond interactions of the amino acid moiety are preserved between LAT1 and AdiC, and that the residues located on TM1 and TM6 are critical for binding. Especially, I63, F252, and G255 are responsible for binding of the positively charged α−amino group, and S66 and G67 of the GSG motif mediate the binding of the negatively charged α−carboxyl group through backbone hydrogen bond interactions. Additionally, the side chains of residues S66 and S338 are involved in electrostatic interactions with the α−carboxyl group. The side chain binding of the ligand involves hydrophobic interactions, with aromatic F252, F402, W405, and aliphatic I139 and I140. The corresponding residues in AdiC include I23, W202, and I205 interacting with the α−amino group of arginine, and S26 and G27 interacting with the α−carboxyl group. The side chain interactions in LAT1 are analogous to the hydrophobic interaction of the aliphatic portion of arginine with M104, W202, and I205 of AdiC [24]. 

The putative HSP in LAT1 is consistent with a previous study, where the *meta* substitution on the phenylalanine or tyrosine with lipophilic groups are believed to occupy this pocket leading to high affinity and improvement in the substrate activity [28,63]. The HSP may explain the high affinity of **11,** where one of the chloroethyl side chains as well as the tetrahydronaphthalene moiety occupy this pocket, leading to massive hydrophobic interactions. Other NMs showed variable interaction with the HSP, which was consistent with their biological activity. Similar to **11**, the high affinity of **2** and **7** can be attributed to the *para*-substituted lipophilic moiety, which can swing around the flexible O and CH_2_ groups to assume a *meta* conformation and satisfy the HSP [4]. Additionally, through intensive docking simulations, we propose that ligands that do not show strong interaction with the HSP are probably substrates, while those that satisfy this pocket are blockers. Based on the results from docking studies, hydrogen bond interactions and hydrophobic interactions seems to be principal driving forces for strong ligand binding in LAT1. This observation is consistent with the binding energy calculations of the MD trajectories, where electrostatic and van der Waals energy contributed substantially to the binding of **8**–**12** (see SI). 

An exhaustive sampling of the docking poses of **9**–**12** revealed new residues never predicted before to exhibit interactions with the halogen atoms. Briefly, S401 and E136 seem to be halogen bond acceptors, while S143, N258, and T345 are hydrogen bond donors to the halogens (Figure S15). Given the widespread occurrence of the halogens in known LAT1 ligands, such as T_4_, T_3_, reverse T_3_, 3,3′-diiodothyronine (3,3′-T_2_) [46,47], 3-iodotyrosine, 3,5-diiodotyrosine, Fenclonine [5], 2-NAM’s [33], it seems likely that the halogen in these ligands not only modulate the lipophilicity of the compound, but also participate in polar interactions, hence contributing to the binding affinity. Additionally, a recent study demonstrated that the *meta*-substituted halogens exhibit an inhibition trend I > Br > Cl > F in phenylalanine and Br > Cl = F > I in tyrosine [28], possibly indicating halogen interactions in these analogs with the binding site residues of LAT1. Interestingly, Krause et al. have alleged halogen interactions that can justify the high affinity of T_3_ and 3,3′-T_2_ in LAT2 [73]. Both LAT1 and LAT2 have aromatic and hydrophilic residues in their binding site that can function as halogen bond acceptors and hydrogen bond donors to the halogens. Interestingly, out of 13 actives that were identified in this study, five compounds, **28**, **32**, **36**, **38**, and **41**, contain halogens. This might indicate that the halogens and associated interactions could be utilized as a molecular tool in the rational design of LAT1 inhibitors by selectively introducing I, Br, or Cl into the aromatic ring.

Napolitano and co-workers have demonstrated that F252A substitution entirely abolishes the transport activity of LAT1, indicating that F252 is serving as a proximal gate that is responsible for occlusion of the substrate from the periplasm [27]. C335A, the double mutant C335A/C407A, and S342G mutants exhibited reduced transport, while C407A displayed similar transport activity to the wild-type [27]. Our interaction energy calculations are consistent with the mutagenesis data where F252, C335, and S342 favored the binding, while C407 did not show any interactions with the docking poses (Figure S17). 

By incorporating dynamics into the structure-based pharmacophores, we identified key pharmacophoric features of **8**–**12** that are involved in binding. The pharmacophores generated from the MD trajectories represent the conformational flexibility of the ligand and the active site. This conformational difference has been considered as a main benefit of the dynamic models when compared to the static model developed from a putative binding mode or from an experimental structure for analyzing protein-ligand interactions [74]. The dynamic pharmacophores analysis revealed that the three hydrophobic (H1, H2, and H3), two HBA, and two HBD were the dominant features that developed during the MD simulation of **11** bound to LAT1. Similar to **11**, other NMs showed very high occupancy of the major features, implying that together hydrophobic and hydrogen bond features are implicated in the binding of the NMs.

Encouraged by the results from retrospective screening of models (see Methods), we set out to predict new ligands of LAT1 through dynamic pharmacophore-based virtual screening followed by docking of the retrieved hits. We utilized consensus scoring for grading the compounds to avoid giving preference either to the pharmacophore fit score or to the docking score. Thirteen previously unknown ligands were identified that inhibited the [^3^H]-His_ex_/His_in_ antiport activity of LAT1 in proteoliposomes. Notwithstanding the deficiency of a cellular environment, the proteoliposomes based assay offers the benefit of detecting activity solely attributed to the transport protein remodeled [23]. Among the hits identified, compounds **28**, **33**, **35**, **36**, **38**–**42** exhibited inhibition in the range of 80–100%. Indeed, the best of them, compound **36** and **42,** elicited almost complete inhibition. The dose-response analysis of compounds **36**, **42**, and **28** yielded an IC_50_ of 0.64 μM, 1.48 μM, and 33.2 μM, respectively. While the most potent inhibitor of LAT1 KYT-0353 **2** is a rationally designed tyrosine analog, compound **36** is a potent ligand identified through a structure-based study. Collectively, these results support the proposed binding mode and demonstrate the successful authentication of an interactive pharmacophore model with 3 hydrophobic, 3 HBD and 1 HBA features. In other words, it can be surmised that the molecular features of 2-NAM-7 (**11**), in the context of their spatial arrangement in the binding site, are critical for LAT1 affinity. By translating these molecular interaction features, derived from an MD snapshot, into a pharmacophore model and using it in virtual screening, we were able to identify potent, non-alkylating, and chemically diverse ligands of LAT1, which could hardly be deduced through similarity-based search utilizing an NM. Our proposed structure-based model corroborates the ligand-based model, comprising of HBD, negative ionizable, aromatic, and HBA features, as reported by Ylikangas et al. [32]. However, HBA next to the aromatic feature in the ligand-based model may not be an essential feature to gain affinity *via* hydrogen bond interaction. Docking poses of **2**, **4**–**7**, **9**–**12**, **35**, **38,** and **42** did not show any hydrogen bond interaction of the tertiary amine or oxygen, indicating that the carboxyl, amide, or oxygen at the *meta* or *para* position likely plays a role of the linker to anchor lipophilic bulk or a parent drug, which can engage in hydrophobic interactions. The failure of compounds **13**–**22** and **29**–**31** to inhibit LAT1 demonstrates that the amino acid moiety is an indispensable feature and a foremost requirement of ligand recognition. Similarly, compounds bearing substitutions either at the α−amino group cannot bind, as exemplified by the inactive compounds **23**–**26**, which are substituted at the α−amino group. Despite being enabled with a positive and a negative charge, **23**–**26** failed to show inhibition, indicating that the charges at the α-carbon are not a stringent requirement for ligand binding [62]. Instead, five hydrogen bond interactions of the amino acid moiety with the backbone, as per the putative CBM (Figure 6C,D), seems obligatory for LAT1 recognition so as to achieve effective binding. Our hypothesis of steric hindrance at the amino acid binding site fits well with the experimental outcome of **23**–**26**. However, alteration of the length and steric bulk of the side chain is permitted, as demonstrated by several compounds that strongly inhibited LAT1. The hydrophobicity of the side chain seems to be decisive in achieving affinity *via* hydrophobic interactions while considering an equivalent enthalpic gain from the binding of the amino acid moiety. The inhibition trend **35** > **38** suggests that *meta* substitution on the phenylalanine or tyrosine is more favorable for affinity than *para*. This observation is consistent with those reported previously where *meta-*substituted analogs of phenylalanine showed increased LAT1 activity [28,30,32,63] and more significant rat brain uptake relative to the analogous *para* compounds [30,75]. As another example, m-Sarcolysin has 100 times higher affinity for LAT1 when compared to melphalan, indicating a marked preference for *meta*-substitution on the aromatic ring of phenylalanine [34]. Among the hits that were retrieved, compound **28**, **33**, and **39** are benzylcysteine analogs. Captivatingly, benzylcysteine is a known inhibitor of Alanine-Serine-Cysteine transporter (SLC1A5, ASCT2) [76]. Since LAT1 and ASCT2 are ~35% homologous, it may well be possible that these analogs show ASCT2 inhibition. Acivicin is an excellent example that displays drug polypharmacology. It inhibits both LAT1 and ASCT2, as well as other metabolic enzymes to curb the growth of cancer cells [77,78]. This might indicate that LAT1 and ASCT2 share specific common molecular features that recognize ligands, such as acivicin. Also, the recently identified 1,2,3-dithiazole analogs are reported to be dual inhibitors of LAT1 [23] and ASCT2 [79].

In conclusion, we constructed a homology model of LAT1 based on the atomic structures of prokaryotic homologs. Subsequently, we performed molecular docking, MD, and interactive pharmacophore modeling that enriched our knowledge of the structural and molecular basis of LAT1 inhibition. Four commercially available small molecule libraries were computationally screened, and 30 top-ranked hits were tested experimentally. 13 novel ligands were identified. Taken together, these findings have once again demonstrated that AdiC is a reliable template for structure-based ligand discovery until the experimental structure of LAT1 or a new structure of high sequence identity becomes available. Two of the retrieved compounds, **36** and **42**, are potent inhibitors of LAT1 with an excellent physicochemical profile, which makes them promising candidates for further evaluation. In addition, these compounds will broaden the scope of future SAR studies that may guide the rational design and synthesis of new inhibitors of LAT1. Finally, our validated structure-based methodology could be implemented in drug discovery campaigns targeting other biologically essential transporters.

## 4. Methods

### 4.1. Homology Modeling

Homology modeling of LAT1 was performed using MODELLER v9.14 [80]. The models were constructed against two templates: (i) the crystal structure of the arginine/agmatine transporter AdiC from *E*. *coli* (PDB ID: 3L1L), (ii) the crystal structure of ApcT from *M*. *jannaschii* (PDB ID: 3GIA). The initial alignment was generated using T-Coffee [81] that comprised LAT1 sequences of 4 different species, human (UniProt accession number: Q01650), mouse (Q9Z127), rabbit (Q7YQK4), dog (K0J2W9), and the template protein (Figures S1 and S2). We utilized the previously reported alignments [5,25,27,82] and the alignment that was obtained from T-Coffee, where gaps that were initially present in the TMs were moved to nearby loops to anchor highly conserved residues in each α−helices. In spite of manual correction, some gaps were observed in our final alignment (see SI). 50 models were generated on each template, and all of the models were assessed on the basis of normalized DOPE score. In the final model 14 loops (Residues: 64–66, 79–83, 110–115, 158–168, 217–242, 254–258, 263–272, 306–310, 334–337, 356–376, 389–394, 419–433, 450–453, and 473–479) were optimized by using the loop refinement module of Schrödinger. The side chains of residues of the binding site were minimized by keeping the backbone constrained while using OPLS-2005 forcefield [83]. 

### 4.2. Molecular Docking 

All ligands used in docking studies were built in the L-configuration in maestro. The low-energy conformations of ligands were generated using LigPrep [84]. Protonation states were computed at target pH 7.0 ± 2.0 and verified with the major microspecies calculated using MarvinSketch [85]. Thirty-two stereoisomers were computed for each ligand by retaining specified chiralities. The homology model of LAT1 was prepared for docking simulations by using the Protein Preparation Wizard workflow [86]. Hydrogen atoms were added and bond orders were allocated. All heavy atoms in the protein were minimized to rmsd 0.3Å using OPLS-2005. The docking grid was generated by using the predicted binding site residues. Flexible molecular docking was performed using Glide XP [50,87,88] with default parameters. The scoring function Glidescore was considered for the binding affinity prediction. 100 poses per ligand were collected to ensure the convergence of conformational sampling.

### 4.3. Cluster Analysis

For the elucidation of CBM, a rmsd matrix of 500 poses was generated on the basis of the common scaffold and clustered using the complete linkage algorithm with a clustering height of 2.0Å. Only those clusters were considered that comprised of poses of at least four out of the five ligands docked. Clustering in this study was performed by using the cheminformatics tool of Schrödinger.

### 4.4. Molecular Dynamics Calculations

Molecular dynamics (MD) simulation (20 ns) of the protein-ligand complex (**8**–**12**) was performed in GROMACS 5.0.5 [89] while using the united atom GROMOS 54a7 force field. The complex was fixed in a fully solvated Dipalmitoylphosphatidylcholine (DPPC) membrane and ions were added to neutralize the system. Long-range electrostatic interactions were estimated using the particle mesh Ewald method with a 0.16 nm cutoff for the real space calculation [90]. To address the van der Waals interactions, a cutoff of 1.2 nm was applied. The production run was performed at a constant temperature, pressure, and a number of particles (NPT). The Nosé-Hoover thermostat [91,92] was used at 323 K with a coupling constant τ = 0.5 ps, and the pressure was controlled using Parrinello-Rahman barostat at 1 bar [93]. The timestep for integration was 2 fs, and all bond lengths were restrained by using the LINCS algorithm [94]. Coordinates and velocities were saved every 2 ps. The rmsd values were obtained from a least-squares fit over the Cα atoms of the protein while using the gmx rms utility of GROMACS. 

### 4.5. Structure-Based Pharmacophore Modeling

The structure-based pharmacophore models were generated for each simulation frame saved using Ligandscout 4.09.2 [95] with default settings. The resulting interactive pharmacophore models of **8**–**12** were evaluated by noticing the ligand atoms that were involved in the pharmacophore feature and the type of feature. If a particular feature and the involved ligand atoms are identical in two models, then the incidence count of this feature was incremented [96]. In this way, we obtained occurrence frequency (statistical behavior) of a specific feature during the course of the simulation. Similarly, the occurrence pattern (statistical pattern) of the pharmacophoric features was monitored. The pharmacophore features appearing redundantly over time and sharing common feature type (e.g., hydrophobic, HBA, or HBD), and the same atoms on the ligand site were classified as major features, while those occurring infrequently were treated as minor features. The DIFt analysis was performed in Schrödinger to determine the interaction partners of LAT1 that led to the manifestation of hydrophobic and hydrogen bond features. The occurrence rate and occurrence pattern of the interactions partners were monitored.

### 4.6. Pharmacophore Clustering and Validation

Dynamic pharmacophores were clustered using the radial distribution function (RDF) code similarity algorithm with default settings (similarity set to 0.5; Cluster distance calculation method set to average) in Ligandscout [95]. For the selection of a dynamic model for screening, enrichment calculations were performed against the representative pharmacophore model (RPM) of the 10 most populated pharmacophore clusters of **8**–**12** (Figure S24). 97 known LAT1 ligands were collected from the literature [5,28,30,32,46,75,97], and 5300 decoys were generated using the Directory of Useful Decoys Enhanced (DUD-E) server [98]. This set of 5397 molecules were then screened against each RPM. The performance of the models was assessed by computing receiver operating characteristics (ROC) graphs and calculating the area under the curve (AUC), and robust initial enrichment (RIE) values of the enrichment plots, demonstrating the ability of the virtual screening to differentiate known ligands among the set of decoys The RPMs of **9**, **10,** and **11** performed equally well with AUC >0.73 (Figure S25, Table S4). The RPMs of **12** showed AUC between 0.65–0.75, while models of **8** had AUC <0.67. The RPM6 of **11** which obtained an AUC value of 0.74 and the highest robust initial enhancement (RIE) value of 9.41 (Table S4), indicating best screening performance was chosen for productive virtual screening. We additionally selected 130 distinct RPMs from the dynamic pharmacophores of **8**–**12** for carrying out screening via the common hits approach (CHA), as described previously [99]. 

### 4.7. Virtual Screening

All small molecules in the libraries, DrugBank (*n* = 6655), Chembridge (*n* = 1,044,986), Enamine (*n* = 96,000), and Sigma-Aldrich (*n* = 548), were converted into three-dimensional (3D) structures by using the Icon tool of Ligandscout [95]. 25 conformations per molecule were computed by using the Icon fast option with the energy window parameter and the rms threshold set to 15 kcal mol^−1^ and 0.5, respectively. The pharmacophore-based virtual screening was performed by using the iscreen module of Ligandscout [95], with default settings as described in the user manual. Molecules matching n or n − 1 query features were considered as hits, where n is the number of pharmacophore features in a model. The number of omitted features was set to 1. The exclusion volume spheres were checked to take into account the steric constraints of LAT1. 1202 hits were retrieved through the pharmacophore search which were then docked to LAT1 using GOLD [58] for further prioritization. Finally, a consensus score was calculated that combines the docking scores and pharmacophore fit score, as shown in Equation (1). Out of 30 compounds that were selected for experimental testing, 10 compounds **13**–**22** correspond to screening from CHA, while **23**–**42** (Figure 10) originated from screening against RPM6.

(1) $$\mathrm{Consensus}\;\mathrm{Score}=\;\frac{\mathrm{ChemPLP}\;\mathrm{Score}-{\;\mathrm{ChemPLP}\;\mathrm{Score}}_{\min}}{{\mathrm{ChemPLP}\;\mathrm{Score}}_{\max}-{\;\mathrm{ChemPLP}\;\mathrm{Score}}_{\min}}\;+\frac{\mathrm{GoldScore}-{\mathrm{Goldscore}}_{\min}}{{\mathrm{GoldScore}}_{\max}-{\;\mathrm{GoldScore}}_{\min}}\;+\frac{\mathrm{Pharmacophore}\;\mathrm{Fit}\;\mathrm{Score}-\mathrm{Pharmacophore}\;\mathrm{Fit}\;{\mathrm{Score}}_{\min}}{\mathrm{Pharmacophore}\;\mathrm{Fit}\;{\mathrm{Score}}_{\max}-\mathrm{Pharmacophore}\;\mathrm{Fit}\;{\mathrm{Score}}_{\min}}$$

### 4.8. Compounds Filtering and Similarity Calculations 

The primary checking of final selected compounds for PAINS was performed using the web service http://zinc15.docking.org/patterns/home/ [59] and returned no problematic compounds. Similarity calculations were performed in Canvas [100] and OpenBabel 2.4.1 [101]. All retrieved hits were compared to **1**, **2**, and **11** using MACCS, radial ECFP, and FP2 keys, and the Tanimoto coefficient (Figure S6). Hits with Tanimoto value <0.8 [102] on the basis of the MACCS fingerprint were considered as chemically dissimilar from **1**, **2**, and **11**.

### 4.9. Chemicals

The purchased compounds **13**–**28**, **32**–**42** were reported with a purity between 90–100% [(LC-MS or certificate of analysis (CoA)] and the identity was confirmed using ^1^H-NMR (Table S8, Figures S26–S69). The compounds **29**, **30**, and **31**, which were purchased from Sigma-Aldrich, were reported with a drugBank purity of ≥98% (HPLC) and the identity was confirmed using ^1^H-NMR as per CoA (spectrum not included in SI). We, in addition, measured ^1^H-NMR of compounds **13**–**26** because the vendor could not provide them (see SI). Compounds **36**–**42** were in an L-configuration, and compounds **27**, **28**, **33**–**35** were in the DL-configuration. The configuration of compound **25** is 2S,4S. The configuration of compound **32** is unknown.

### 4.10. Pharmacological Testing

#### 4.10.1. Purification and Reconstitution of Human LAT1

Human LAT1 was over-expressed in *E*. *coli* and was purified as previously described [43]. In brief, solubilized cell lysate was centrifuged (12,000× *g*, 10 min, 4 °C); supernatant was applied on a His-Trap HP column (5 mL Ni Sepharose) connected to ÄKTA start system. The column was equilibrated with 10 mL buffer (20 mM Tris HCl pH 8.0, 10% glycerol, 200 mM NaCl, 0.1% sarkosyl) prior to supernatant application. The column was washed with 10 mL buffer (20 mM Tris HCl pH 8.0, 10% glycerol, 200 mM NaCl, 0.05% DDM, and 2 mM DTE) and the protein was eluted with the same buffer plus 400 mM imidazole. Purified protein was desalted, in order to remove imidazole, using a PD-10 column in a buffer containing 20 mM Tris HCl pH 8.0, 10% glycerol, 0.05% DDM and 10 mM DTE.

The purified human LAT1 was reconstituted by detergent removal approach using the batch-wise method, as previously described [43]. In brief, the initial mixture contained: 4 μg of purified protein, 100 μL of 10% C12E8, 100 μL of 10% egg yolk phospholipids (*w*/*v*) in the form of liposomes that were prepared as previously described [103], 20 mM Tris HCl pH 7.5, and 10 mM L-His in a final volume of 700 μL. This mixture was incubated with 0.5 g of Amberlite XAD-4 for 90 min at 23 °C, 1200 rpm to retain detergent.

#### 4.10.2. Transport Measurements

To perform transport assays, the external substrate was removed by gel filtration chromatography: 600 μL of proteoliposomes were passed through a Sephadex G-75 column (0.7 cm diameter × 15 cm height) pre-equilibrated with 20 mM Tris HCl pH 7.5 and a concentration of sucrose to balance internal osmolarity. Transport was started adding 5 μM [^3^H]-His to proteoliposomes and stopped after 30 min according to the stop inhibitor method, as previously described [43]. In brief, in controls 1.5 µM HgCl_2_ was added at time zero and radioactivity was subtracted to samples for calculating net transport. After stopping the transport reaction, 100 μL of proteoliposomes was subjected to gel filtration chromatography using a Sephadex G-75 column (0.6 cm diameter × 8 cm height), to separate the external from the internal radioactivity. Proteoliposomes were eluted with 1 mL 50 mM NaCl in 4 mL scintillation mixture and counted. Initial transport rate was measured by stopping the reaction after 30 min and data were analyzed using Grafit software for IC_50_ values fitting. Protein concentration was estimated by Chemidoc imaging system1 [104] to calculate the human LAT1 specific activity.

## Figures and Tables

**Table 1:** Activities of the docked ligands (HAC: heavy atom count; LLE: lipophilic ligand efficiency; LE: ligand efficiency; FQ: fit quality).

Ligand	pIC_50_	LogP *o*/*w*	HAC	LE	LLE	FQ
**8**	5.11	−0.78	14	0.37	5.89	0.60
**9**	5.07	1.30	21	0.24	3.76	0.53
**10**	4.17	1.18	21	0.20	2.98	0.44
**11**	7.10	1.40	21	0.34	5.70	0.75
**12**	3.60	1.42	21	0.17	2.17	0.37

## Supplementary Materials

The following are available online at http://www.mdpi.com/1422-0067/20/1/27/s1. The homology model of LAT1 in the outward-occluded conformation and in the inward-open, occluded conformation, and the docking pose of ligand **8** to LAT1 (PDB); Additional studies and figures demonstrating homology modeling, model evaluation, docking, binding free energy calculations, compound inhibition, dose-response analysis, and purity data (PDF).

## Abbreviations

LAT1, large neutral amino acid transporter 1; APC, amino acid/polyamine/organocation; NM, nitrogen mustard; BCH, 2-Amino-2-norbornanecarboxylic acid; mTOR, mechanistic/mammalian target of rapamycin; HAT, heteromeric amino acid transporter; BBB, Blood-Brain Barrier; CBM, common binding mode; 3D QSAR, three-dimensional quantitative structure-activity relationship; MD, molecular dynamics; HBD, hydrogen bond donor; HBA, hydrogen bond acceptor; RDF, radial distribution function; RPM, representative pharmacophore model; ROC, receiver operating characteristics; AUC, area under the curve; MACCS, molecular access system; PAINS, pan assay interference compounds
