# Supplementary material for: Discovery of Potent Inhibitors for the Large Neutral Amino Acid Transporter 1 (LAT1) by Structure-Based Methods
Source: Int J Mol Sci. 2018 Dec 21;20(1):27. doi: 10.3390/ijms20010027 (PMC6337383; doi:10.3390/ijms20010027)
Supplement: Supplementary file 1 [file ijms-20-00027-s001.pdf]

Supporting information

Discovery of potent inhibitors for the large neutral amino acid transporter 1 (LAT1) by structure-based methods

*Natesh Singh,[1] Mariafrancesca Scalise,[2] Michele Galluccio,[2] Marcus Wieder,[1] Thomas Seidel,[1] Thierry Langer,[1] Cesare Indiveri,[2] Gerhard F. Ecker[1, *]*

[1]University of Vienna, Department of Pharmaceutical Chemistry, Althanstrasse 14, 1090 Wien, Austria
[2]Department DiBEST, Unit of Biochemistry & Molecular Biotechnology, University of Calabria, Arcavacata di Rende, Italy

Table of contents

Homology modeling of LAT1 2
Model evaluation 2
Network visualization of the poses 2-3
Binding free energy calculations 3-4
References 4-5
Table S1 6
Figure S1 7
Figure S2 8
Table S2 and Figure S3 9
Figure S4 10
Figure S5 11
Table S3 12
Figure S6 13
Figure S7 and S8 14
Figure S9 and S10 15
Figure S11 and S12 16
Figure S13 and S14 17
Figure S15 and S16 18
Figure S17 and S18 19
Figure S19 20
Figure S20 22
Figure S21 23
Figure S22 24
Figure S23 25-29
Figure S24 30
Figure S25 31
Table S4 32
Table S5 33
Table S6 34
Table S7 35
Table S8 36
LC-MS and ^{1}H-NMR spectrum of compounds 37-78

Homology modeling of LAT1

The homology model of human LAT1 was constructed against two templates: (i) the crystal structure of outward-occluded conformation of arginine/agmatine transporter AdiC from *E. coli* (PDB ID: 3L1L), [1] (ii) the crystal structure of inward-open conformation of ApcT from *M. jannaschii* (PDB ID: 3GIA) [2]. The sequence identity and the sequence similarity of LAT1 with AdiC is ~ 20% and ~ 40%, and the sequence identity and the sequence similarity of LAT1 with ApcT is ~ 23% and ~ 41%. The amino acid residues 1−50 and 480−507 of LAT1 were not considered in the model building because these residues are predicted to form long intracellular N- and C-terminus domains [3]. In our final alignment, short insertions of one and two amino acids were observed in the TM3 and TM11 of LAT1 (**Figure S1**). Gaps with deletions of four and one amino acids were found in the TM9 and TM10. Long insertions and deletions were observed in the extracellular loop 3 (EL3) between TM5 and TM6, undoubtedly implying ambiguity in the loop prediction. Additionally, the amino acid residue differences were observed in the TMs of LAT1 of human, mouse, rabbit, and dog (**Table S1**). However, the residues enclosing the binding site of LAT1 were identical in all species.

Model evaluation

The final model of LAT1 was evaluated using the PROCHECK [4] and QMEAN [5]. The Ramachandran analysis showed that 88.4% of all residues were present in most favored regions, 9.5% in additionally allowed, 1.6% in generously allowed and 0.5% in disallowed areas (**Table S2**, **Figure S3**). Most of the residues located in generously and disallowed regions were found on the outer surface and in the intra- and extra-cellular loops of the model. Only two residues G65 and G256 found in the forbidden areas were within 5Å of the binding site (**Figure S4**). Both residues were optimized *via* energy-based refinement using the variable dielectric surface generalized Born solvation model [6]. The model showed decent quality in all regions including the binding site according to QMEAN analysis (**Table S2**, **Figure S5**).

Network visualization of the docking poses

The network projection of the docking poses of **8–12** was generated to visualize the global pose space, where a connection between the two nodes (or poses) indicates a root mean square deviation (rmsd) of ≤ 0.75Å (**Figure S16A**). The network can be interactively explored to see how different poses coalesce into clusters trends (or don't) when viewed in the context of pose similarity on the basis of rmsd. The connections between the nodes and the size of nodes inform our understanding of the binding mode by helping us to identify which unique poses of **8–12**

are involved in the common binding mode (CBM). The network shows one large cluster 1 enclosed within the black boundary and other moderate to small clusters 2-10 consisting of poses of at least four out of the five ligands docked. The residual poses can be observed in the form of small clusters inside and around the periphery of the plot. Docking poses of **8–12** considered for the elucidation of a CBM were identified within cluster 1 (cyan circle) and exhibited shortest-path distance among themselves (**Figure S16A**). The clustering of interactive pharmacophore models generated from the poses of 1 (**Figure S16B**) revealed clusters of hydrogen bond donors (HBDs), hydrogen bond acceptors (HBAs) and hydrophobic features (**Figure S16C**) indicating that the majority of poses showed overlapping features that were developed as a result of common interaction partners in the binding site.

Binding free energy calculations

The free energy of binding (ΔG_{bind}) of the complexes obtained from the MD simulation was calculated by using the Molecular Mechanics–Poisson Boltzmann Surface Area (MM-PBSA) approach [7–9]. The results indicated that **9–12** possessed significantly high negative ΔG_{bind} as compared to **8** (**Table S3**, **Figure S21**). Based on the binding energy calculations, the estimated sensitivity of LAT1 to ligands may be expressed in the order **11** > **9** ~ **10** > **12** > **8**, which is qualitatively reliable with *in vivo* data of the NMs. Nevertheless, **8** was poorly predicted by MM-PBSA, though it is equipotent to **9** and ~ 9 times more potent than **10**. This deviation between the predicted and experimental value may be ascribed to the shortcomings of MM-PBSA in contrast to more precise methods of ΔG calculations, such as thermodynamic integration (TI) and free energy perturbation (FEP). The van der Waals (ΔG_{vdw}), electrostatic interactions (ΔG_{elect}) and non-polar solvation energy ($\Delta G_{non\text{-}polar}$) contributed negatively, while polar solvation energy (ΔG_{polar}) added positively to the total free binding energy of the ligands. The $\mathbf{r^2}$ between ΔG_{vdw} and ΔG_{bind} is 0.82, and $\mathbf{r^2}$ between ΔG_{elect} and ΔG_{bind} is 0.64. In terms of negative contribution, ΔG_{vdw} gives more significant contribution than ΔG_{elect} for all ligands except **8** suggesting significant hydrophobic interactions of the side chain. The lack of extended side chain in **8** may explain the low ΔG_{vdw} as compared to the NMs, and thus a smaller ΔG_{bind}. Moreover, in **8** and **12**, the contribution from the electrostatic and van der Waals energy was compensated mainly by the high polar solvation free energy resulting in reduced ΔG_{bind}. Overall, ΔG_{elect} and ΔG_{vdw} seems to be dominant forces contributing to the stability of complexes **8–12**. To identify the critical molecular determinants involved in the binding, per-residue energy contribution was computed. The binding of the ligands was mostly influenced

favorably by residues I139, I140, I147, V148, F252, W257, V339 and W405 *via* van der Waals interactions, while residues T62, I63, G65, S66, G67, F252, and S338 contributed *via* electrostatic interactions (**Figure S22**).

References

1. Gao, X. *et al.* Mechanism of substrate recognition and transport by an amino acid antiporter. *Nature* **463,** 828–832 (2010).
2. Shaffer, P. L., Goehring, A., Shankaranarayanan, A. & Gouaux, E. Structure and mechanism of a Na+-independent amino acid transporter. *Science* **325,** 1010–1014 (2009).
3. Fotiadis, D., Kanai, Y. & Palacín, M. The SLC3 and SLC7 families of amino acid transporters. *Mol. Aspects Med.* **34,** 139–158 (2013).
4. Laskowski, R. A., Moss, D. S. & Thornton, J. M. Main-chain bond lengths and bond angles in protein structures. *J. Mol. Biol.* **231,** 1049–1067 (1993).
5. Benkert, P., Tosatto, S. C. E. & Schomburg, D. QMEAN: A comprehensive scoring function for model quality assessment. *Proteins* **71,** 261–277 (2008).
6. Zhu, K. *et al.* Antibody structure determination using a combination of homology modeling, energy-based refinement, and loop prediction. *Proteins* **82,** 1646–1655 (2014).
7. Kumari, R., Kumar, R., Open Source Drug Discovery Consortium & Lynn, A. g_mmpbsa--a GROMACS tool for high-throughput MM-PBSA calculations. *J. Chem. Inf. Model.* **54,** 1951–1962 (2014).
8. Lee, M. R., Duan, Y. & Kollman, P. A. Use of MM-PB/SA in estimating the free energies of proteins: application to native, intermediates, and unfolded villin headpiece. *Proteins* **39,** 309–316 (2000).
9. Konecny, R., Baker, N. A. & McCammon, J. A. iAPBS: a programming interface to Adaptive Poisson-Boltzmann Solver (APBS). *Comput. Sci. Discov.* **5,** (2012).
10. Clamp, M., Cuff, J., Searle, S. M. & Barton, G. J. The Jalview Java alignment editor. *Bioinforma. Oxf. Engl.* **20,** 426–427 (2004).

11. Lomize, M. A., Pogozheva, I. D., Joo, H., Mosberg, H. I. & Lomize, A. L. OPM database and PPM web server: resources for positioning of proteins in membranes. *Nucleic Acids Res.* **40,** D370-376 (2012).

12. Deléage, G., Clerc, F. F., Roux, B. & Gautheron, D. C. ANTHEPROT: a package for protein sequence analysis using a microcomputer. *Comput. Appl. Biosci. CABIOS* **4,** 351–356 (1988).

13. Fruchterman TMJ, Reingold EM (1991) Graph drawing by force-directed placement. Softw Pract Exper 21:1129–1164.

	Human	Mouse	Rabbit	Dog
**TM2**	A85	S86	S81	A63
**TM3**	K132	K133	K128	R110
**TM4**	E169	E170	E165	S147
**TM8**	I326	I331	V322	I304
**TM9**	V374<br>V382<br>L386<br>K391	V379<br>I387<br>M391<br>R396	V370<br>A378<br>L382<br>R387	M352<br>I360<br>L364<br>R369
**TM10**	V396<br>I413<br>I416	I401<br>I418<br>M421	V392<br>I409<br>M412	V374<br>A391<br>L394
**TM12**	V456<br>T463<br>F474	M461<br>A468<br>F479	V452<br>T459<br>F470	V434<br>T441<br>L452

Table S1. The amino acid residue differences in the TMs of LAT1 of mouse, rabbit, and dog with respect to the human sequence. The corresponding substitutions are indicated in red.

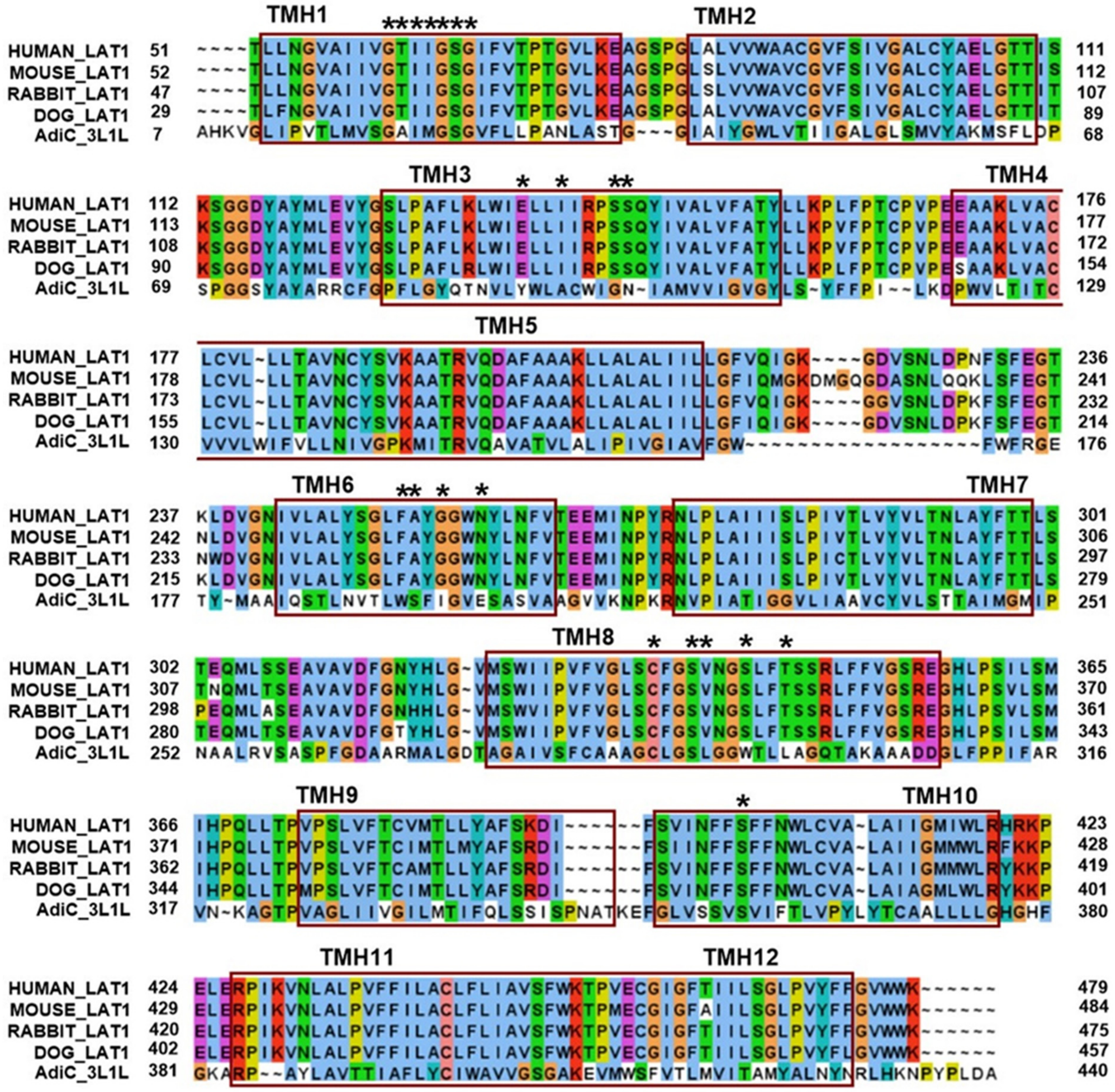

Figure S1. LAT1–AdiC alignment as visualized using Jalview [10]. The residues are colored according to their type using the Clustalx color scheme. The TMs are indicated as brown boxes. The TMs of AdiC were defined using the PPM server [11]. The residues of LAT1 involved in direct interactions with the docking poses of **8–12** are highlighted with a black asterisk.

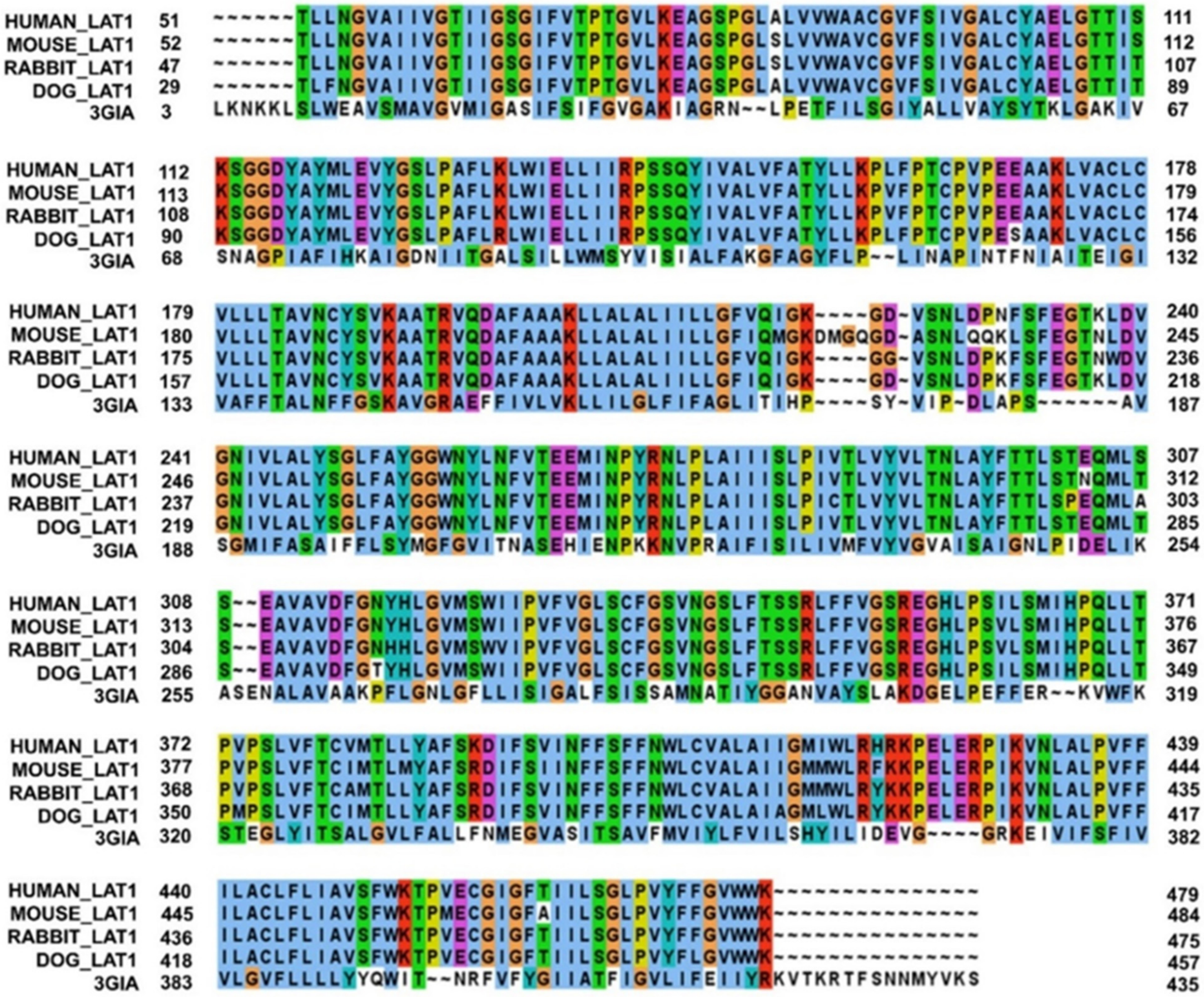

Figure S2. LAT1–ApcT alignment.

Template	AdiC (PDB ID: 3L1L)
Sequence Identity	20.33%
Residues in most favored regions	88.4%
Residues in additionally allowed regions	9.5%
Generously allowed regions	1.6%
Residues in disallowed regions	0.5%
Normalized DOPE score	-0.39
G factor	0.40
Q mean	0.47
Z Score	-3.49
Errat (Overall quality factor)	93.57

Table S2. Assessment of LAT1 model built on the AdiC structure (PDB ID: 3L1L).

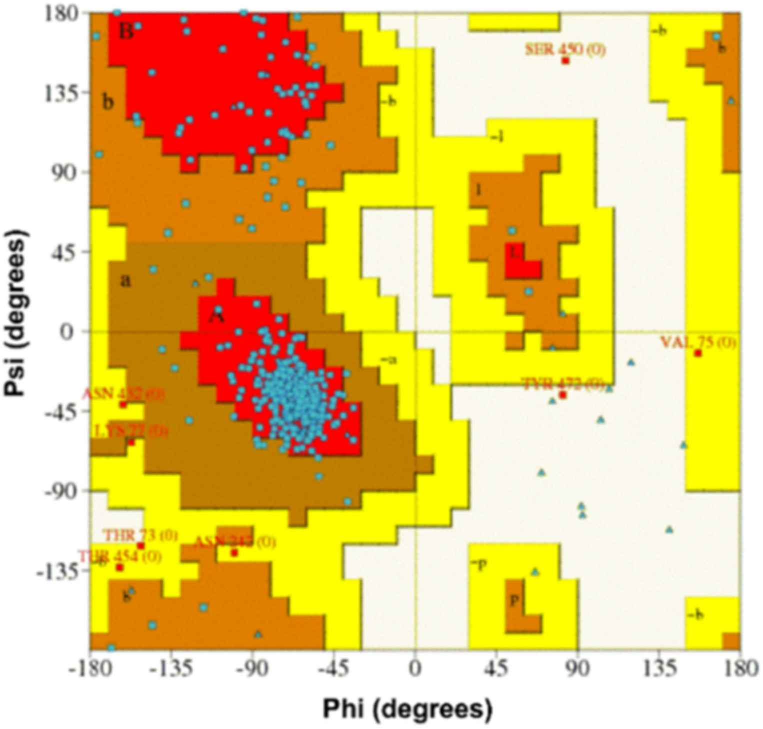

Figure S3. The Ramachandran plot of LAT1 model based on the AdiC structure.

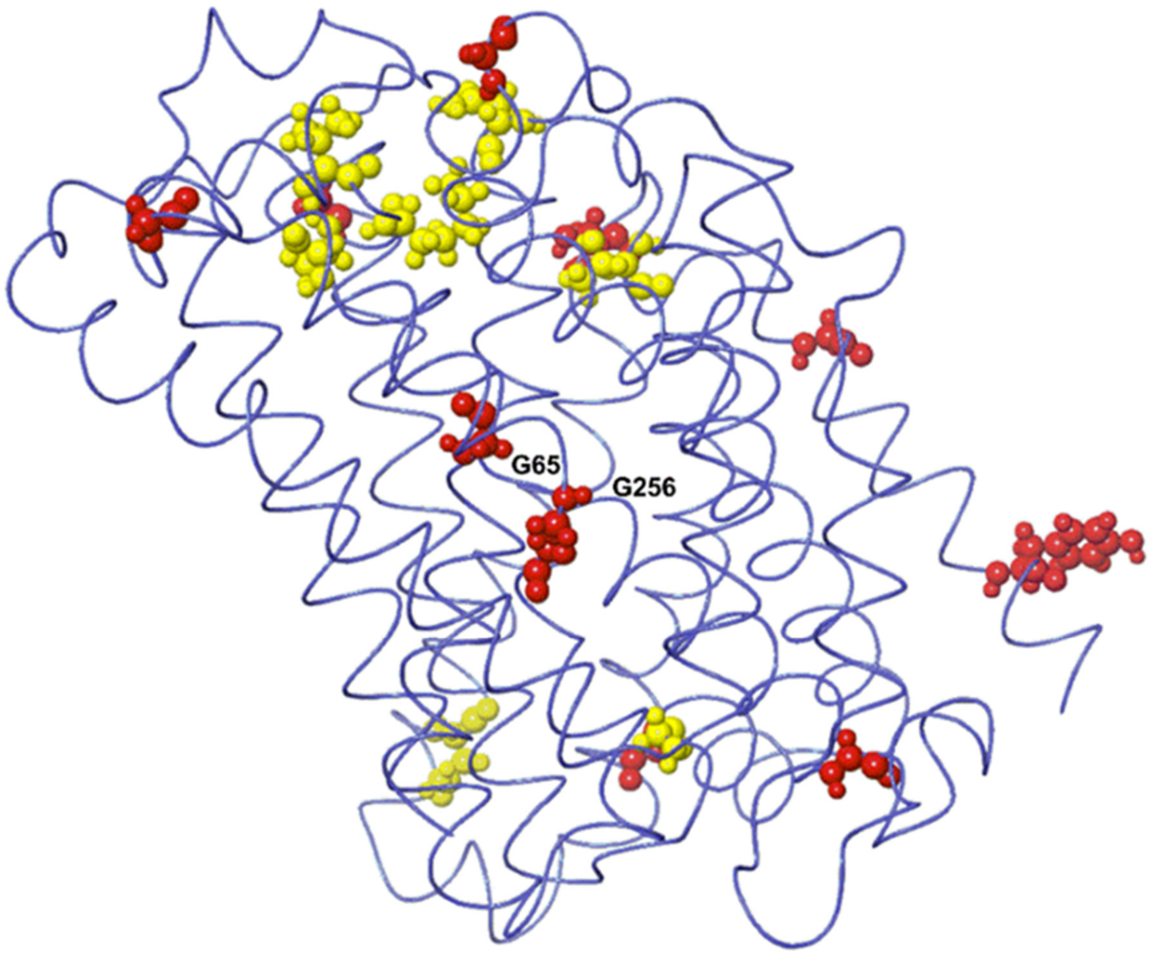

Figure S4. Outliers defined by PROCHECK analysis. Residues in generously allowed regions are shown in yellow and in disallowed areas as red.

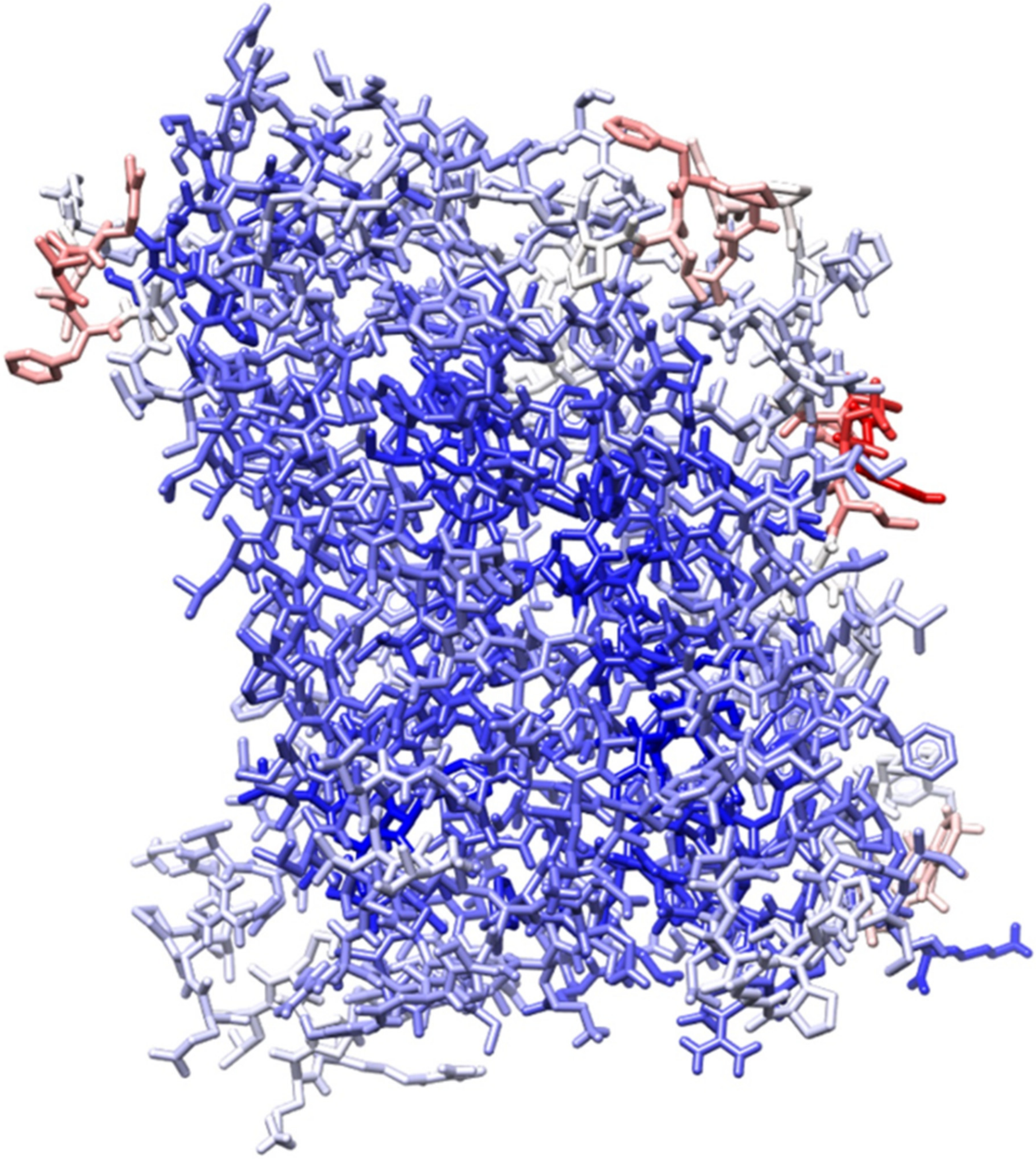

Figure S5. The QMEAN analysis of LAT1 model (blue: high quality and more reliable regions, red: low-quality regions and potentially unreliable regions; estimated error above 3.5Å).

Ligand	ΔG_{vdw}	ΔG_{elect}	ΔG_{polar}	$\Delta G_{non\text{-}polar}$	ΔG_{bind}
**8**	-26.26 ± 2.73	-34.47 ± 3.12	59.47 ± 2.79	-3.04 ± 0.14	-4.30 ± 2.84
**9**	-43.92 ± 2.83	-19.44 ± 3.89	50.73 ± 3.82	-4.53 ± 0.18	-17.16 ± 3.20
**10**	-43.71 ± 2.98	-24.26 ± 2.68	55.46 ± 2.85	-4.56 ± 0.18	-17.07 ± 2.95
**11**	-40.68 ± 2.74	-21.83 ± 2.85	47.61 ± 3.13	-4.27 ± 0.17	-19.17 ± 3.08
**12**	-42.14 ± 3.61	-34.70 ± 3.62	67.84 ± 3.03	-4.27 ± 0.19	-13.27 ± 3.21

Table S3. Average MM-PBSA free energies of 8–12 calculated from the 20 ns MD simulations. ΔG_{bind} (free energy of binding), ΔG_{elect} (electrostatic energy), ΔG_{vdw} (van der Waals energy), ΔG_{polar} (polar solvation free energy) and $\Delta G_{non\text{-}polar}$ (non-polar solvation free energy). All energies are in kcal mol^{-1}.

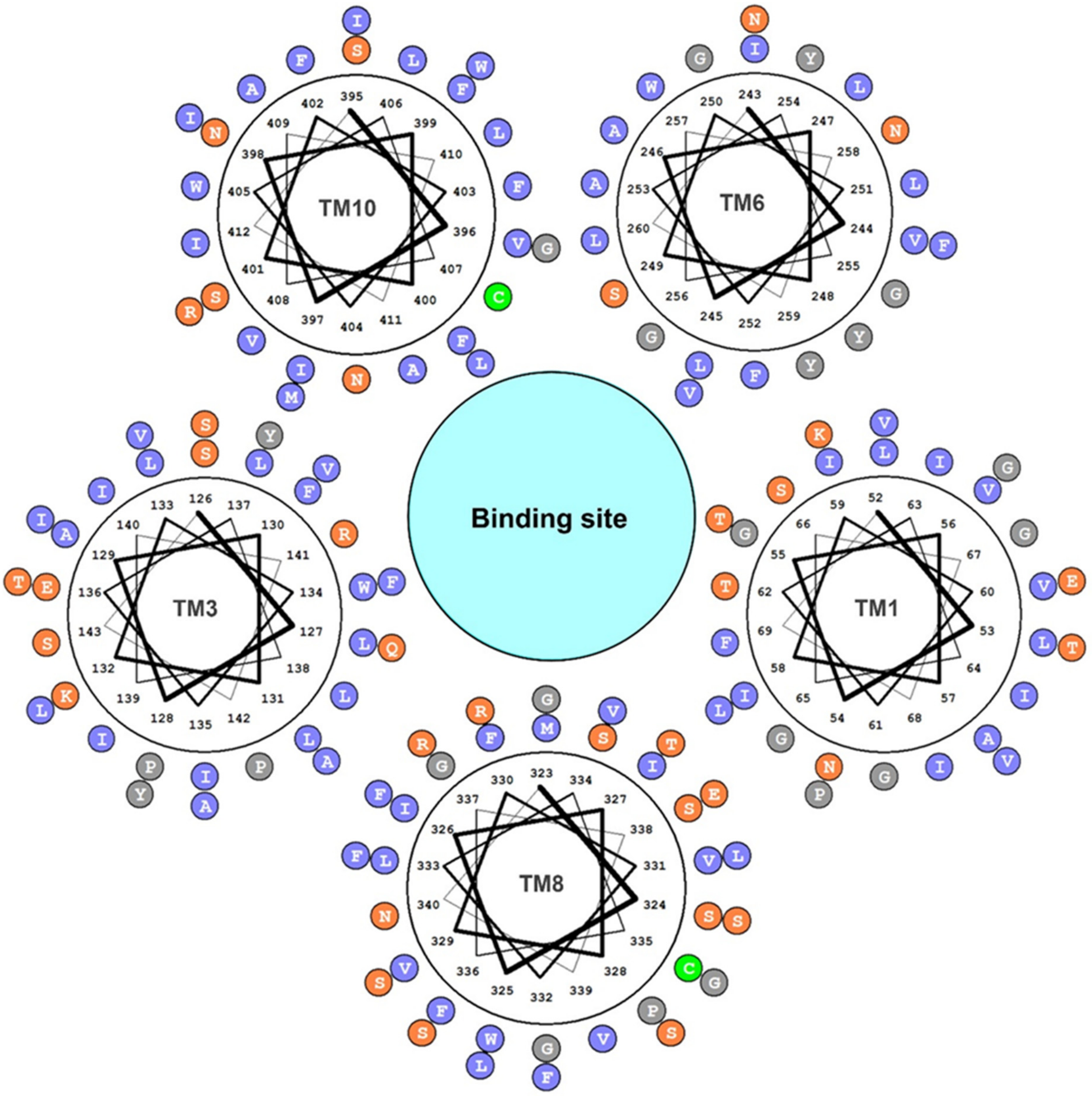

Figure S6. Helical projection diagram [12] of the TMs (1, 3, 6, 8, and 10) enclosing the binding site of LAT1. Hydrophobic residues are indicated in blue, hydrophilic residues in orange, cysteine residues in green and others in grey.

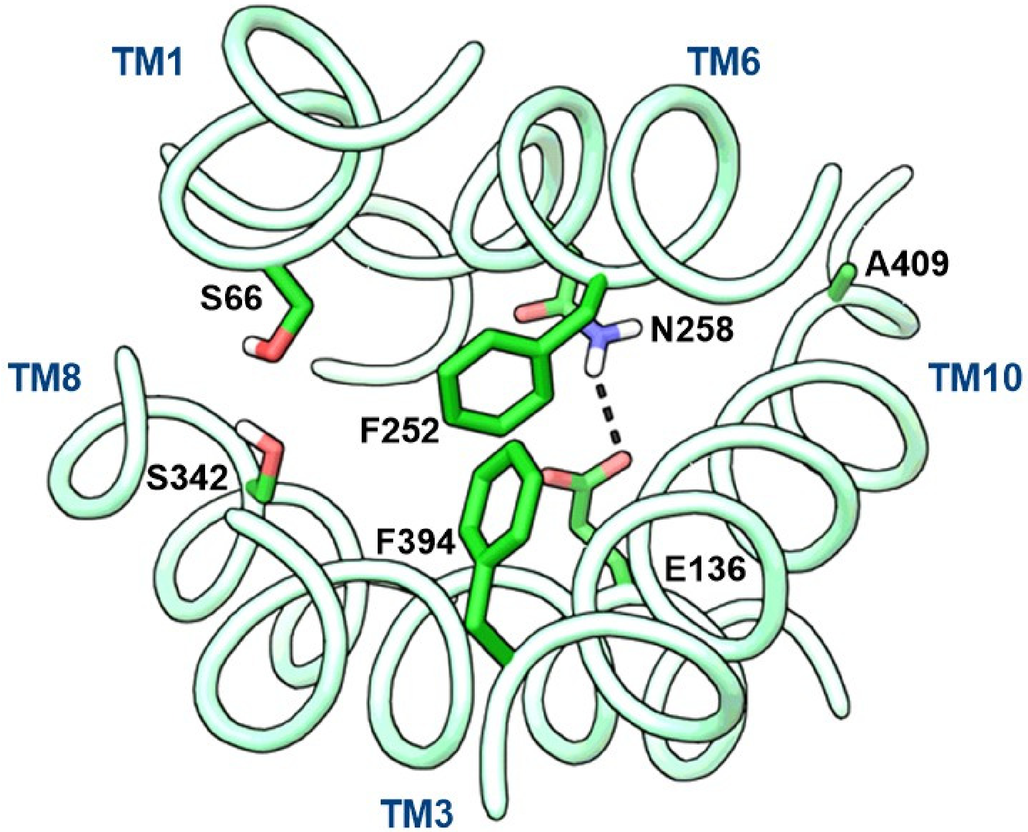

Figure S7. The putative gate residues of LAT1 (Doorway residue: F394, Proximal gate: S66 and F252, Middle gate: S342, Distal gate: N258, E136, and A409). The residues (green) are shown in stick representation. The hydrogen bond interaction between N258 and E136 possibly indicates a closed distal gate.

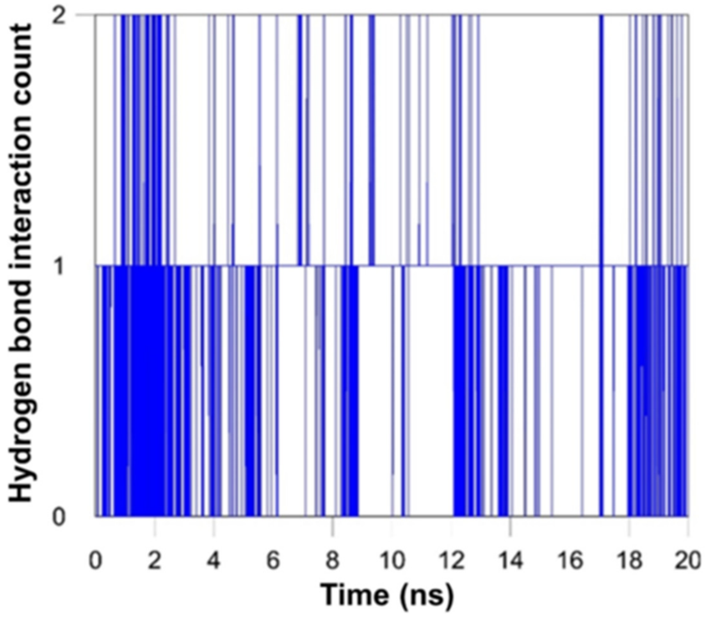

Figure S8. The plot of hydrogen bond interaction counts between N258 and E136 of LAT1 complexed with **11** as a function of simulation time.

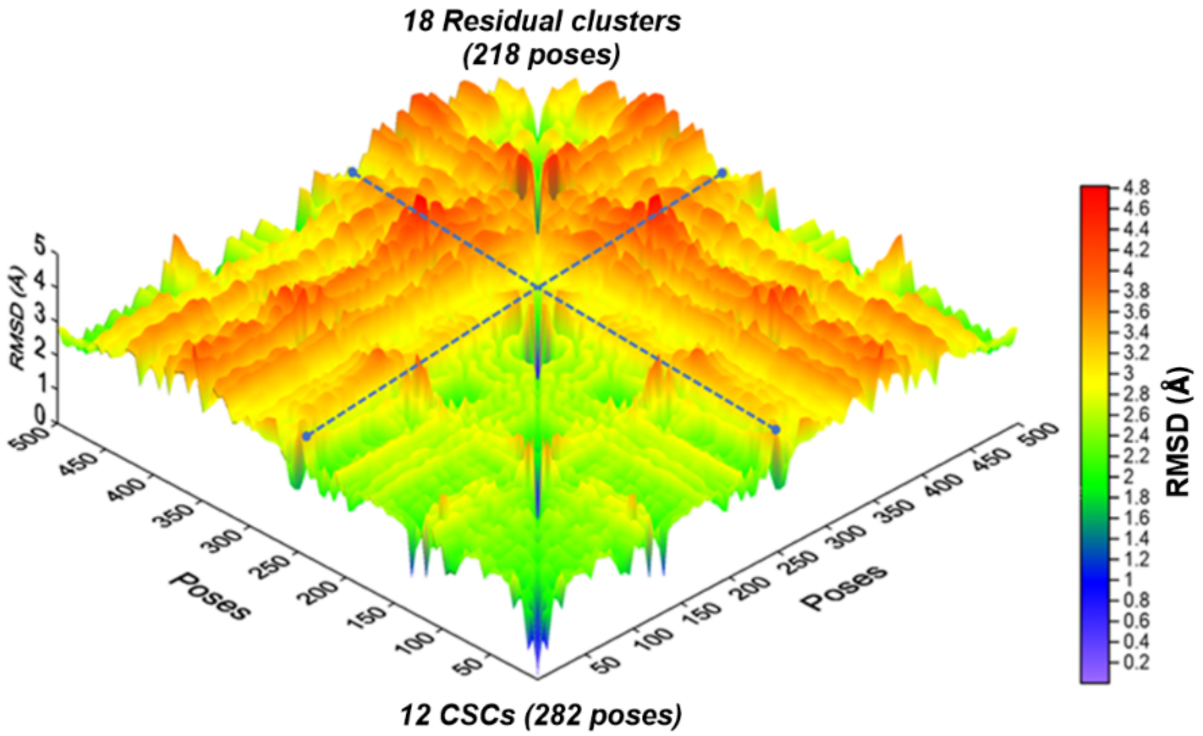

Figure S9. The surface landscape of 500 docking poses based on the rmsd matrix.

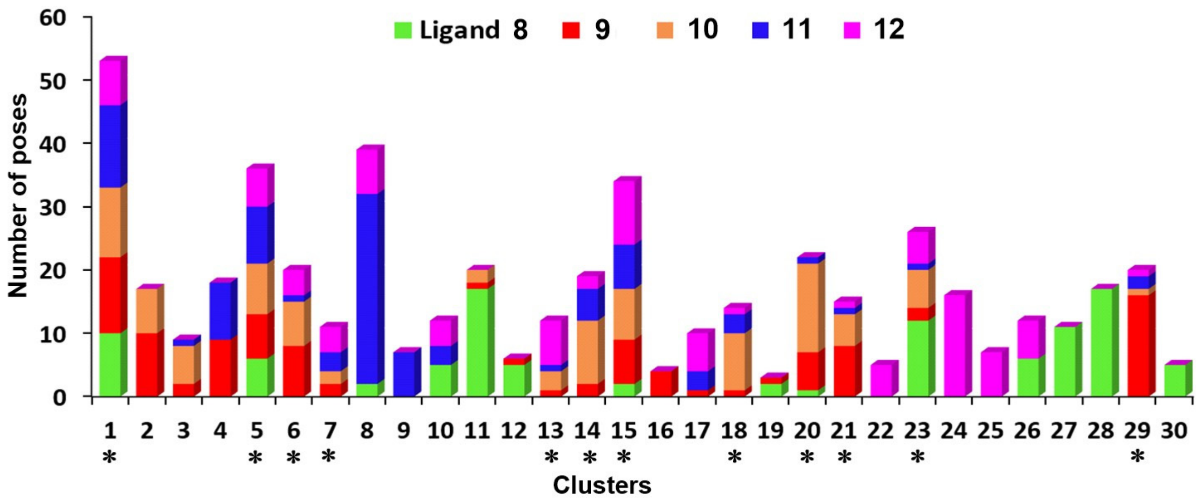

Figure S10. The cluster statistics of docking showing the distribution of poses of **8–12** in clusters 1–30. Clusters marked with an asterisk are common scaffold cluster (CSC), while the rest are residual clusters.

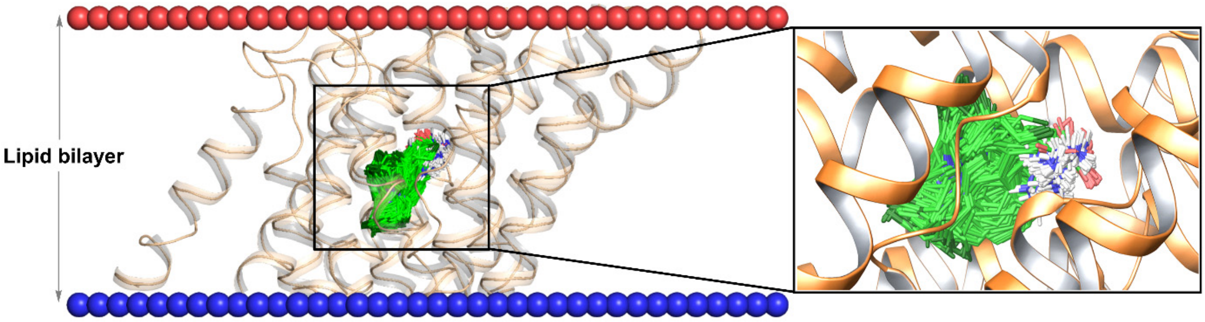

Figure S11. Distribution of 282 poses corresponding to 12 CSCs. The lipid bilayer of LAT1 model was defined using the PPM server [11]. It is observed from the distribution that the length of the poses is inclined with respect to the lipid bilayer. The α–amino and the α–carboxyl groups are pointing towards the periplasmic side, while the tetrahydronaphthalene moieties are occupying the center of the binding site. The NM side chains are directed intra- and extra-cellular.

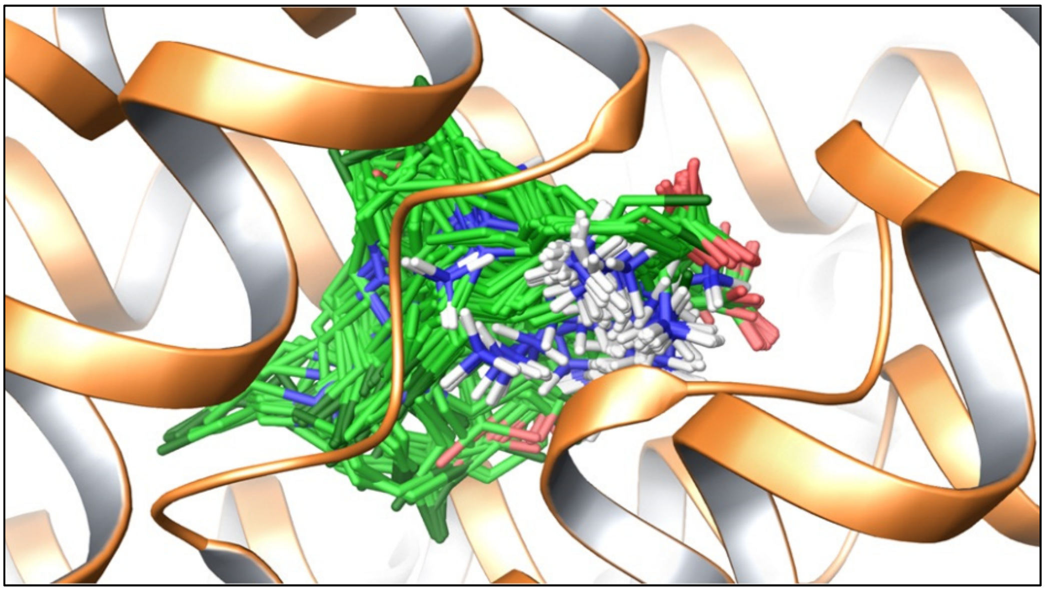

Figure S12. Distribution of 218 poses corresponding to 18 residual clusters. It is observed from the distribution that the α–amino, the α–carboxyl and the NM moieties are spread over a wide area in the binding site. In some poses, the α–amino and the α–carboxyl groups are directed intra-cellular.

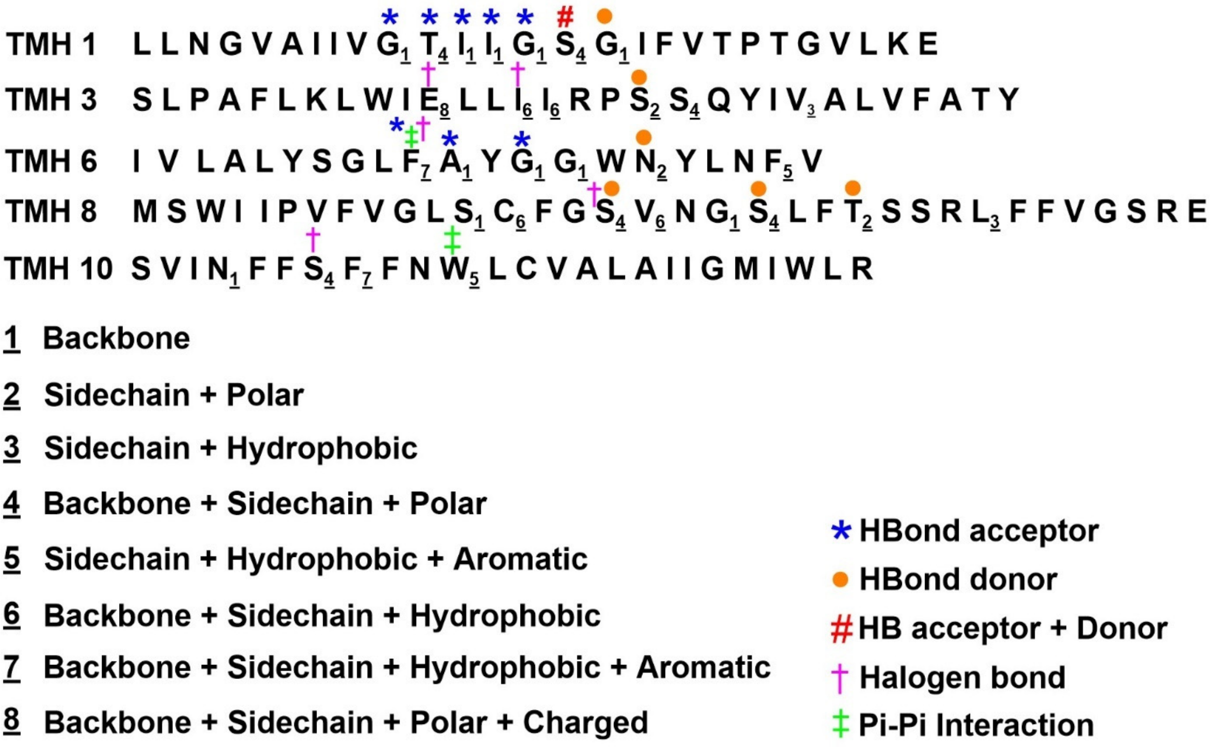

Figure S13. SIFt represented on schematic helices aligned according to the relative amino acid positions and their interaction type in 500 docking poses.

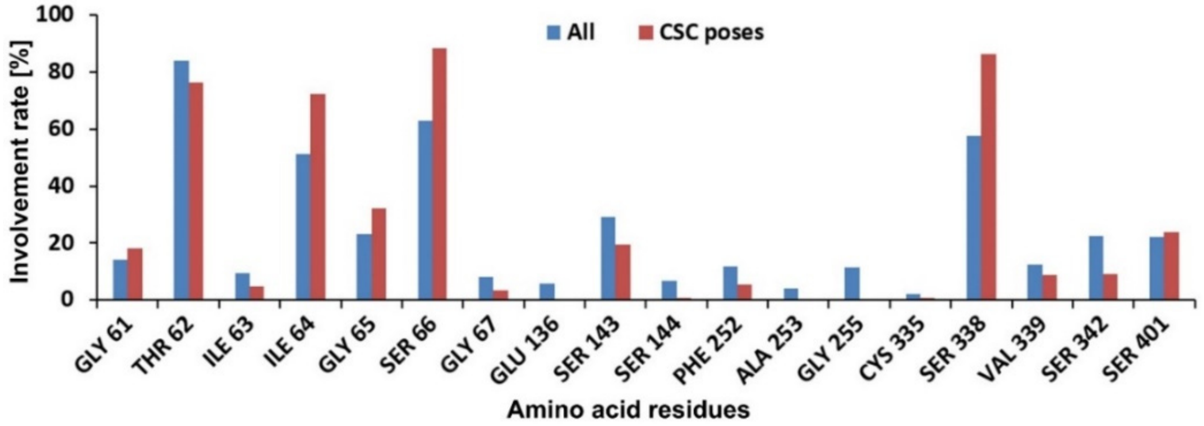

Figure S14. Structural interaction fingerprint (SIFt) showing the involvement rate of residues in hydrogen bond interactions (excluding hydrogen bond interactions of chlorine) in all 500 poses (blue) and 282 CSC poses (red).

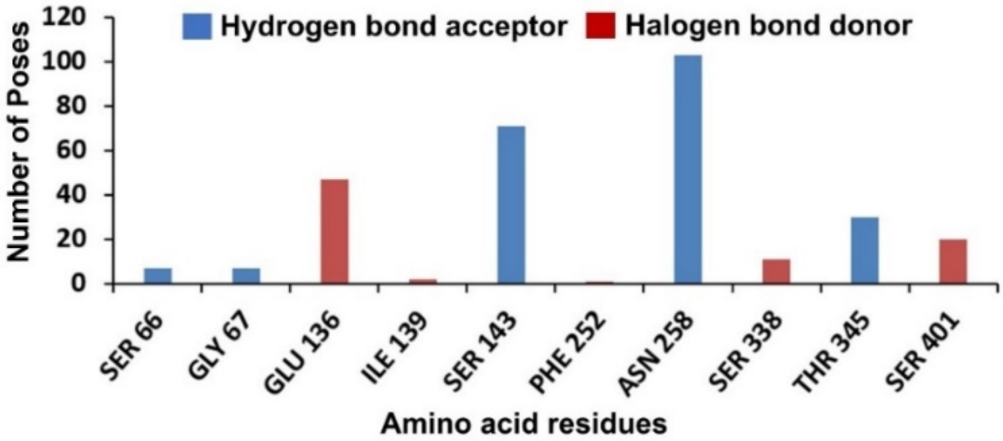

Figure S15. SIFt showing the polar interactions of the chlorine atom with the residues in 500 docking poses. The blue vertical bars indicate the poses where chlorine is acting as a hydrogen bond acceptor from the residue, and the red vertical bars indicate poses where chlorine is donating a halogen bond to the residue.

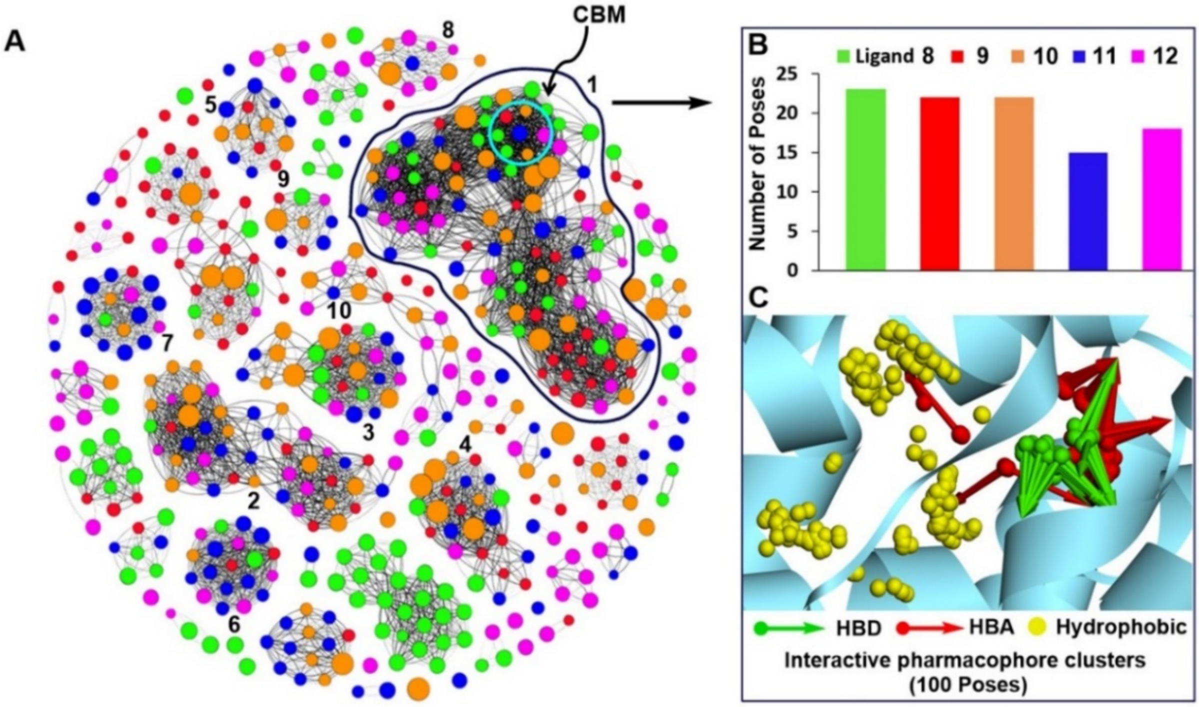

Figure S16. A, The network map of 500 docking poses as visualized using the Fruchterman–Reingold algorithm [13]. In the graph, each node corresponds to a distinct pose of **8** (green), **9** (red), **10** (orange), **11** (blue), and **12** (purple). A link between the two nodes (or poses) corresponds to a rmsd of ≤ 0.75Å. The size of each node is proportional to the docking score, i.e., a more prominent node indicates a high negative docking score, while a small node indicates a low negative docking score. The densely populated cluster 1 is indicated with a black boundary. Docking poses within the cyan circle of cluster 1 were considered for the elucidation of CBM. **B**, distribution of the poses of **8–12** corresponding to cluster 1. **C**, interactive pharmacophore clusters generated from the poses of 1.

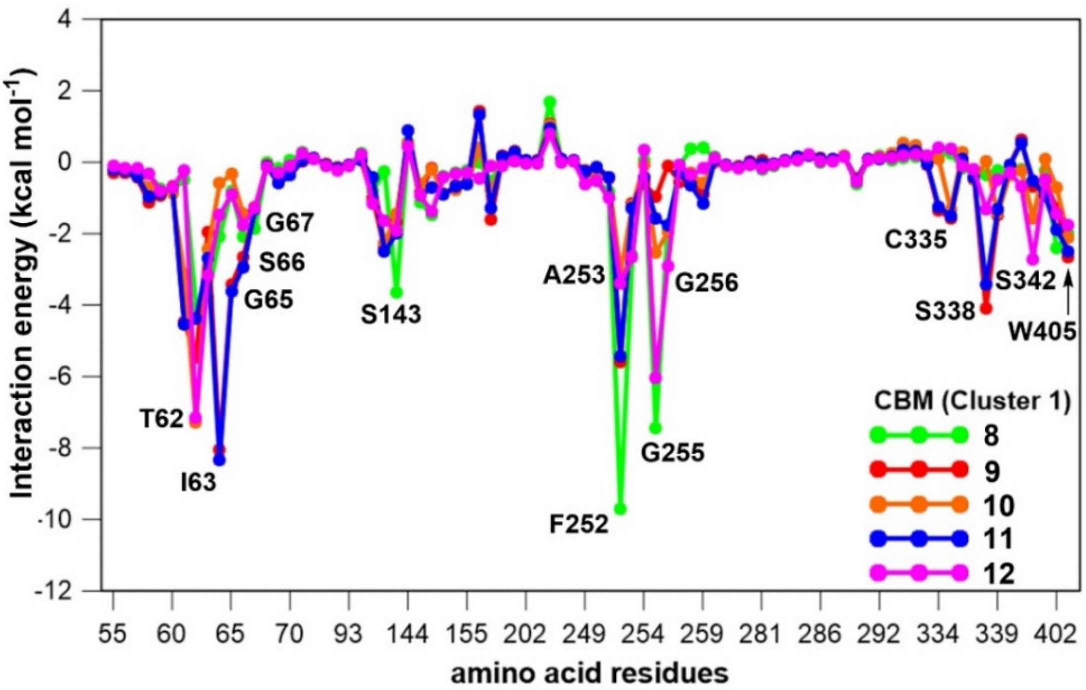

Figure S17. Per-residue energy contribution to the docking pose of **8–12**.

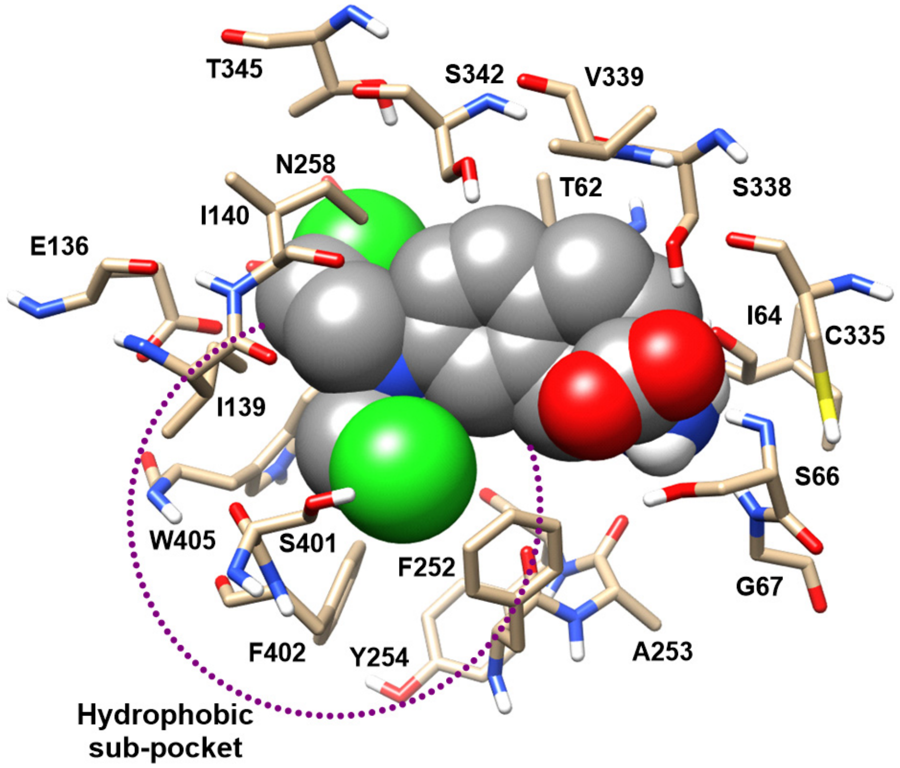

Figure S18. The last snapshot of the molecular dynamics (MD) simulation of complex **11** depicting the Side chain Binding Site (SBS) or hydrophobic sub-pocket (HSP). The ligand and the interacting residues are shown in space-filling and stick style, respectively. A violet dashed circle indicates the HSP comprising residues I139, F252, Y254, F402, and W405.

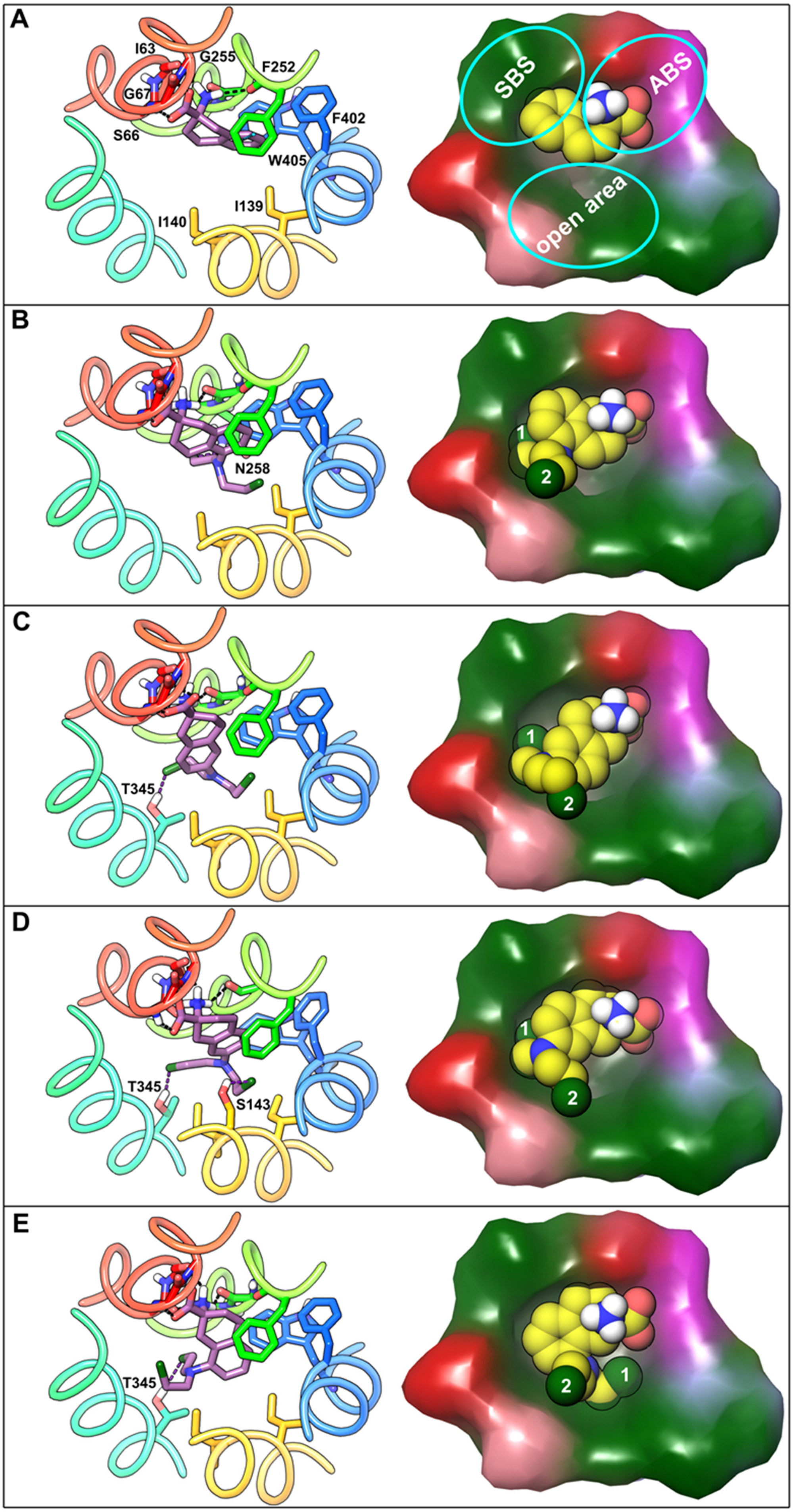
A
I63
G255
F252
G67
S66
F402
W405
I139
I140
SBS
ABS
open area
B
N258
1
2
C
T345
1
2
D
T345
S143
1
2
E
T345
2
1

Figure S19. The predicted binding mode of **8** (**A**), **9** (**B**), **10** (**C**), **11** (**D**), and **12** (**E**) in LAT1. The ligands are shown in stick representation, and their carbon atoms are colored purple. The interacting residues are shown in stick representation, and their carbon atoms are colored according to the color of the corresponding TM helix. The figure adjacent to the interactive binding mode shows occupancy of the ligand atoms in the binding site. The ligands are depicted in space-filling style, and their carbon atoms are colored yellow. The chloroethyl moieties are numbered 1 and 2. The binding site surface is colored according to the residue type, i.e., the green areas are hydrophobic, while the red and purple regions are hydrophilic.

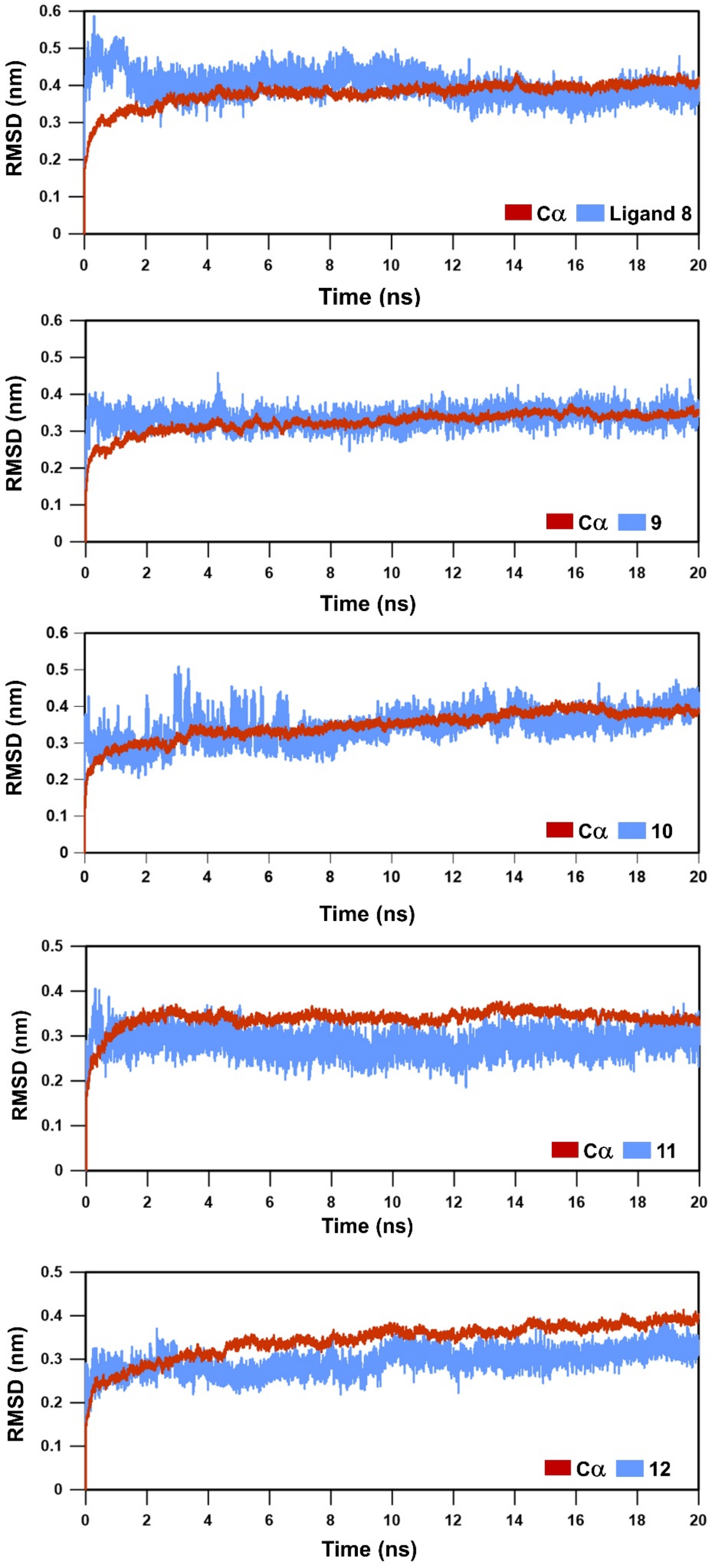

Figure S20. The rmsd plots as a function of simulation time.

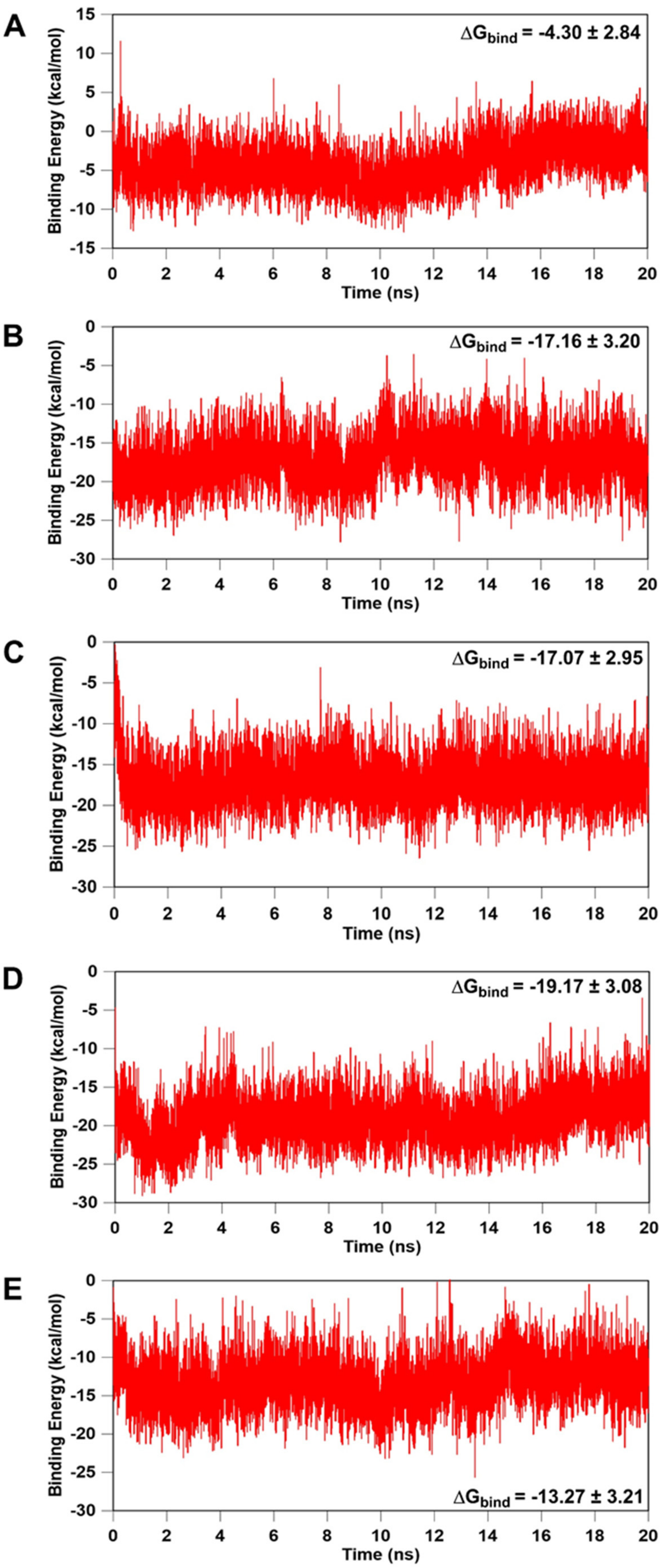

Figure S21. The plot of binding energy as a function of simulation time of complex **8** (**A**), **9** (**B**), **10** (**C**), **11** (**D**) and **12** (**E**). ΔG_{bind} represents the average binding energy of the ligand.

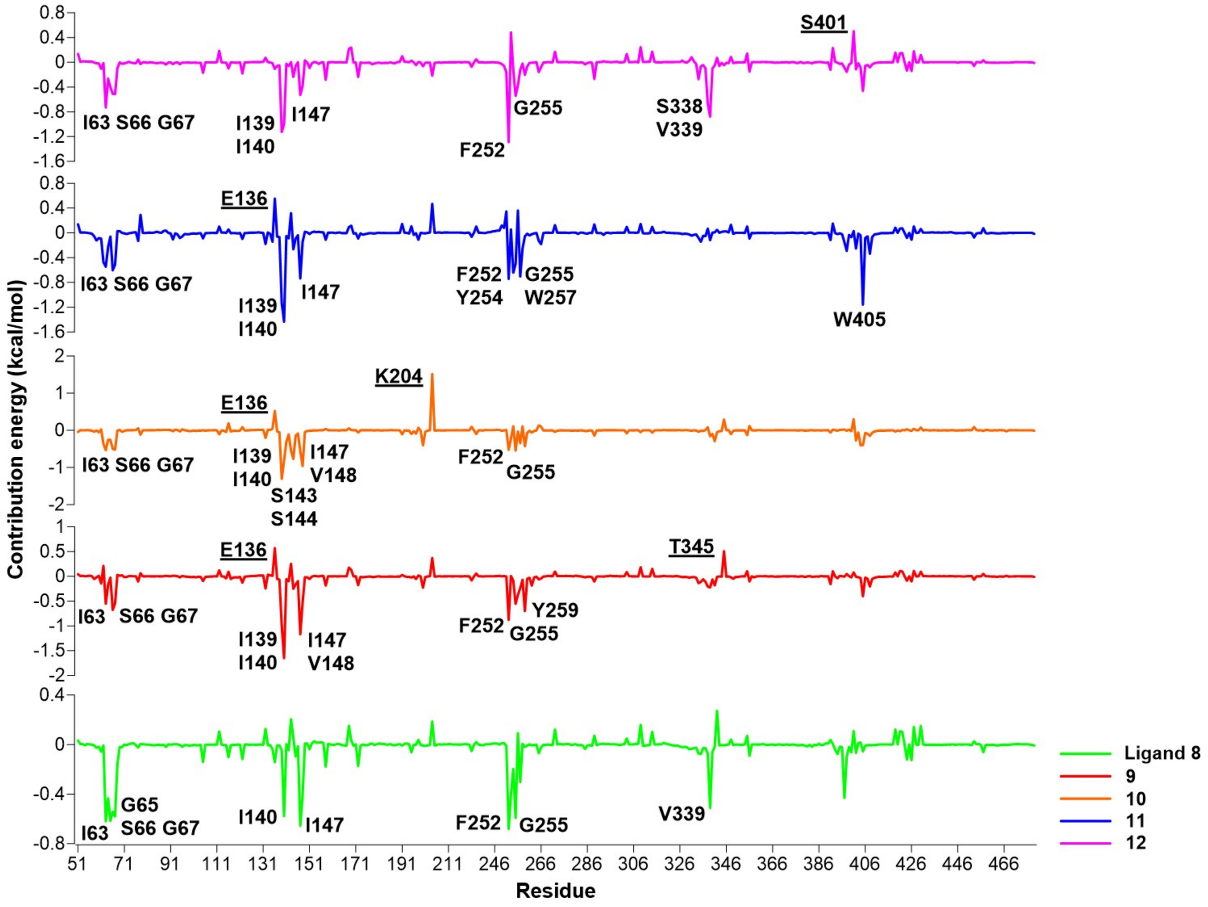

Figure S22. The average per-residue contribution to the binding energy (ΔG_{bind}) of each complex. The residues that contributed ≤ -0.5 kcal mol^{-1} in the binding are indicated, whereas the residues that impaired the binding with ≥ 0.5 kcal mol^{-1} are underlined.

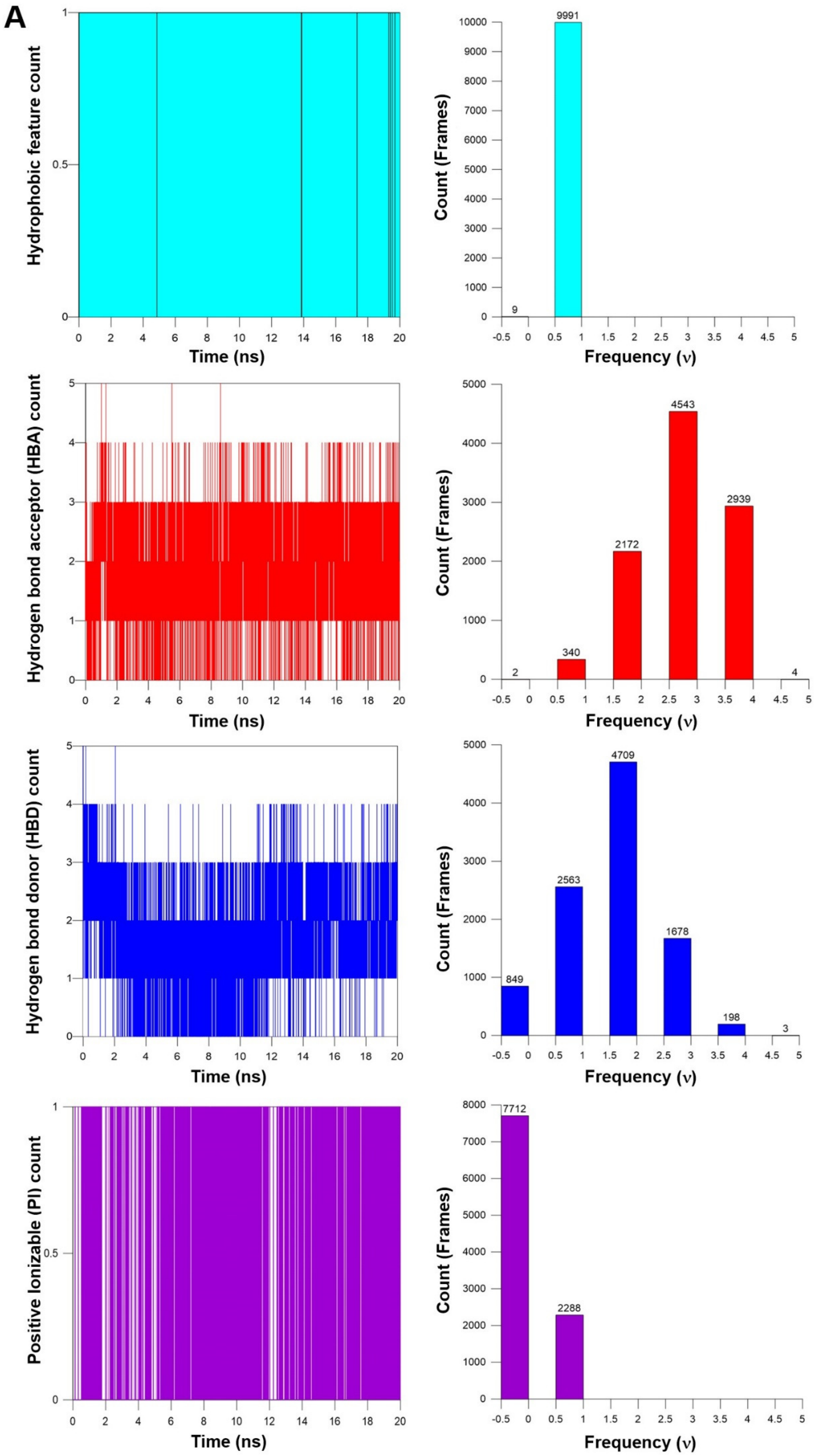
A
Hydrophobic feature count
Time (ns)
Count (Frames)
Frequency (ν)
9991
9
Hydrogen bond acceptor (HBA) count
Time (ns)
Count (Frames)
Frequency (ν)
2
340
2172
4543
2939
4
Hydrogen bond donor (HBD) count
Time (ns)
Count (Frames)
Frequency (ν)
849
2563
4709
1678
198
3
Positive Ionizable (PI) count
Time (ns)
Count (Frames)
Frequency (ν)
7712
2288

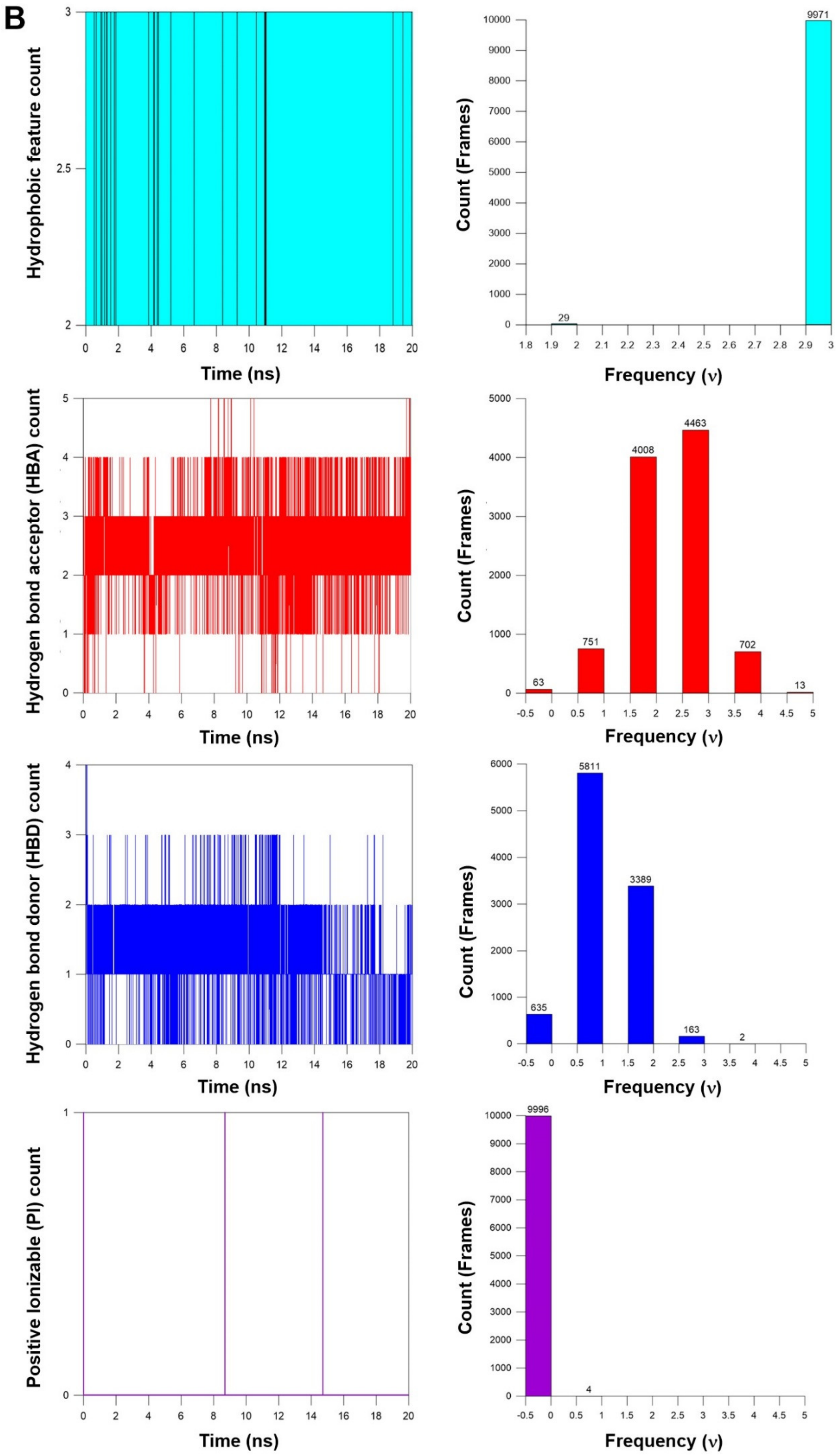
B
Hydrophobic feature count
Time (ns)
Count (Frames)
Frequency (ν)
9971
29
Hydrogen bond acceptor (HBA) count
Time (ns)
Count (Frames)
Frequency (ν)
63
751
4008
4463
702
13
Hydrogen bond donor (HBD) count
Time (ns)
Count (Frames)
Frequency (ν)
635
5811
3389
163
2
Positive Ionizable (PI) count
Time (ns)
Count (Frames)
Frequency (ν)
9996
4

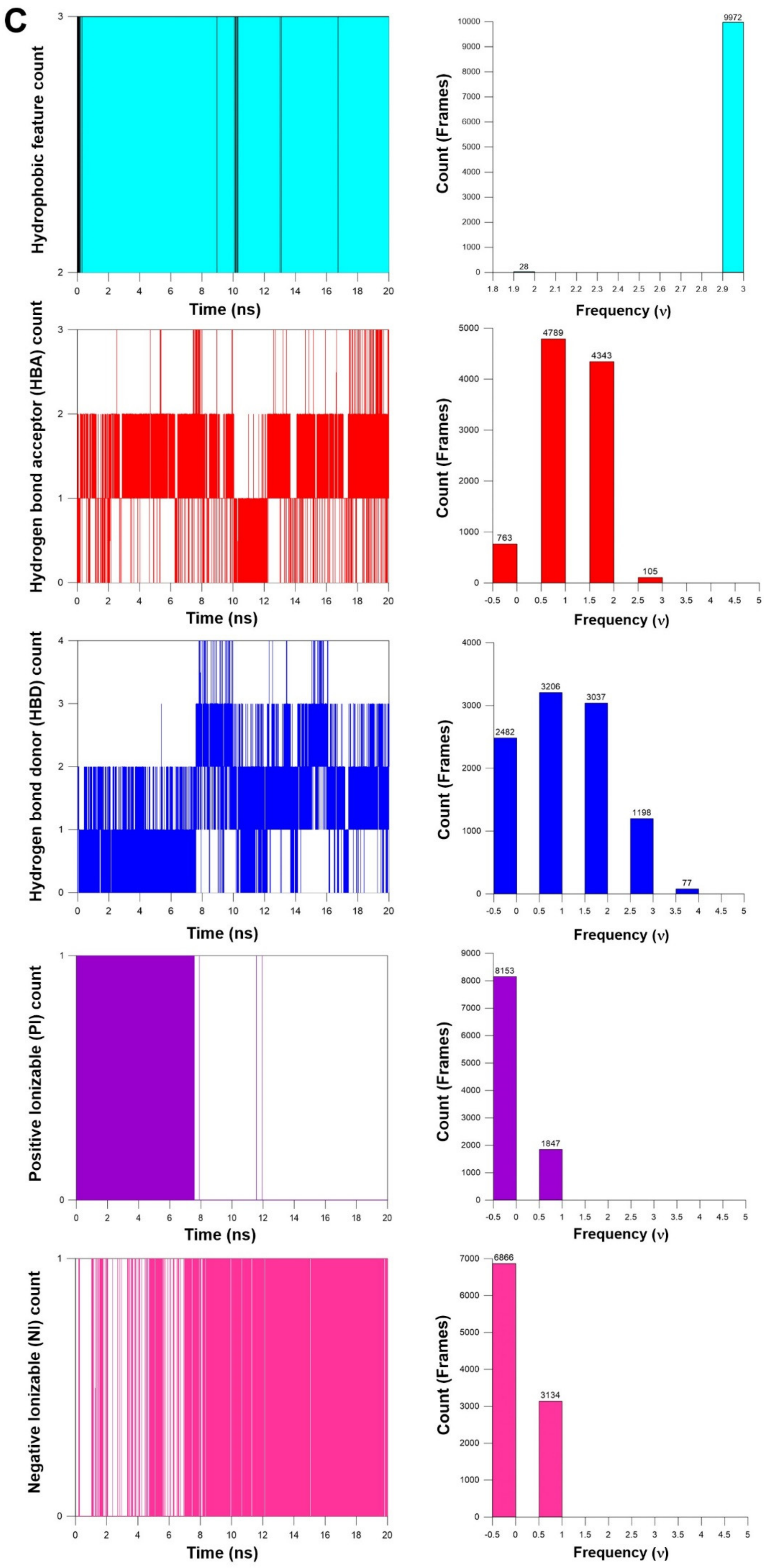
C
Hydrophobic feature count
Time (ns)
Count (Frames)
Frequency (ν)
9972
28
Hydrogen bond acceptor (HBA) count
4789
4343
763
105
Hydrogen bond donor (HBD) count
2482
3206
3037
1198
77
Positive Ionizable (PI) count
8153
1847
Negative Ionizable (NI) count
6866
3134

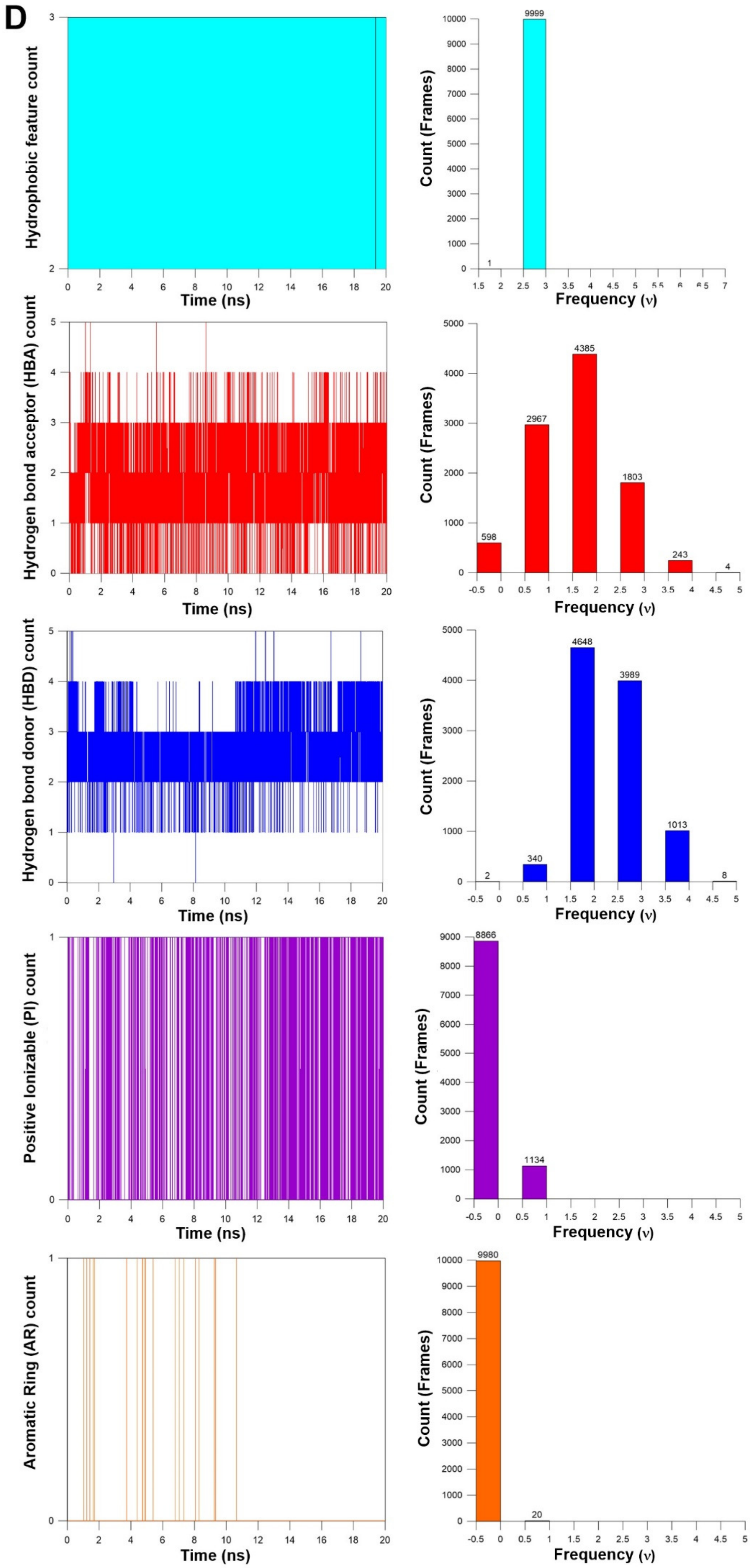
D
Hydrophobic feature count
Time (ns)
Count (Frames)
Frequency (ν)
9999
1
Hydrogen bond acceptor (HBA) count
598
2967
4385
1803
243
4
Hydrogen bond donor (HBD) count
2
340
4648
3989
1013
8
Positive Ionizable (PI) count
8866
1134
Aromatic Ring (AR) count
9980
20

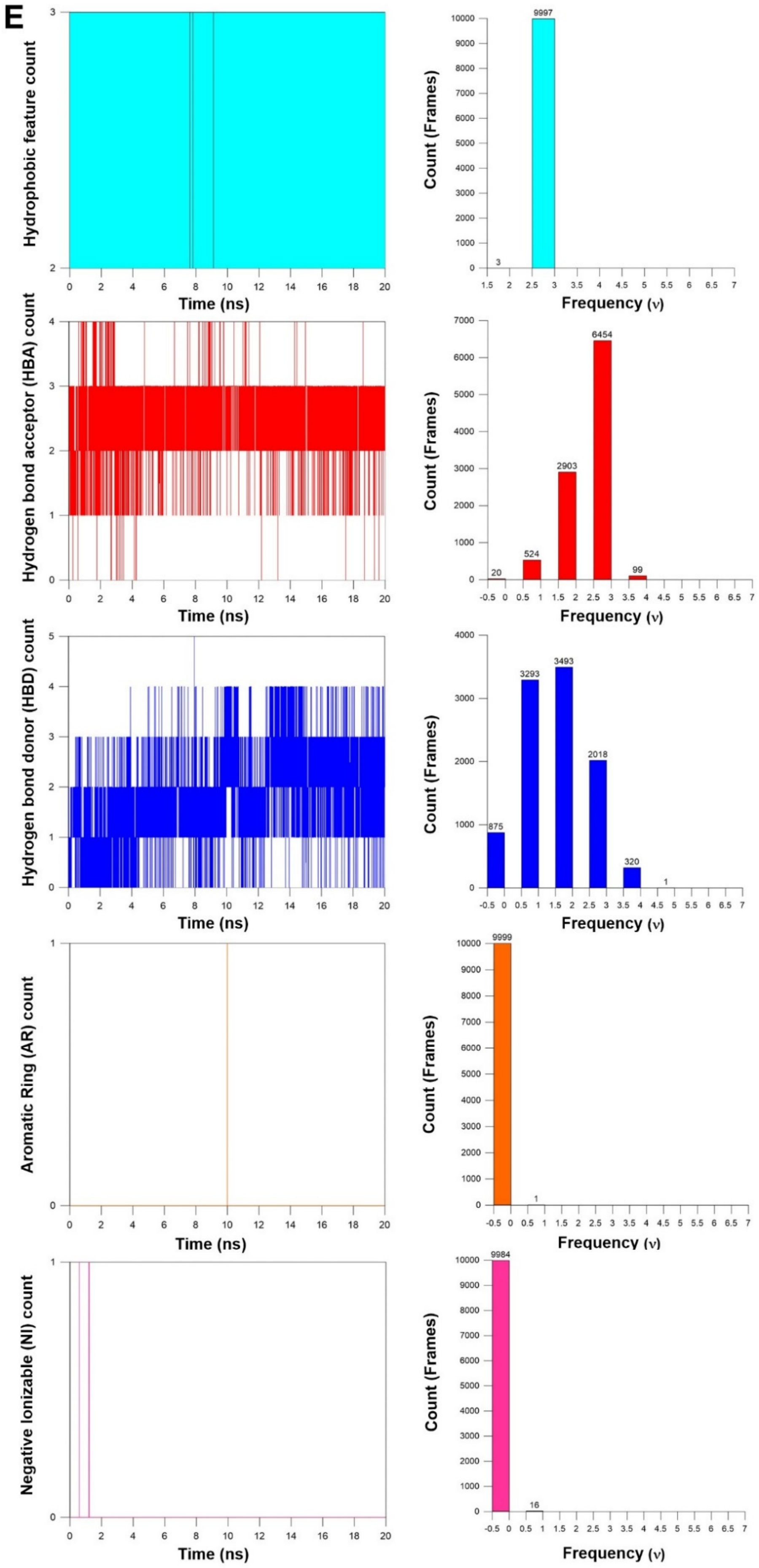
E
Hydrophobic feature count
Time (ns)
Count (Frames)
9997
3
Frequency (ν)
Hydrogen bond acceptor (HBA) count
Time (ns)
Count (Frames)
6454
2903
524
20
99
Frequency (ν)
Hydrogen bond donor (HBD) count
Time (ns)
Count (Frames)
3293
3493
2018
875
320
1
Frequency (ν)
Aromatic Ring (AR) count
Time (ns)
Count (Frames)
9999
1
Frequency (ν)
Negative Ionizable (NI) count
Time (ns)
Count (Frames)
9984
16
Frequency (ν)

Figure S23. Plots showing the evolution of different pharmacophoric features as a function of simulation time of **8** (**A**), **9** (**B**), **10** (**C**), **11** (**D**) and **12** (**E**) bound to LAT1.

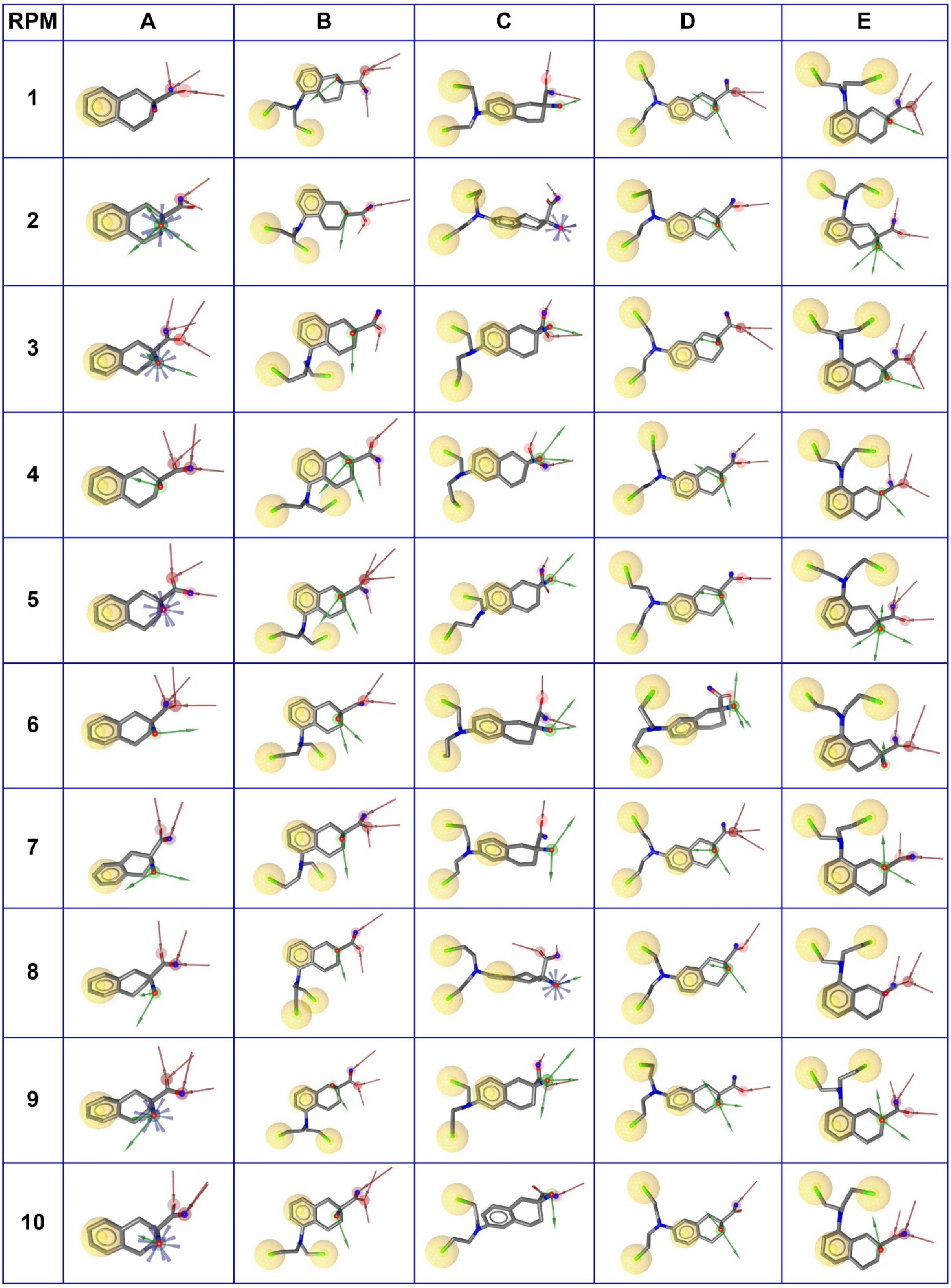

Figure S24. The representative pharmacophore models (RPMs) of 10 most populated clusters of dynamic pharmacophores of **8** (**A**), **9** (**B**), **10** (**C**), **11** (**D**), and **12** (**E**).

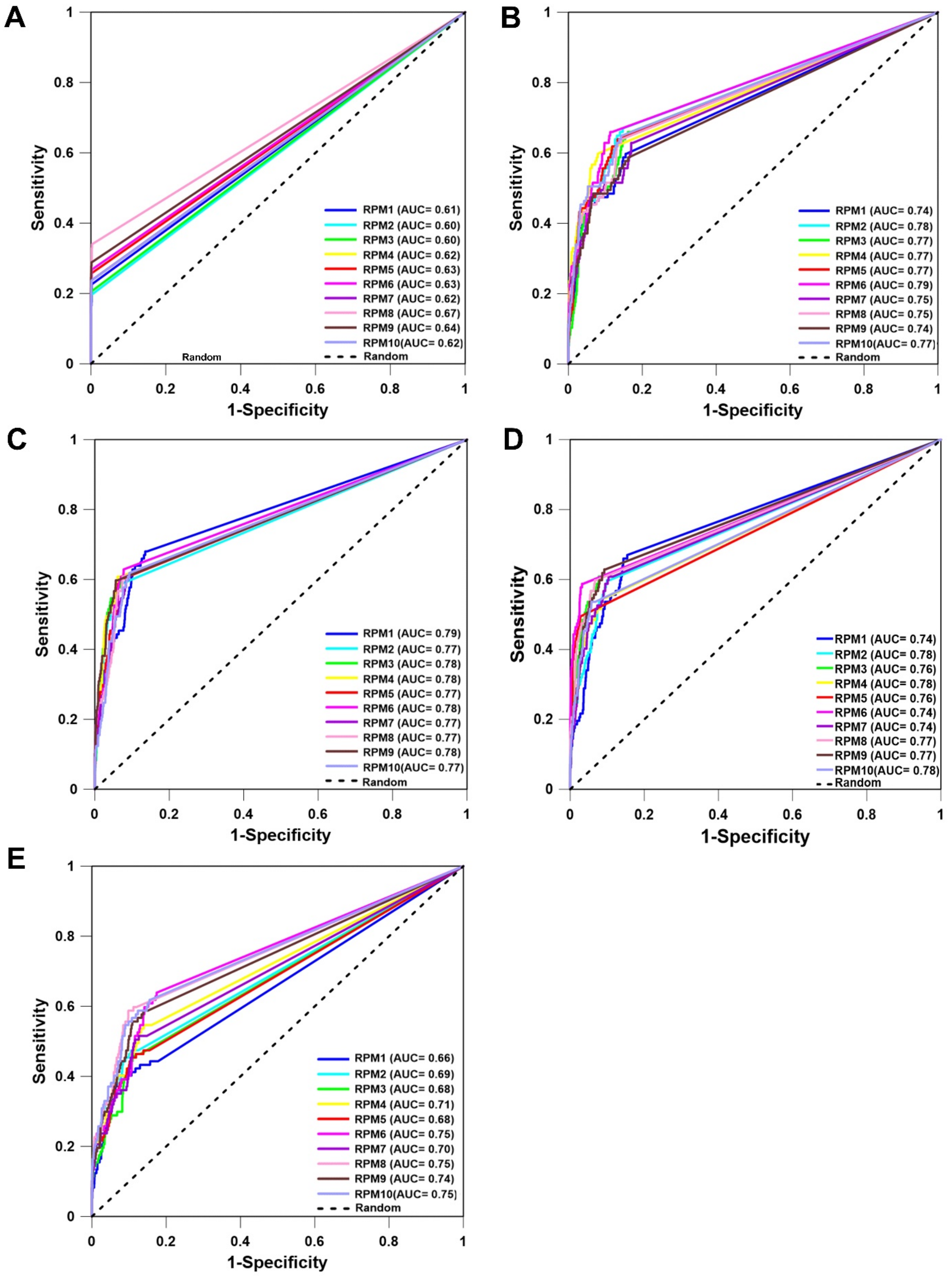

Figure S25. The receiver operating characteristic (ROC) curve validation of the RPMs of 10 most populated clusters of dynamic pharmacophores of **8** (**A**), **9** (**B**), **10** (**C**), **11** (**D**) and **12** (**E**). The true positive rate (Sensitivity) is shown on the Y-axis, and the false positive rate (1-Specificity) is on the X-axis.

Cpd.	RPM	ROC	AUC	RIE	EF 1%	EF 2%	EF 5%	EF 10%	EF 20%
**8**	1	0.605	0.612	4.31	23	11	4.5	2.3	1.1
	2	0.591	0.598	3.78	20	9.8	3.9	2	0.98
	3	0.595	0.602	4.15	22	11	4.3	2.2	1.1
	4	0.611	0.618	4.53	24	12	4.7	2.4	1.2
	5	0.622	0.628	4.91	26	13	5.2	2.6	1.3
	6	0.627	0.633	5.07	27	13	5.4	2.7	1.3
	7	0.611	0.618	4.71	25	12	4.9	2.5	1.2
	8	0.663	0.669	6.34	34	17	6.8	3.4	1.7
	9	0.638	0.644	5.47	29	14	5.8	2.9	1.4
	10	0.611	0.618	4.52	24	12	4.7	2.4	1.2
**9**	1	0.736	0.74	6.29	15	10	8.2	4.7	3
	2	0.777	0.781	7.19	25	14	8.2	5.2	3.4
	3	0.762	0.766	6.1	10	8.2	8.2	4.9	3.3
	4	0.766	0.77	7.92	25	16	8.9	6	3
	5	0.766	0.77	7	14	13	8.9	5.4	3.2
	6	0.781	0.785	7.31	24	14	7.4	5.8	3.3
	7	0.746	0.75	6.58	22	12	7.4	4.9	3.1
	8	0.746	0.75	7.02	21	14	8.7	4.7	3.2
	9	0.730	0.735	6.42	19	12	7.4	4.8	2.9
	10	0.770	0.774	7.28	18	14	9.3	5.2	3.2
**10**	1	0.784	0.788	6.92	20	12	8	5.6	3.4
	2	0.763	0.767	7.46	15	12	9.7	5.9	2.9
	3	0.773	0.777	7.75	14	13	11	6.1	3
	4	0.775	0.779	7.97	19	14	10	6.1	3
	5	0.766	0.77	7.2	19	14	9.1	6	3
	6	0.778	0.782	7.23	20	13	7.6	6.3	3.1
	7	0.767	0.771	6.86	15	12	7.8	6.1	3.1
	8	0.767	0.771	7.04	22	12	7.4	6.1	3
	9	0.772	0.776	8.11	23	16	10	6	3
	10	0.766	0.77	6.59	12	9.8	8.4	6.1	3.1
**11**	1	0.733	0.737	5.85	14	9.3	6.8	5.2	3.4
	2	0.778	0.782	6.65	19	12	7.4	5.6	3
	3	0.758	0.762	8.06	23	16	10	6.1	3
	4	0.771	0.775	7.01	20	14	8.7	5.5	2.7
	5	0.753	0.757	8.05	25	20	9.9	4.9	2.5
	**6**	**0.735**	**0.739**	**9.41**	**35**	**22**	**12**	**5.9**	**2.9**
	7	0.738	0.742	6.9	16	12	8	5.7	3
	8	0.770	0.774	8.12	21	18	9.9	6	3
	9	0.768	0.772	7.55	20	12	9.5	6.2	3.1
	10	0.776	0.78	7.16	19	13	9.1	5.4	2.7
**12**	1	0.649	0.655	4.75	12	7.7	5.8	4	2.2
	2	0.684	0.689	5.62	18	9.8	6.6	4.5	2.4
	3	0.671	0.677	4.72	14	8.2	5.4	4	2.3
	4	0.706	0.711	5.68	20	11	6.2	4.2	2.7
	5	0.671	0.677	5.2	18	9.8	5.4	4	2.4
	6	0.744	0.748	5.68	20	12	5.8	3.9	3.2
	7	0.693	0.698	5.23	15	10	5.8	3.7	2.6
	8	0.748	0.752	6.37	22	12	6.8	5.6	3
	9	0.731	0.736	5.84	16	9.8	6.2	4.7	2.9
	10	0.748	0.752	6.42	20	12	7.4	5.5	3.1

Table S4. The performance metrics in the validation of the RPMs of 10 most populated clusters of dynamic pharmacophores of **8–12**.

Cpd.	Pharmacophore fit score	GoldScore	ChemPLP Score	Consensus score	Rank
**13**	47.16	44.092	55.296	2.407	18
**14**	47.36	57.462	83.935	2.715	2
**15**	46.95	48.946	59.047	2.365	24
**16**	47.30	49.573	64.883	2.543	7
**17**	47.42	54.544	61.855	2.590	4
**18**	47.77	12.136	58.808	2.551	6
**19**	47.16	35.507	71.039	2.473	9
**20**	47.24	19.651	69.415	2.436	13
**21**	47.21	46.581	52.957	2.421	14
**22**	47.00	49.761	64.243	2.420	15
**23**	46.37	47.776	65.138	2.169	57
**24**	46.54	30.320	63.625	2.161	61
**25**	46.33	46.032	67.491	2.161	62
**26**	46.15	53.853	61.094	2.079	85
**27**	46.49	48.416	67.036	2.230	46
**28**	46.32	49.358	57.450	2.107	77
**29**	46.88	47.373	62.115	2.351	27
**30**	46.63	44.046	68.114	2.276	41
**31**	46.52	52.367	59.661	2.211	50
**32**	46.49	48.416	67.036	2.230	36
**33**	46.63	44.046	68.114	2.276	41
**34**	45.49	41.986	58.335	1.755	128
**35**	46.33	50.264	66.138	2.168	59
**36**	46.26	41.761	62.653	2.087	83
**37**	45.48	33.423	37.754	1.591	131
**38**	46.00	56.208	65.327	2.054	94
**39**	46.68	43.655	72.470	2.322	32
**40**	46.14	35.799	54.006	1.963	112
**41**	45.64	30.245	71.869	1.856	124
**42**	46.53	40.617	59.520	2.170	56

Table S5. Pharmacophore fit score, docking scores, consensus score, and rank of the hit compounds selected for experimental testing; Total number of compounds screened (pharmacophore-based): 1148189 → Total number of compounds docked to LAT1: 1202 → Consensus scoring: 1202 → top-ranked 200 out of 1202 compounds were considered for the final selection of compounds for experimental testing

Cpd.	% Residual Activity	Standard Deviation (n = 3)	MACCS			Radial ECFP			FP2		
DMSO	100	0	1	2	11	1	2	11	1	2	11
1	6.1	0.3									
13	74.23	16.4	0.27	0.5	0.28	0.02	0.05	0.04	0.06	0.31	0.16
14	95.87	16.7	0.13	0.33	0.19	0.01	0.07	0.04	0.03	0.17	0.10
15	92.21	1.8	0.27	0.46	0.46	0.03	0.07	0.06	0.09	0.19	0.17
16	95.1	5.1	0.22	0.44	0.26	0.01	0.06	0.03	0.09	0.21	0.21
17	94.75	10.7	0.28	0.3	0.42	0.01	0.03	0.05	0.10	0.15	0.20
18	90.14	7.9	0.27	0.62	0.4	0.02	0.08	0.06	0.08	0.20	0.15
19	93.06	2	0.32	0.44	0.47	0.03	0.07	0.05	0.06	0.24	0.18
20	90.07	7.1	0.25	0.5	0.34	0.01	0.08	0.05	0.11	0.23	0.20
21	85.14	7.8	0.29	0.37	0.39	0.03	0.06	0.04	0.10	0.18	0.16
22	93.36	4.7	0.26	0.42	0.4	0.01	0.07	0.05	0.08	0.24	0.20
23	96.22	5.5	0.26	0.44	0.37	0.03	0.06	0.07	0.13	0.25	0.30
24	100.72	10.6	0.23	0.55	0.42	0.02	0.07	0.07	0.11	0.30	0.22
25	94.12	3.3	0.37	0.5	0.37	0.09	0.08	0.04	0.16	0.23	0.21
26	89.37	11.8	0.25	0.44	0.37	0.06	0.06	0.04	0.14	0.30	0.34
27	67.96	1.97	0.35	0.47	0.39	0.05	0.06	0.07	0.20	0.29	0.31
28	12.93	5.35	0.38	0.58	0.46	0.04	0.06	0.11	0.14	0.20	0.23
29	98.42	11	0.32	0.37	0.5	0.02	0.05	0.05	0.15	0.14	0.22
30	94.08	8.1	0.26	0.41	0.44	0.04	0.06	0.05	0.13	0.22	0.22
31	91.83	16.6	0.27	0.56	0.42	0.02	0.08	0.05	0.09	0.26	0.24
32	53.11	4.67	0.75	0.52	0.68	0.04	0.06	0.12	0.45	0.23	0.45
33	17.86	1.94	0.53	0.48	0.53	0.08	0.13	0.05	0.19	0.16	0.25
34	77.12	3.64	0.37	0.51	0.37	0.09	0.14	0.04	0.14	0.24	0.23
35	11.78	0.11	0.44	0.56	0.46	0.04	0.09	0.1	0.21	0.34	0.35
36	0	0	0.56	0.47	0.6	0.11	0.14	0.07	0.32	0.27	0.44
37	65.31	10.53	0.47	0.54	0.45	0.09	0.16	0.04	0.21	0.31	0.40
38	13.17	10.48	0.38	0.68	0.51	0.08	0.2	0.06	0.19	0.42	0.36
39	11.76	2.99	0.53	0.42	0.53	0.05	0.06	0.11	0.17	0.18	0.27
40	11.34	2.78	0.44	0.55	0.46	0.08	0.14	0.05	0.19	0.23	0.31
41	15.21	10.2	0.5	0.43	0.51	0.1	0.17	0.08	0.26	0.27	0.40
42	7.83	0.4	0.5	0.71	0.44	0.07	0.17	0.04	0.15	0.44	0.36

Table S6. The percent residual activity of compounds measured at 100 μM concentration and Tanimoto coefficients, derived from substructure-based fingerprint (MACCS), circular fingerprint (ECFP) and path-based fingerprint (FP2), of the tested compounds to **1** (BCH), **2** (KYT-0353) and **11** (DL-2-NAM-7).

[] μM	Cpd. 28			Cpd. 42			Cpd. 36		
	Exp. 1	Exp. 2	Exp. 3	Exp. 1	Exp. 2	Exp. 3	Exp. 1	Exp. 2	Exp. 3
**0**	100.00	100.00	100.00	100.00	100.00	100.00	100.00	100.00	100.00
**0.1**	98.56	118.69	87.20	91.01	109.97	91.55	81.30	100.31	62.08
**0.2**	109.35	122.43	93.48	77.34	82.24	73.67	69.42	119.31	58.21
**0.5**	112.59	110.90	85.27	72.30	76.32	64.01	54.32	77.26	5.56
**1**	120.86	119.63	97.34	64.39	51.09	41.55	38.13	55.14	40.82
**2.5**	84.89	85.98	94.20	42.45	36.45	24.15	39.93	44.24	0.97
**5**	79.14	103.43	83.33	33.81	36.14	17.39	16.55	0.00	1.21
**10**	96.40	89.72	73.19	38.49	10.90	5.31	0.00	0.00	0.00
**25**	57.91	52.02	47.58	39.21	14.33	9.66	21.94	17.45	1.21
**50**	46.76	49.53	20.29	12.95	13.71	13.30	0.00	6.23	0.00
**100**	44.24	13.08	16.18	20.14	19.31	4.11	28.42	14.02	0.00

Mean			Standard Deviation		
Cpd. 28	Cpd. 42	Cpd. 36	Cpd. 28	Cpd. 42	Cpd. 36
100.00	100.00	100.00	0.00	0.00	0.00
101.48	97.51	81.23	15.95	10.80	19.12
108.42	77.75	82.32	14.50	4.30	32.53
102.92	70.88	45.71	15.31	6.28	36.62
112.61	52.34	44.70	13.24	11.47	9.14
88.36	34.35	28.38	5.09	9.32	23.84
88.63	29.11	5.92	12.98	10.22	9.22
86.44	18.24	0.00	11.95	17.76	0.00
52.51	21.07	13.53	5.18	15.88	10.91
38.86	13.32	2.08	16.14	0.38	3.60
24.50	14.52	14.15	17.17	9.03	14.21

Table S7. Dose-response analysis of compounds **28**, **42** and **36**.

Cpd.	Screening database	Vendor	Catalog number	Purity (%)	Purity data	Identity data
**13**	Chembridge	Chembridge	90864634	> 90%	LC-MS	^{1}H-NMR
**14**	Chembridge	Chembridge	17592342	> 90%	LC-MS	^{1}H-NMR
**15**	Chembridge	Chembridge	75729207	> 90%	LC-MS	^{1}H-NMR
**16**	Chembridge	Chembridge	69089454	> 90%	LC-MS	^{1}H-NMR
**17**	Chembridge	Chembridge	42920737	> 90%	LC-MS	^{1}H-NMR
**18**	Chembridge	Chembridge	59415756	> 90%	LC-MS	^{1}H-NMR
**19**	Chembridge	Chembridge	96309693	> 90%	LC-MS	^{1}H-NMR
**20**	Chembridge	Chembridge	93476697	> 90%	LC-MS	^{1}H-NMR
**21**	Chembridge	Chembridge	61429699	> 90%	LC-MS	^{1}H-NMR
**22**	Chembridge	Chembridge	56805360	> 90%	LC-MS	^{1}H-NMR
**23**	Chembridge	Chembridge	55119706	> 90%	LC-MS	^{1}H-NMR
**24**	Chembridge	Chembridge	73911779	> 90%	LC-MS	^{1}H-NMR
**25**	Chembridge	Chembridge	45983641	> 90%	LC-MS	^{1}H-NMR
**26**	Chembridge	Chembridge	93025608	> 90%	LC-MS	^{1}H-NMR
**27**	Chembridge	Chembridge	5788646	> 90%	LC-MS	^{1}H-NMR
**28**	Chembridge	Chembridge	6407567	> 90%	LC-MS	^{1}H-NMR
**29**	DrugBank	Sigma-Aldrich	17343	≥ 98%	CoA	CoA
**30**	DrugBank	Sigma-Aldrich	A7611	≥ 98%	CoA	CoA
**31**	DrugBank	Sigma-Aldrich	SML1811	≥ 98%	CoA	CoA
**32**	Enamine	Enamine	EN300-250911	95%	LC-MS	^{1}H-NMR
**33**	Enamine	Enamine	Z1336457514	90%	LC-MS	^{1}H-NMR
**34**	Enamine	Enamine	Z1622825787	90%	LC-MS	^{1}H-NMR
**35**	Enamine	Amatek Chemical	A-0615	≥ 98%	CoA	^{1}H-NMR
**36**	Enamine	Amatek Chemical	A-5185	≥ 98%	CoA	^{1}H-NMR
**37**	Sigma-Aldrich	Chem-Impex	07083	> 98%	CoA	^{1}H-NMR
**38**	Sigma-Aldrich	Chem-Impex	04721	> 99%	CoA	^{1}H-NMR
**39**	Sigma-Aldrich	Chem-Impex	01404	≥ 98%	CoA	^{1}H-NMR
**40**	Sigma-Aldrich	Chem-Impex	06071	≥ 98%	CoA	^{1}H-NMR
**41**	Sigma-Aldrich	Chem-Impex	07382	≥ 99%	CoA	^{1}H-NMR
**42**	Sigma-Aldrich	Amatek Chemical	A-3072	≥ 98%	CoA	^{1}H-NMR

Table S8. Specifications of screening compounds and method of verification, as provided by vendors. Purity stated by coupled liquid chromatography-mass spectrometry (LC-MS) or certificate of analysis (CoA). Identity confirmed by proton nuclear magnetic resonance (^{1}H-NMR) spectra or CoA.

FC94586521

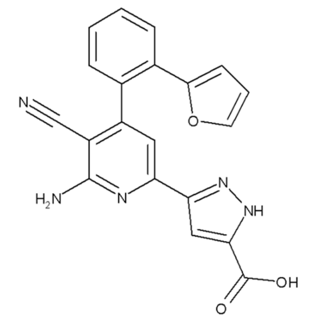

ID	90864634	371.3583	$C_{20}H_{13}N_5O_3$

```
Data File R:\HPLC\AUTO\FC945865\1EC-1101.D
Sample Name: Fc945865P1-E-03
Instrument 1 05/11/11 11:32:13
PMP1, Solvent A          : 0.1%TFA in Acn/H2O (2.5:97.5)
PMP1, Solvent B          : 0.1% TFA in AcN
PMP1, Solvent C          : 0.1%FA in ACN/H2O (2.5:97.5)
PMP1, Solvent D          : 0.1%FA in ACN
Ionization mode             :    APCI Positive

Signal 1: ADC1 A, ELSD
Peak RetTime Type  Width      Area       Height      Area
  #   [min]        [min]    [mV*s]       [mV]         %
----|-------|----|-------|----------|----------|--------|
   1   1.471 MM     0.0315  286.64450  151.57648 100.0000
Totals :                    286.64450  151.57648


Signal 2: DAD1 A, Sig=300,200 Ref=off
Peak RetTime Type  Width      Area       Height      Area
  #   [min]        [min]    [mAU*s]     [mAU]         %
----|-------|----|-------|----------|----------|--------|
   1   1.205 MM     0.0245   52.47095   35.76190   2.2034
   2   1.289 MF     0.0231   79.77810   57.44833   3.3501
   3   1.331 MF     0.0350  133.06870   63.36123   5.5879
   4   1.419 FM     0.0271 2116.05029 1301.64673  88.8586
Totals :                   2381.36804 1458.21819


Signal 3: MSD1 TIC, MS File
Peak RetTime Type  Width      Area       Height      Area
  #   [min]        [min]                              %
----|-------|----|-------|----------|----------|--------|
   1   1.475 MM     0.0484 1.17477e7  4.04130e6  100.0000
Totals :                   1.17477e7  4.04130e6
```

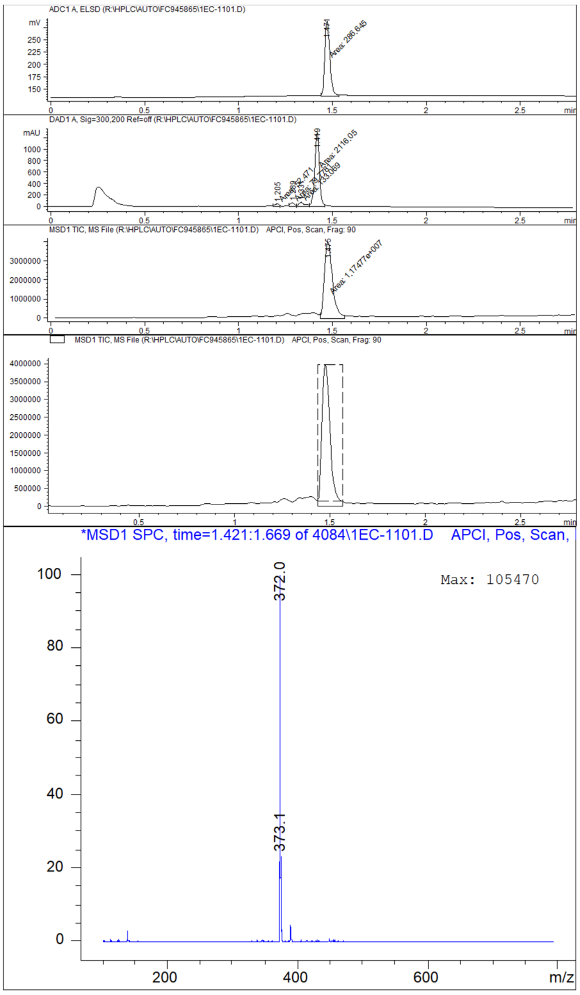

Figure S26. LC-MS spectrum of compound **13**.

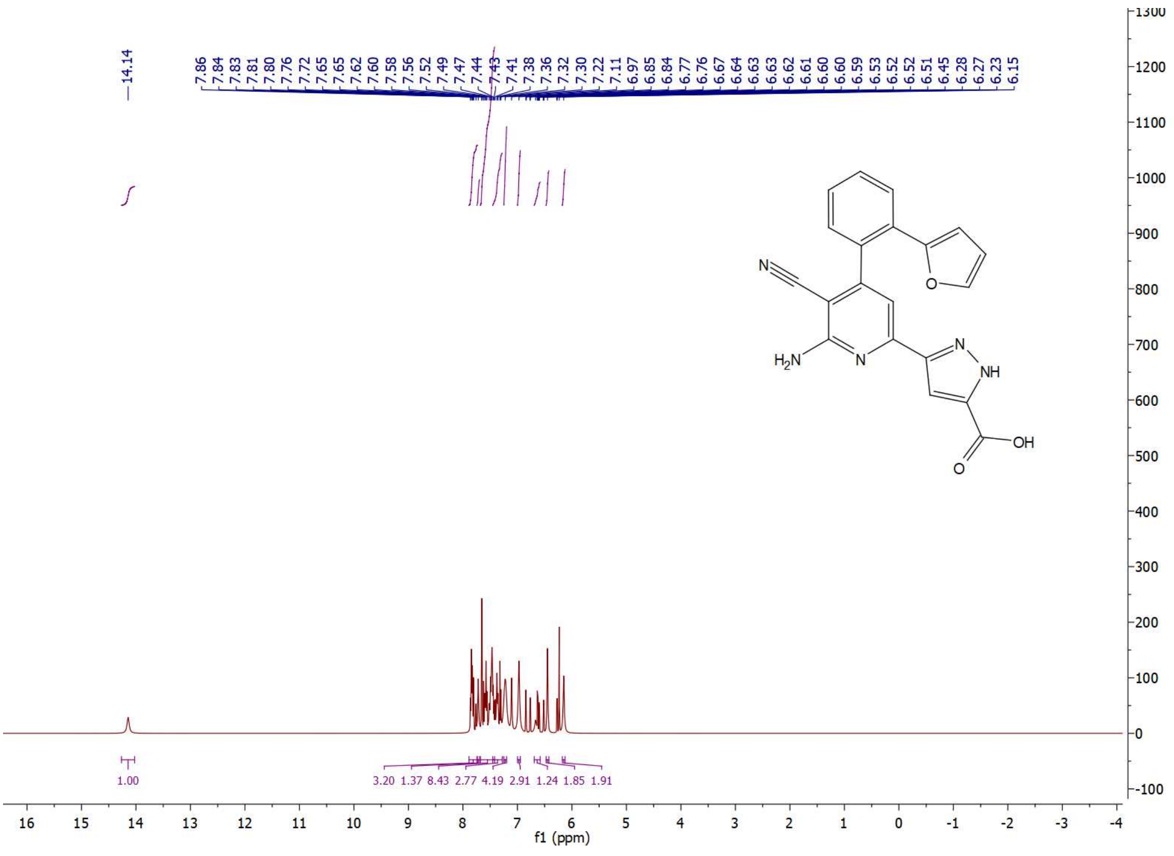

Figure S27. ^{1}H-NMR spectrum of compound **13**.

FC941404325

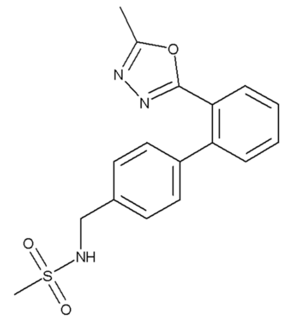

ID	17592342	343.4073	$C_{17}H_{17}N_3O_3S$

```
Data File D:\FC9414~1\2AD-2601.D
Sample Name: FC9414043P2-A-04
Instrument 1 14/12/2013 13:39:55
Column: Onyx C18 50x4.6mm | 3.75ml/min | Columns Reg Valve
Gradient: "A"->@2.2min->"B"(Hold 0.4min)->@0.2min->"A"->PostRun
PMP1, Solvent A          : 0.1%TFA, 2.5%AcN/W
PMP1, Solvent B          : 0.1%TFA/AcN
PMP1, Solvent C          : --NOT USED--
PMP1, Solvent D          : MeOH
Ionization mode               :    API-ES Positive

Signal 1: ADC1 A, ELSD
Peak RetTime Type  Width     Area       Height      Area
  #   [min]        [min]   [mV*s]      [mV]          %
----|-------|----|-------|----------|----------|--------|
   1  1.460 MM     0.0354  707.07800  332.74982 100.0000
Totals :                   707.07800  332.74982


Signal 2: DAD1 A, Sig=300,200 Ref=off
Peak RetTime Type  Width     Area       Height      Area
  #   [min]        [min]   [mAU*s]     [mAU]         %
----|-------|----|-------|----------|----------|--------|
   1  1.303 MM     0.0341   88.75000   43.38043   4.4562
   2  1.388 MM     0.0353 1902.85217  897.75653  95.5438
Totals :                  1991.60217  941.13696


Signal 3: MSD1 TIC, MS File
Peak RetTime Type  Width     Area       Height      Area
  #   [min]        [min]                             %
----|-------|----|-------|----------|----------|--------|
   1  1.405 MM     0.0532 3.71595e6  1.16452e6  100.0000
Totals :                  3.71595e6  1.16452e6
```

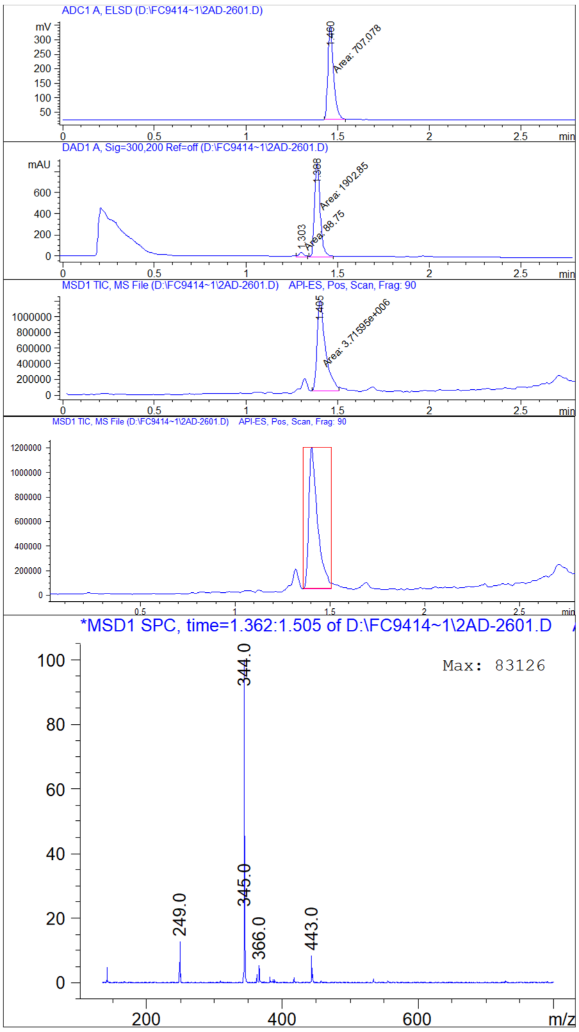

Figure S28. LC-MS spectrum of compound **14**.

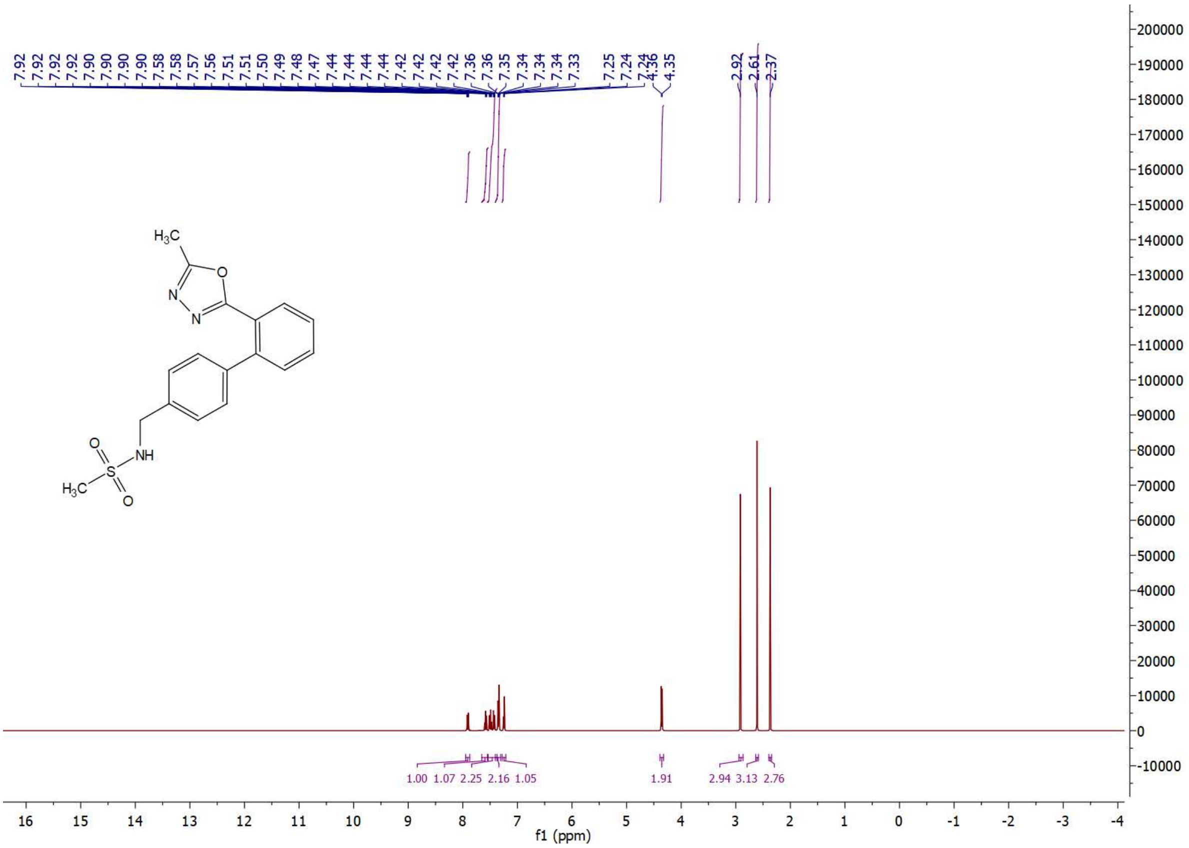

Figure S29. ^{1}H-NMR spectrum of compound **14**.

FC94108305

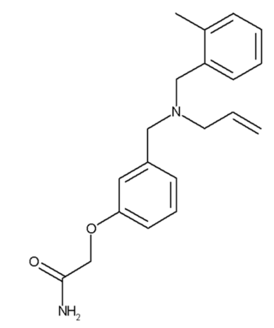

ID	75729207	324.4265	$C_{20}H_{24}N_2O_2$

```
Data File D:\DATA\2045\2EA-1501.D
Sample Name: Fc941083P2-E-01
Instrument 1 27/04/2010 15:14:51  #2
Column: Monolithic SpeedROD C18e 50x4.6mm | 3.75ml/min
Gradient: "A"->@2.1min->"B"(Hold 0.8min)->@0.2min->"A"->PostRun
PMP1, Solvent A          : 0.1%TFA in MeOH/H2O (2.5:97.5)
PMP1, Solvent B          : 0.1% TFA in MeOH
PMP1, Solvent C          : 0.1%FA in ACN/H2O (2.5:97.5)
PMP1, Solvent D          : 0.1%FA in ACN
Ionization mode              :    API-ES Positive

Signal 1: ADC1 A, ELSD
Peak RetTime Type  Width      Area       Height      Area
  #   [min]        [min]    [mV*s]       [mV]         %
----|-------|----|-------|----------|----------|--------|
   1   1.347 BP    0.0288   14.12710    7.54512 100.0000
Totals :                    14.12710    7.54512

Signal 2: DAD1 A, Sig=300,200 Ref=off
Peak RetTime Type  Width      Area       Height      Area
  #   [min]        [min]    [mAU*s]      [mAU]        %
----|-------|----|-------|----------|----------|--------|
   1   1.302 PB    0.0391  408.79758  158.50075 100.0000
Totals :                   408.79758  158.50075

Signal 3: MSD1 TIC, MS File
Peak RetTime Type  Width      Area       Height      Area
  #   [min]        [min]                              %
----|-------|----|-------|----------|----------|--------|
   1   1.357 BP    0.0507 3.84215e7  1.13136e7  100.0000
Totals :                  3.84215e7  1.13136e7
```

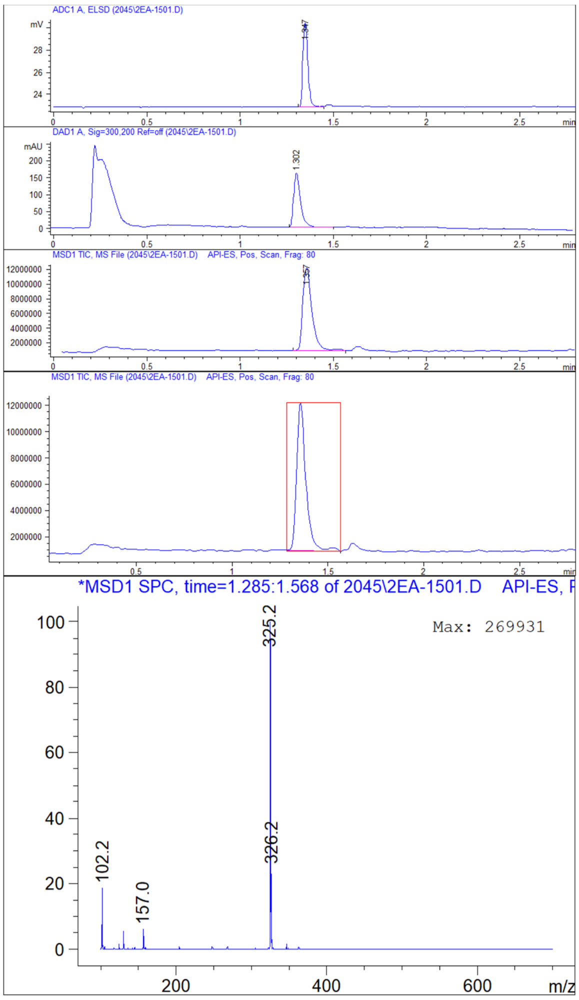

Figure S30. LC-MS spectrum of compound **15**.

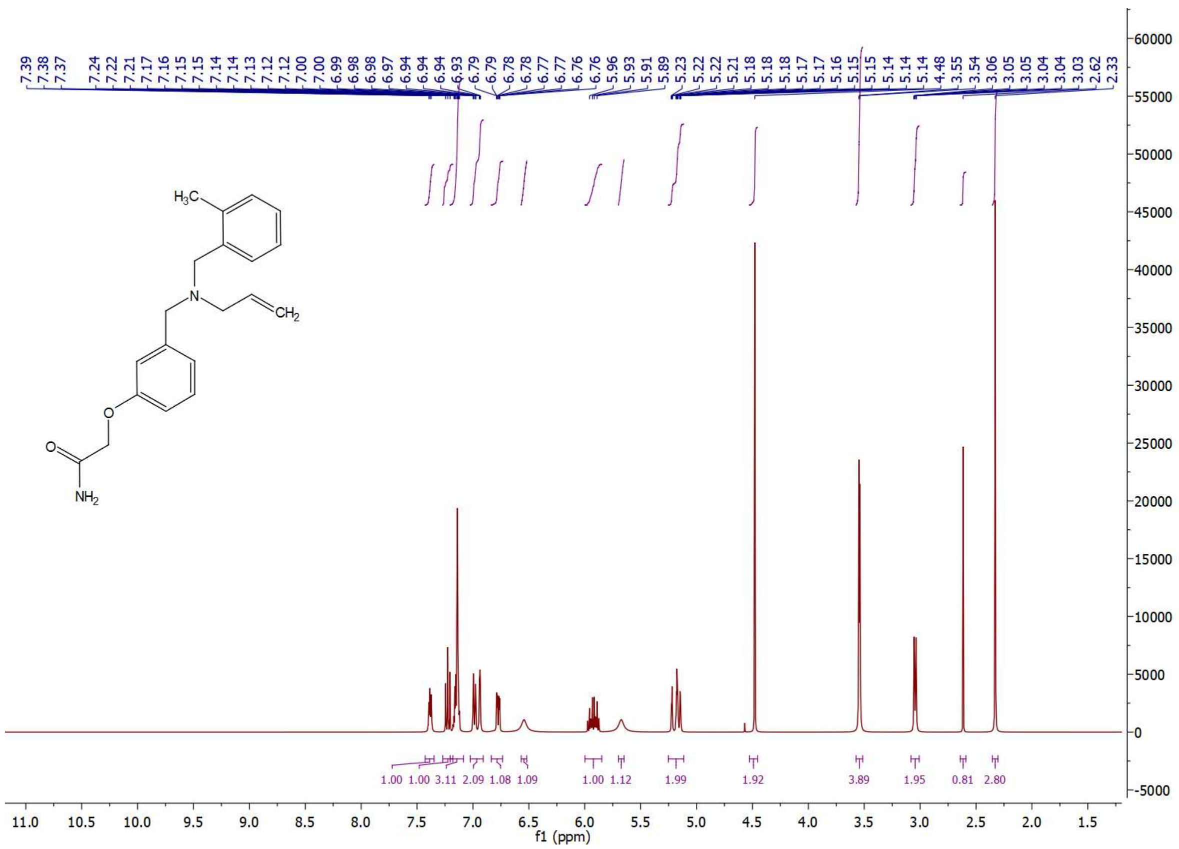

Figure S31. ^{1}H-NMR spectrum of compound **15**.

FC941619905

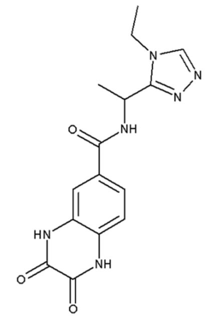

ID	69089454	328.3332	$C_{15}H_{16}N_6O_3$

Data File D:\FC9416~2\2EA-1101.D
Sample Name: FC9416199P2-E-01
Instrument 1 22/12/2014 17:24:11
Column: Onyx C18 50x4.6mm | 3.75ml/min | Columns Reg Valve
Gradient: "A"->@2.2min->"B"(Hold 0.4min)->@0.2min->"A"->PostRun
PMP1, Solvent A : 0.1%TFA in Acn/H2O (2.5:97.5)
PMP1, Solvent B : 0.1%TFA in AcN
PMP1, Solvent C : 0.1%FA in Acn/H2O (2.5:97.5)
PMP1, Solvent D : 0.1%FA in AcN
Ionization mode : APCI Positive

Signal 1: ADC1 B, ELSD

Peak #	RetTime [min]	Type	Width [min]	Area [mAu*s]	Height [mAu]	Area %
1	0.907	BB	0.0292	66.42889	36.50945	100.0000
Totals :				66.42889	36.50945	

Signal 2: DAD1 A, Sig=300,200 Ref=off

Peak #	RetTime [min]	Type	Width [min]	Area [mAU*s]	Height [mAU]	Area %
1	0.866	PB	0.0303	2160.26025	1129.71533	100.0000
Totals :				2160.26025	1129.71533	

Signal 3: MSD1 TIC, MS File

Peak #	RetTime [min]	Type	Width [min]	Area	Height	Area %
1	0.896	MM	0.0501	2.63097e4	8749.19434	100.0000
Totals :				2.63097e4	8749.19434	

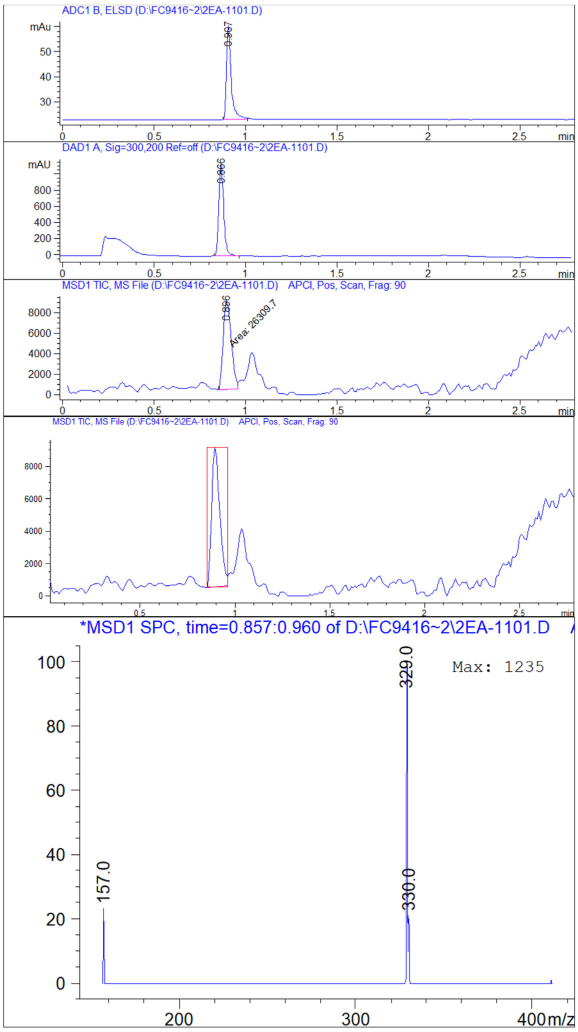

Figure S32. LC-MS spectrum of compound **16**.

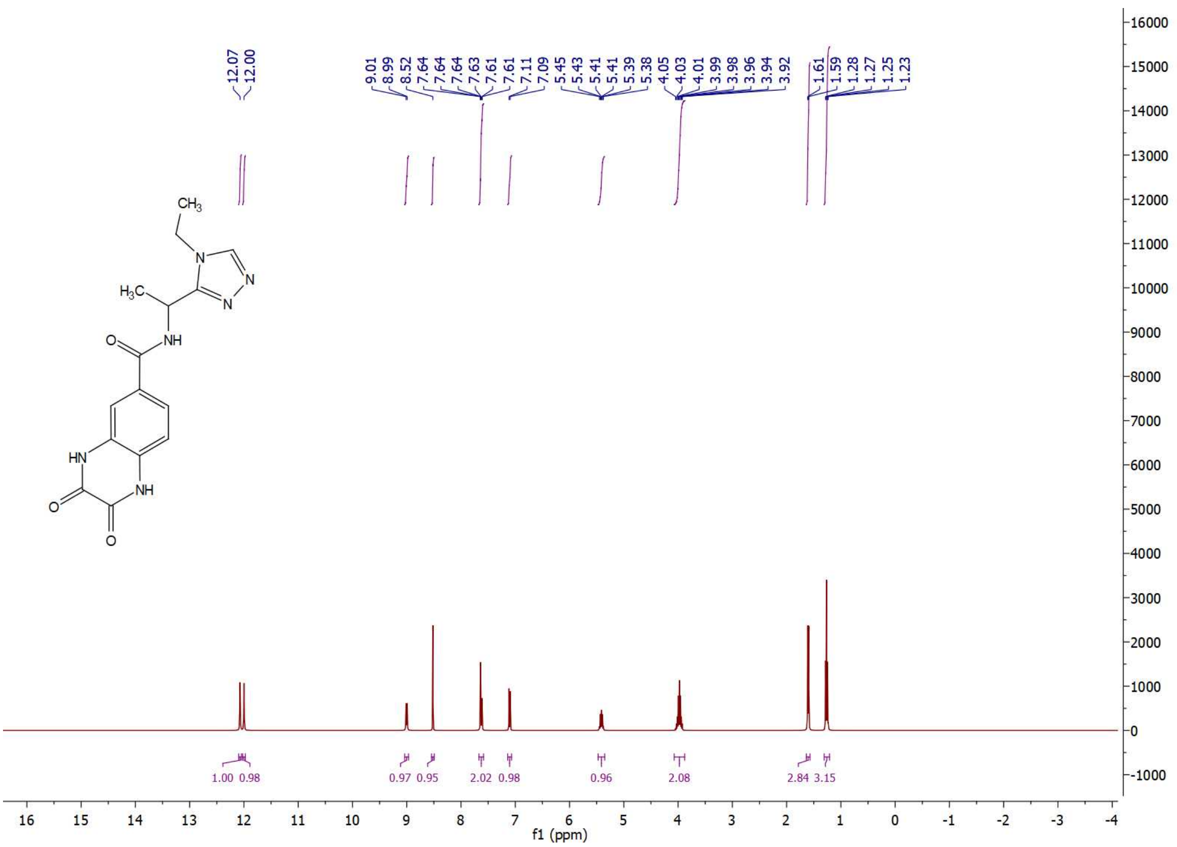

Figure S33. ^{1}H-NMR spectrum of compound **16**.

FC94124720

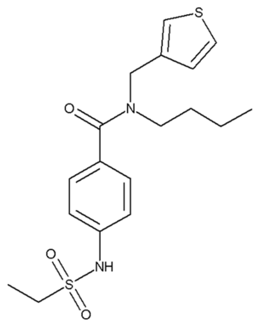

ID	42920737	380.5316	$C_{18}H_{24}N_2O_3S_2$

```
Data File D:\DATA\278\2DC-5101.D
Sample Name: Fc941247P2-D-03
Instrument 1 08.06.2010 3:17:15  #6
Column: SpeedROD Rp-18e 50x4.6mm | 3.75ml/min | Columns Reg
Valve
Gradient: "A"->@2.2min->"B"(Hold 0.4min)->@0.2min->"A"->PostRun
PMP1, Solvent A          : 0.1%TFA in Acn/H2O (2.5:97.5)
PMP1, Solvent B          : 0.1% TFA in AcN
PMP1, Solvent C          : 0.1%FA in ACN/H2O (2.5:97.5)
PMP1, Solvent D          : 0.1%FA in ACN
Ionization mode              :    APCI Positive

Signal 1: ADC1 A, ELSD
Peak RetTime Type  Width     Area       Height     Area
  #   [min]        [min]    [mV*s]      [mV]        %
----|-------|----|-------|----------|----------|--------|
   1   1.768  PB    0.0340 1076.39929  493.29245 100.0000
Totals :                   1076.39929  493.29245

Signal 2: DAD1 A, Sig=300,200 Ref=off
Peak RetTime Type  Width     Area       Height     Area
  #   [min]        [min]    [mAU*s]     [mAU]       %
----|-------|----|-------|----------|----------|--------|
   1   1.714  BB    0.0319 1487.78931  725.97333 100.0000
Totals :                   1487.78931  725.97333

Signal 3: MSD1 TIC, MS File
Peak RetTime Type  Width     Area       Height     Area
  #   [min]        [min]                            %
----|-------|----|-------|----------|----------|--------|
   1   1.737  PP    0.0496 1.29464e7  4.11518e6  100.0000
Totals :                   1.29464e7  4.11518e6
```

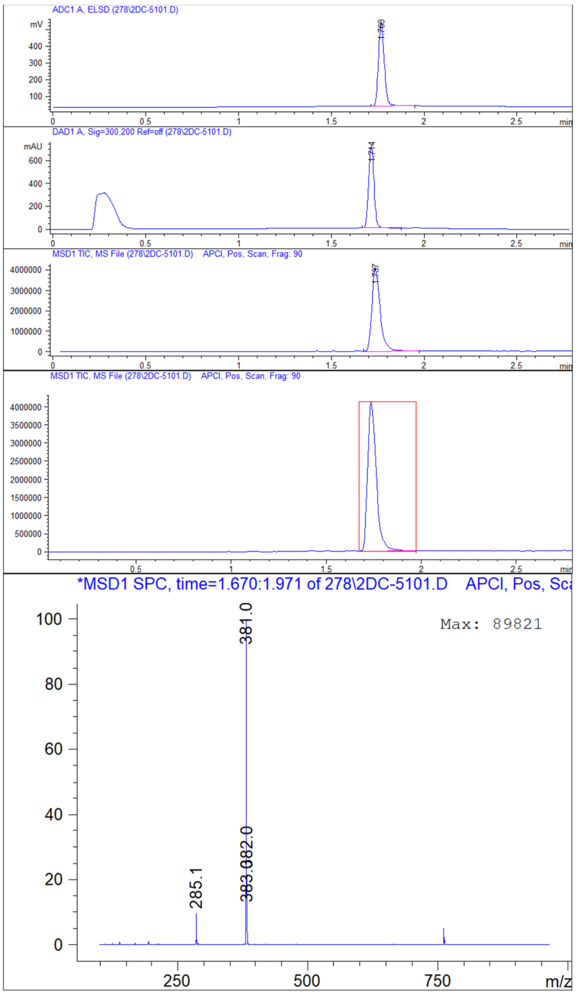

Figure S34. LC-MS spectrum of compound **17**.

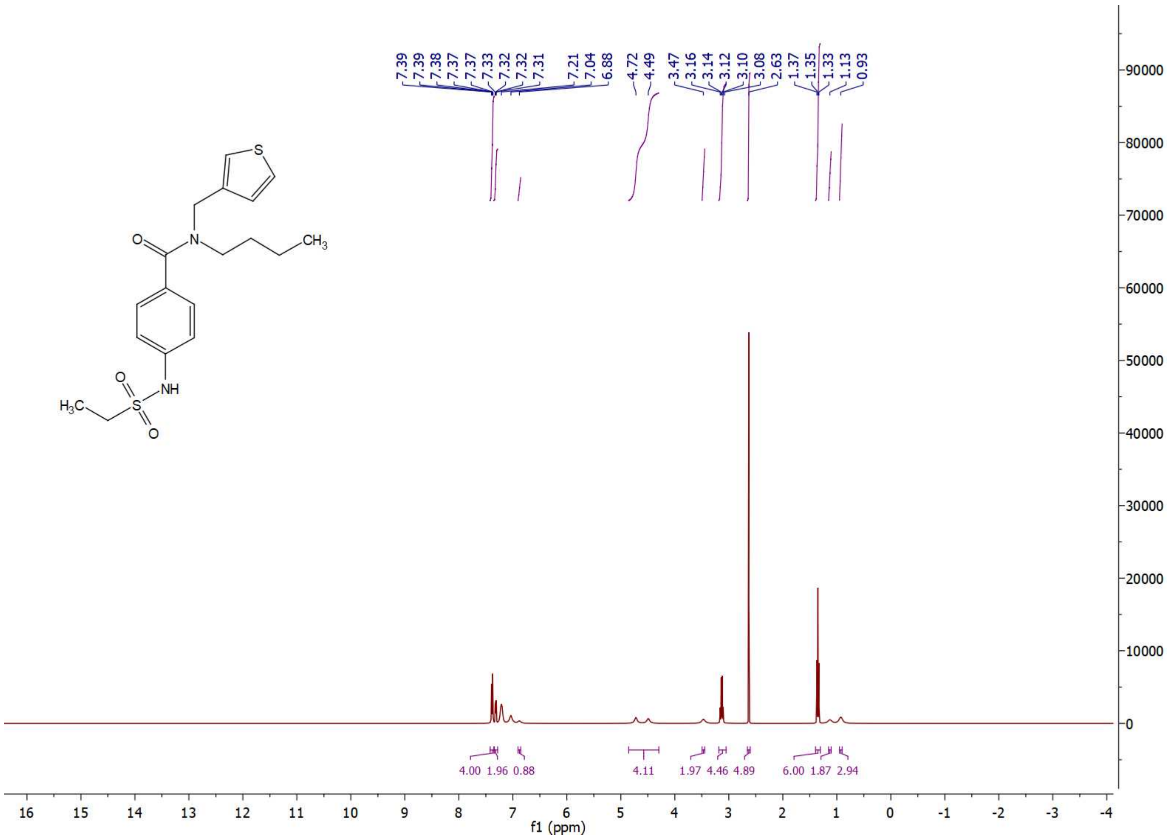

Figure S35. ^{1}H-NMR spectrum of compound **17**.

FC941415013

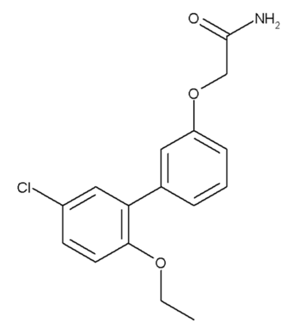

ID	59415756	305.7638	$C_{16}H_{16}ClNO_3$

```
Data File D:\DATA\FC9414~1\2EB-2801.D
Sample Name: FC9414150P2-E-02
Instrument 1 26/12/2013 10:13:41
Column: Onyx C18 50x4.6mm | 3.75ml/min | Columns Reg Valve
Gradient: "A"->@2.2min->"B"(Hold 0.4min)->@0.2min->"A"->PostRun
PMP1, Solvent A         : 0.1%FA in Acn/H2O (2.5:97.5)
PMP1, Solvent B         : 0.1%TFA in AcN
PMP1, Solvent C         : 0.1%TFA in ACN/H2O (2.5:97.5)
PMP1, Solvent D         : 0.1%FA in ACN
Ionization mode              :    APCI Positive

Signal 1: ADC1 A, ELSD
Peak RetTime Type  Width     Area       Height      Area
  #   [min]        [min]    [mV*s]      [mV]         %
----|-------|----|-------|----------|----------|--------|
   1   1.797 MF    0.0278  489.18277  293.74487  97.5391
   2   1.838 FM    0.0223   12.34192    9.21917   2.4609
Totals :                   501.52469  302.96404

Signal 2: DAD1 A, Sig=300,200 Ref=off
Peak RetTime Type  Width     Area       Height      Area
  #   [min]        [min]    [mAU*s]     [mAU]        %
----|-------|----|-------|----------|----------|--------|
   1   1.742 MM    0.0364 1350.10852  618.62280  97.9463
   2   2.129 MM    0.0266   28.30794   17.75657   2.0537
Totals :                  1378.41646  636.37937

Signal 3: MSD1 TIC, MS File
Peak RetTime Type  Width     Area       Height      Area
  #   [min]        [min]                             %
----|-------|----|-------|----------|----------|--------|
   1   1.775 MM    0.0298 4.26923e4  2.38645e4  100.0000
Totals :                  4.26923e4  2.38645e4
```

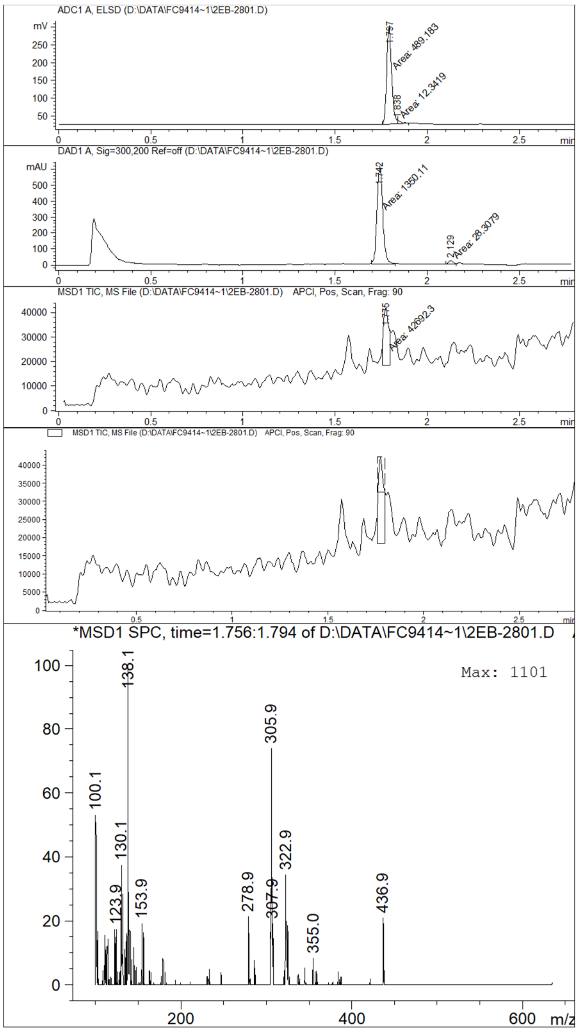

Figure S36. LC-MS spectrum of compound **18**.

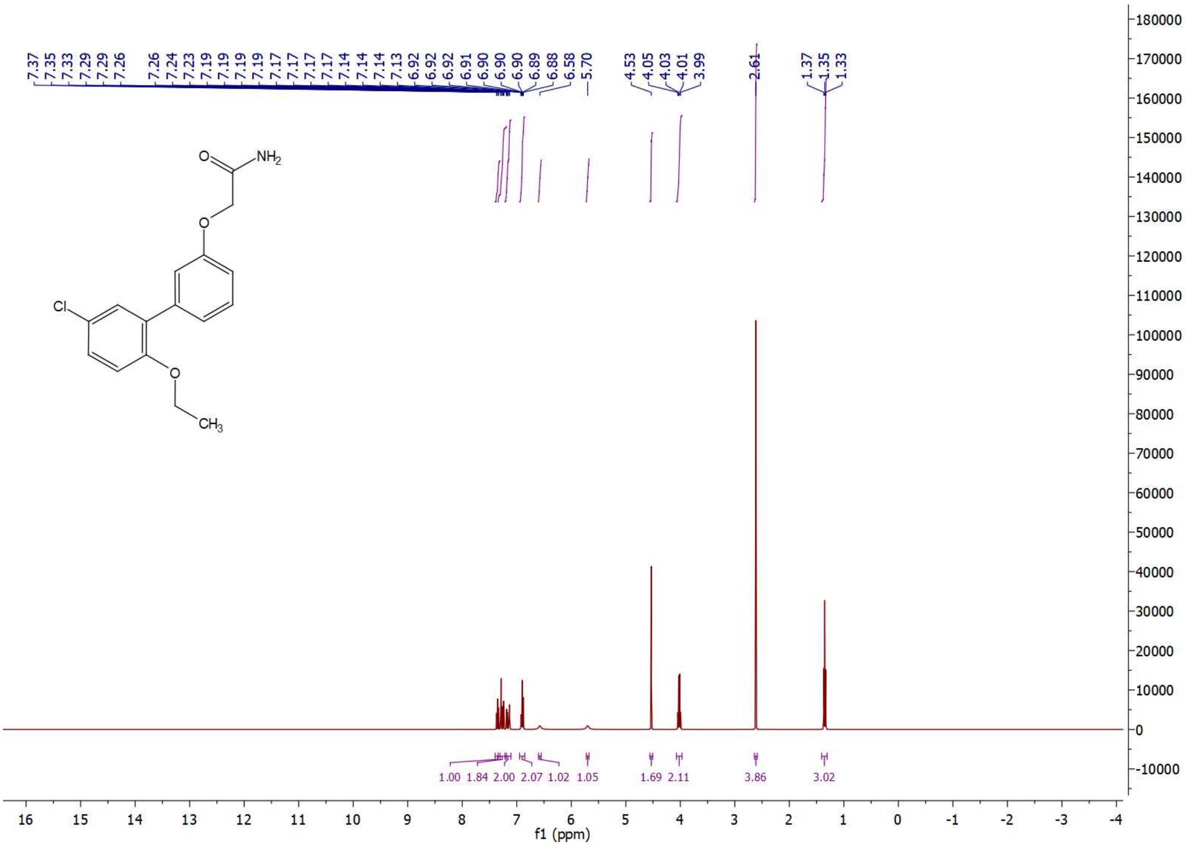

Figure S37. ^{1}H-NMR spectrum of compound **18**.

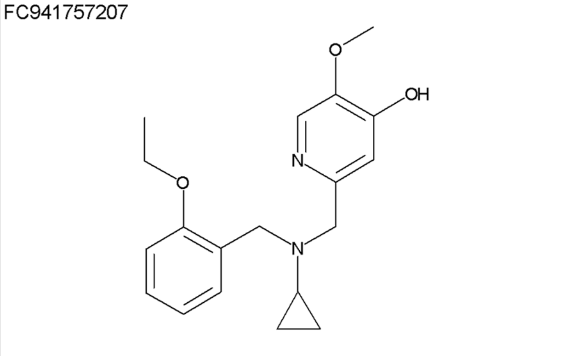

ID	96309693	328.4147	$C_{19}H_{24}N_2O_3$

```
Data File D:\FC9417~1\2GA-0801.D
Sample Name: FC9417572P2-G-01
Instrument 1 07/07/2015 12:06:38
Column: Onyx C18 50x4.6mm | 3.75ml/min | Columns Reg Valve
Gradient: "A"->@2.2min->"B"(Hold 0.4min)->@0.2min->"A"->PostRun
PMP1, Solvent A          : 0.1%TFA in Acn/H2O (2.5:97.5)
PMP1, Solvent B          : 0.1%TFA in AcN
PMP1, Solvent C          : 0.1%FA in Acn/H2O (2.5:97.5)
PMP1, Solvent D          : 0.1%FA in AcN
Ionization mode               :    APCI Positive

Signal 1: ADC1 B, ELSD
Peak RetTime Type  Width     Area      Height     Area
  #   [min]        [min]   [mAu*s]     [mAu]       %
----|-------|----|-------|----------|----------|--------|
   1   1.152 BB    0.0273   49.98822   28.68937 100.0000
Totals :                    49.98822   28.68937

Signal 2: DAD1 A, Sig=300,200 Ref=off
Peak RetTime Type  Width     Area      Height     Area
  #   [min]        [min]   [mAU*s]     [mAU]       %
----|-------|----|-------|----------|----------|--------|
   1   1.111 BB    0.0330  977.47913  455.76953 100.0000
Totals :                   977.47913  455.76953

Signal 3: MSD1 TIC, MS File
Peak RetTime Type  Width     Area      Height     Area
  #   [min]        [min]                           %
----|-------|----|-------|----------|----------|--------|
   1   1.142 MM    0.0536 1.33367e7  4.14469e6  100.0000
Totals :                  1.33367e7  4.14469e6
```

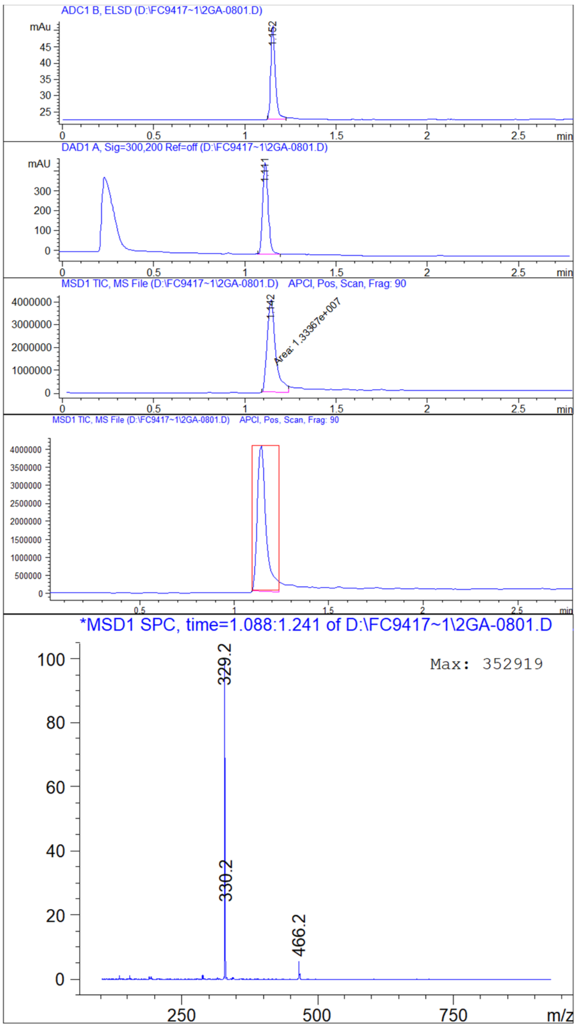

Figure S38. LC-MS spectrum of compound **19**.

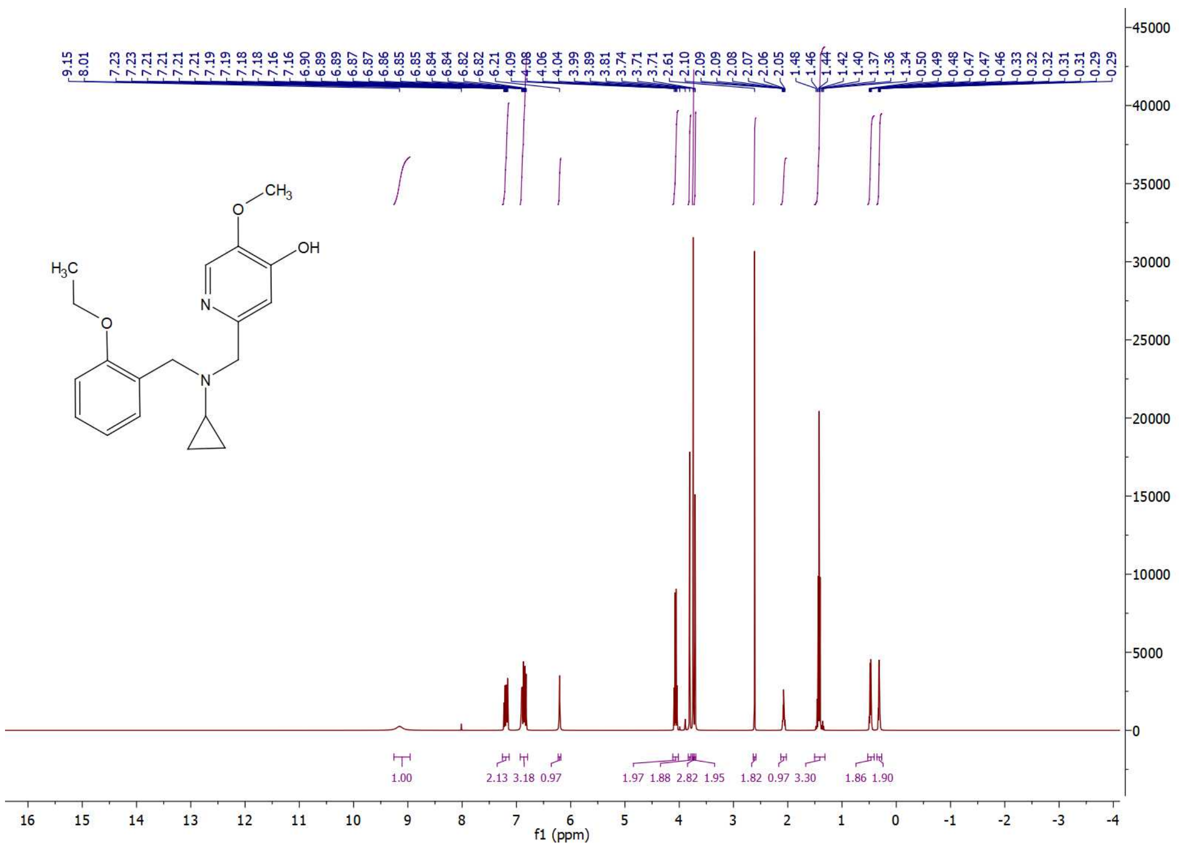

Figure S39. ^{1}H-NMR spectrum of compound **19**.

FC941604133

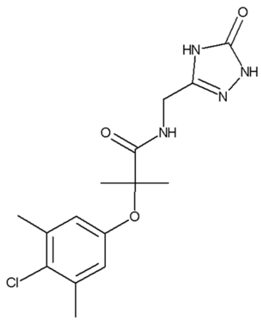

ID	93476697	338.7967	$C_{15}H_{19}ClN_4O_3$

```
Data File D:\DATA\363\2AE-3201.D
Sample Name: FC9416041P2-A-05
Instrument 1 13/09/2014 23:41:19  №6
Column: Luna C18 50x4.6mm | 3.75ml/min | Columns Reg Valve
Gradient: "A"->@2.2min->"B"(Hold 0.4min)->@0.2min->"A"->PostRun
PMP1, Solvent A          : 0.1%TFA in Acn/H2O (2.5:97.5)
PMP1, Solvent B          : 0.1%TFA in AcN
PMP1, Solvent C          : 0.1%FA in Acn/H2O (2.5:97.5)
PMP1, Solvent D          : 0.1%FA in AcN
Ionization mode               :    APCI Positive

Signal 1: ADC1 B, ELSD
Peak RetTime Type  Width      Area       Height      Area
  #   [min]         [min]    [mAu*s]     [mAu]        %
----|-------|----|-------|----------|----------|--------|
   1   1.501 PB     0.0257   58.83380   34.86125 100.0000
Totals :                     58.83380   34.86125


Signal 2: DAD1 A, Sig=300,200 Ref=off
Peak RetTime Type  Width      Area       Height      Area
  #   [min]         [min]    [mAU*s]     [mAU]        %
----|-------|----|-------|----------|----------|--------|
   1   1.460 BB     0.0330  465.83734  217.24261 100.0000
Totals :                    465.83734  217.24261


Signal 3: MSD1 TIC, MS File
Peak RetTime Type  Width      Area       Height      Area
  #   [min]         [min]                             %
----|-------|----|-------|----------|----------|--------|
   1   1.486 PB     0.0397 8.68557e5  3.23751e5  100.0000
Totals :                   8.68557e5  3.23751e5
```

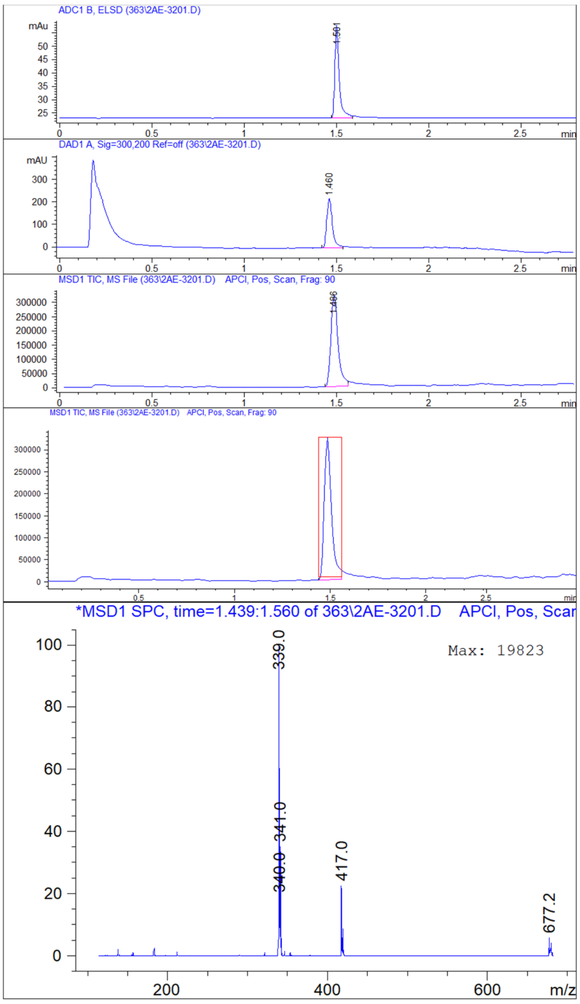

Figure S40. LC-MS spectrum of compound **20**.

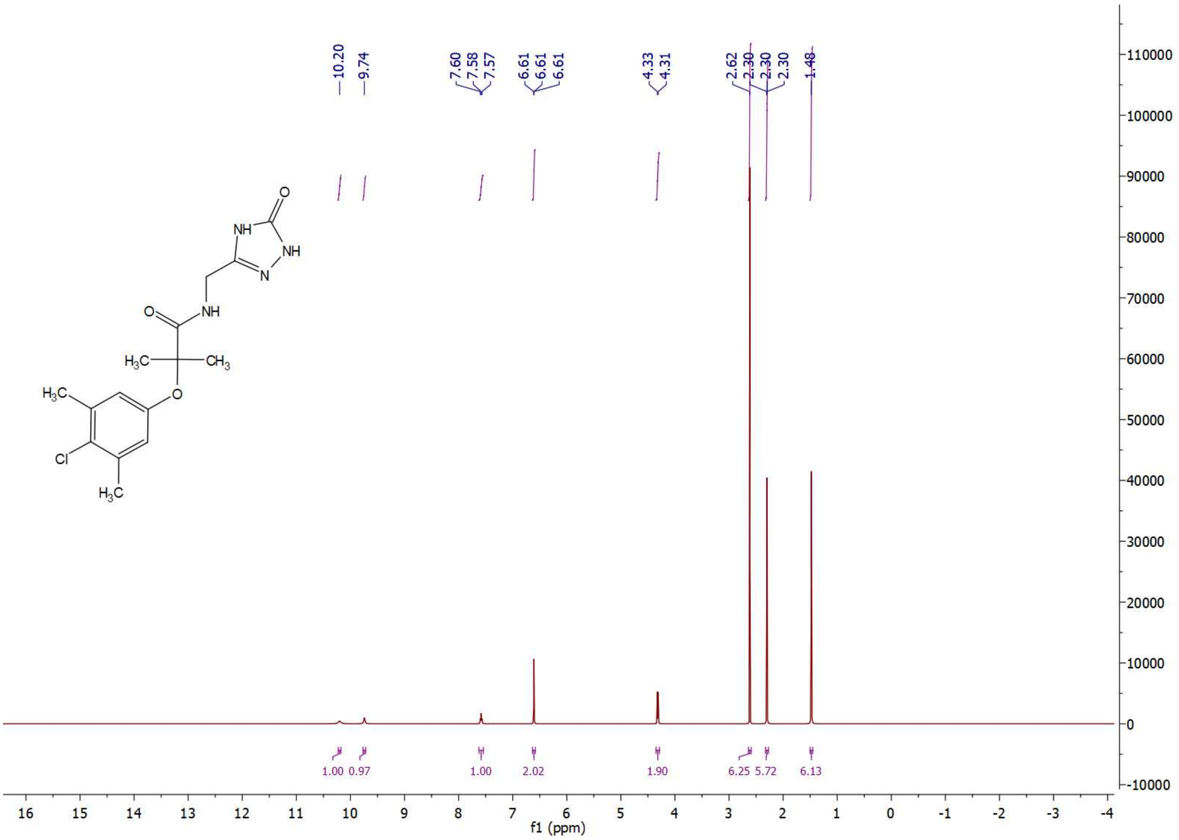

Figure S41. ^{1}H-NMR spectrum of compound **20**.

FC941627220

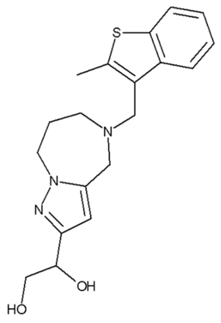

ID	61429699	357.4781	$C_{19}H_{23}N_3O_2S$

```
Data File R:\HPLC\AUTO\1DC-0401.D
Sample Name: FC9416272P1-D-03
Instrument 1 15/01/2015 17:08:59
Column: Onyx C18 50x4.6mm | 3.75ml/min | Columns Reg Valve
Gradient: "A"->@2.2min->"B"(Hold 0.4min)->@0.2min->"A"->PostRun
PMP1, Solvent A         : 0.1%TFA in Acn/H2O (2.5:97.5)
PMP1, Solvent B         : 0.1%TFA in AcN
PMP1, Solvent C         : 0.1%FA in Acn/H2O (2.5:97.5)
PMP1, Solvent D         : 0.1%FA in AcN
Ionization mode              :    APCI Positive

Signal 1: ADC1 B, ELSD
Peak RetTime Type  Width      Area       Height      Area
  #   [min]        [min]    [mAu*s]      [mAu]        %
----|-------|----|-------|----------|----------|--------|
   1   1.232 MM     0.0313   53.22203   28.34169 100.0000
Totals :                     53.22203   28.34169


Signal 2: DAD1 A, Sig=300,200 Ref=off
Peak RetTime Type  Width      Area       Height      Area
  #   [min]        [min]    [mAU*s]      [mAU]        %
----|-------|----|-------|----------|----------|--------|
   1   1.188 BB     0.0313 1048.66492  525.41290 100.0000
Totals :                   1048.66492  525.41290


Signal 3: MSD1 TIC, MS File
Peak RetTime Type  Width      Area       Height      Area
  #   [min]        [min]                              %
----|-------|----|-------|----------|----------|--------|
   1   1.221 MM     0.0481 1.44097e6  4.99496e5  100.0000
Totals :                   1.44097e6  4.99496e5
```

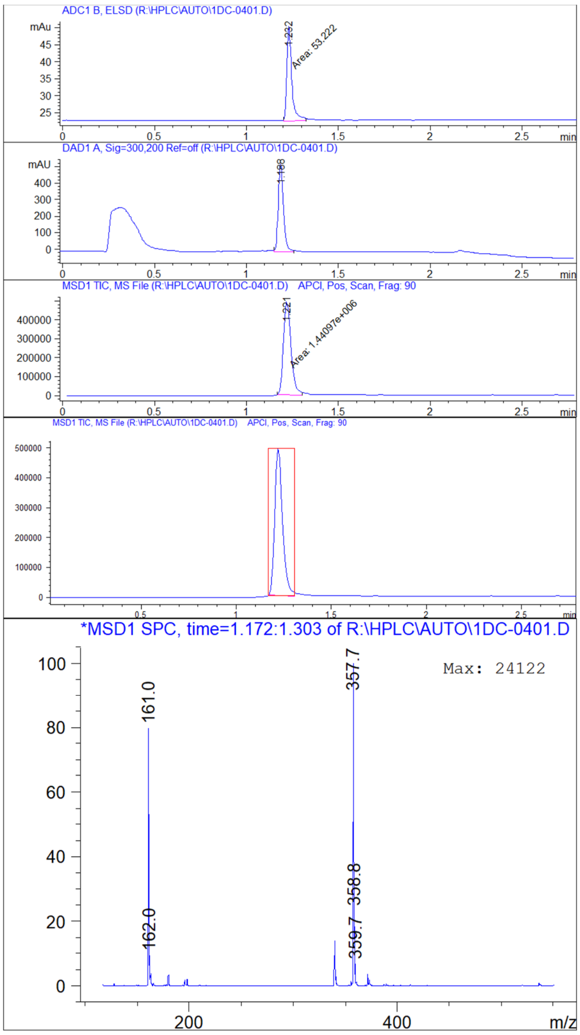

Figure S42. LC-MS spectrum of compound **21**.

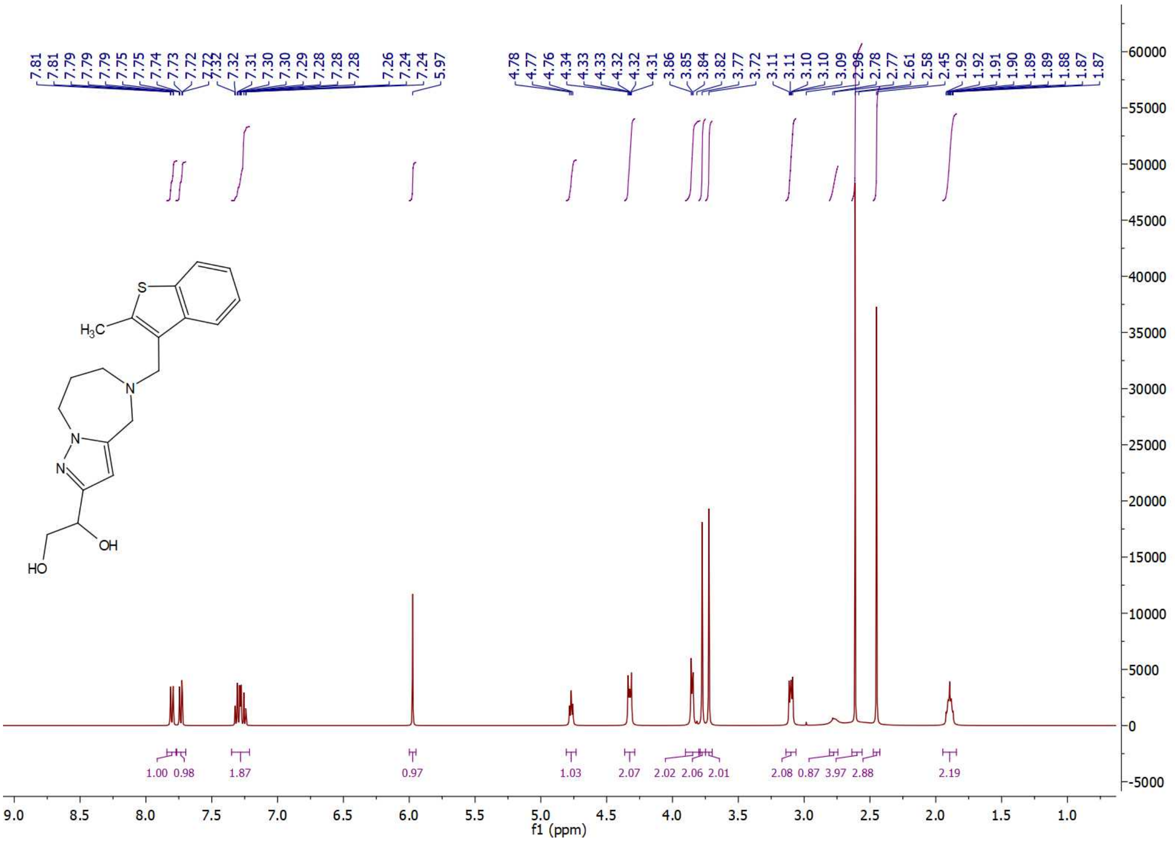

Figure S43. ^{1}H-NMR spectrum of compound **21**.

FC941471927

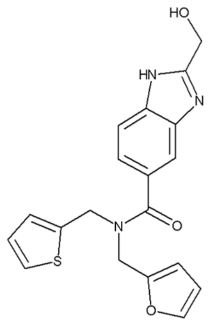

ID	56805360	367.4296	$C_{19}H_{17}N_3O_3S$

```
Data File D:\FC9414~1\2CD-3301.D
Sample Name: FC9414719P2-C-04
Instrument 1 21/02/2014 9:40:28
Column: Onyx C18 50x4.6mm | 3.75ml/min | Columns Reg Valve
Gradient: "A"->@2.2min->"B"(Hold 0.4min)->@0.2min->"A"->PostRun
PMP1, Solvent A          : --NOT USED--
PMP1, Solvent B          : 0.1%TFA/AcN
PMP1, Solvent C          : 0.1%TFA, 2.5%AcN/W
PMP1, Solvent D          : --NOT USED--
Ionization mode              :    API-ES Positive

Signal 1: ADC1 A, ELSD
Peak RetTime Type  Width      Area       Height      Area
  #   [min]        [min]    [mV*s]      [mV]          %
----|-------|----|-------|----------|----------|--------|
   1   1.031 MM     0.0434    6.14426    2.35735    3.3883
   2   1.351 MM     0.0382  167.33493   73.08236   92.2787
   3   1.412 MM     0.0328    7.85726    3.98911    4.3330
Totals :                    181.33646   79.42882

Signal 2: DAD1 A, Sig=300,200 Ref=off
Peak RetTime Type  Width      Area       Height      Area
  #   [min]        [min]    [mAU*s]     [mAU]         %
----|-------|----|-------|----------|----------|--------|
   1   0.953 MM     0.0295   79.31690   44.78701    7.9156
   2   1.273 MM     0.0308  848.50348  459.76584   84.6785
   3   1.349 MM     0.0294   74.20933   42.03251    7.4059
Totals :                   1002.02971  546.58535

Signal 3: MSD1 TIC, MS File
Peak RetTime Type  Width      Area       Height      Area
  #   [min]        [min]                               %
----|-------|----|-------|----------|----------|--------|
   1   1.290 MM     0.0425 1.75291e6  6.87326e5  100.0000
Totals :                   1.75291e6  6.87326e5
```

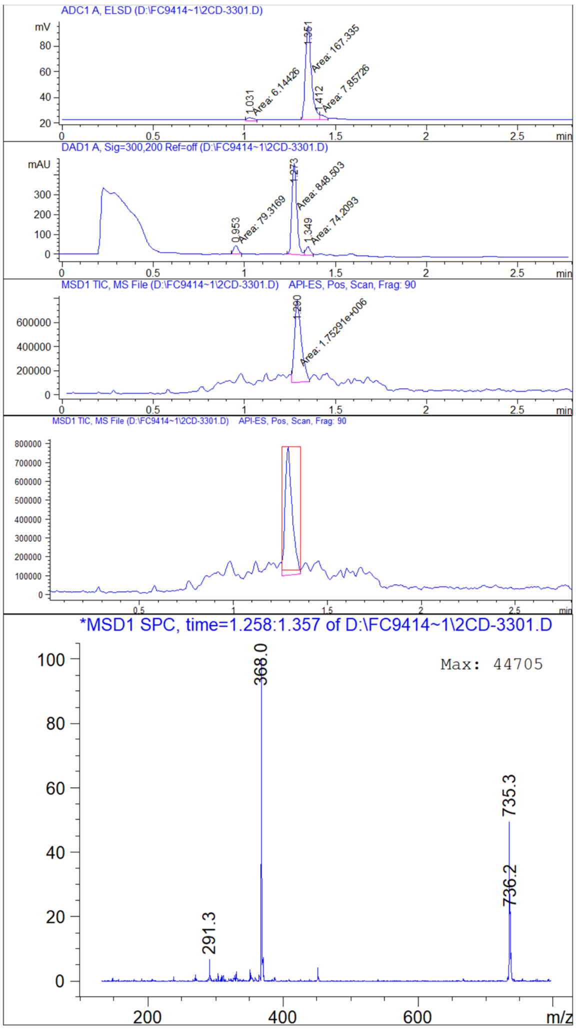

Figure S44. LC-MS spectrum of compound **22**.

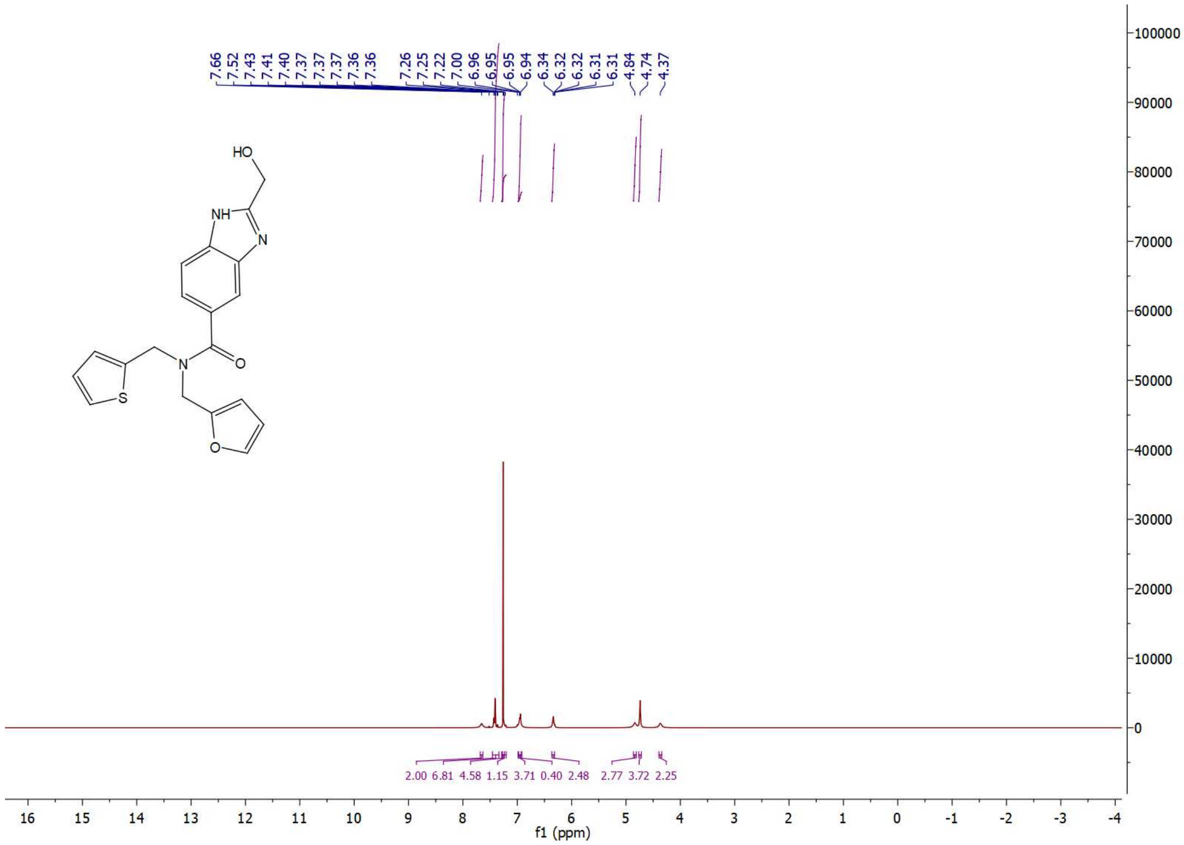

Figure S45. ^{1}H-NMR spectrum of compound **22**.

FC941218603

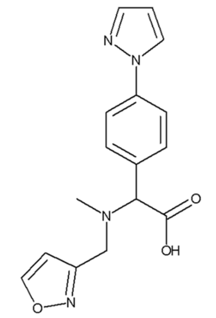

ID	55119706	312.3309	$C_{16}H_{16}N_4O_3$

```
Data File D:\DATA\30\2CA-0401.D
Sample Name: FC9412186P2-C-01
Instrument 1 15/06/2013 12:56:06  #4
Column: Onyx C18 50x4.6mm | 3.75ml/min | Columns Reg Valve
Gradient: "A"->@2.0min->"B"(Hold 0.4min)->@0.2min->"A"->PostRun
PMP1, Solvent A          : 0.1%TFA, 2.5%AcN/W
PMP1, Solvent B          : 0.1%TFA/AcN
PMP1, Solvent C          : --NOT USED--
PMP1, Solvent D          : MeOH
Ionization mode              :    API-ES Positive

Signal 1: ADC1 A, ELSD
Peak RetTime Type  Width      Area       Height      Area
  #   [min]        [min]    [mV*s]      [mV]          %
----|-------|----|-------|----------|----------|--------|
   1   0.795 PP     0.0390    34.94821   13.13548 100.0000
Totals :                      34.94821   13.13548


Signal 2: DAD1 A, Sig=300,200 Ref=off
Peak RetTime Type  Width      Area       Height      Area
  #   [min]        [min]    [mAU*s]     [mAU]         %
----|-------|----|-------|----------|----------|--------|
   1   0.715 PV     0.0375 2709.57349 1151.28125  95.8371
   2   0.811 VP     0.0297  117.69513   60.46548   4.1629
Totals :                    2827.26862 1211.74673


Signal 3: MSD1 TIC, MS File
Peak RetTime Type  Width      Area       Height      Area
  #   [min]        [min]                              %
----|-------|----|-------|----------|----------|--------|
   1   0.730 BP     0.0398 1.48016e7  5.78066e6  100.0000
Totals :                    1.48016e7  5.78066e6
```

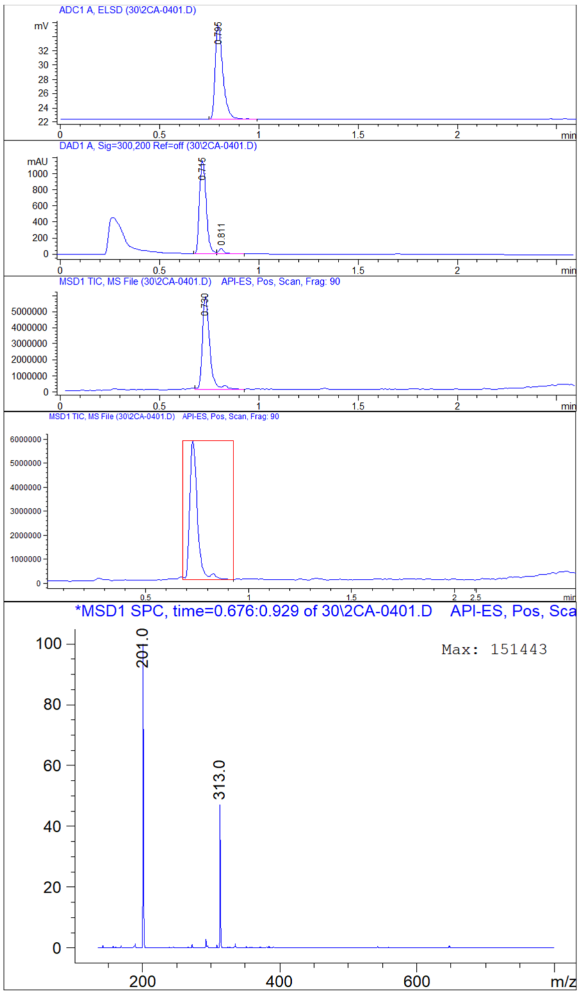

Figure S46. LC-MS spectrum of compound **23**.

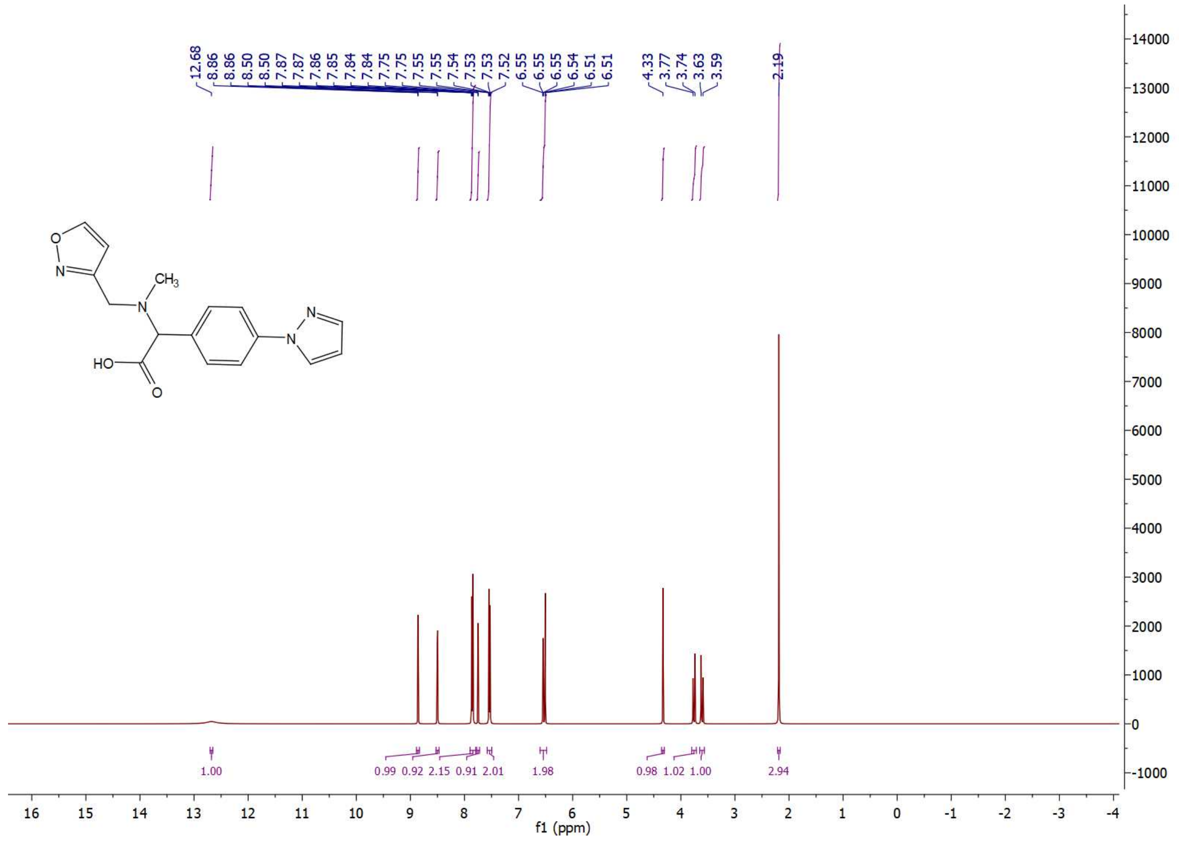

Figure S47. ^{1}H-NMR spectrum of compound **23**.

FC941267101

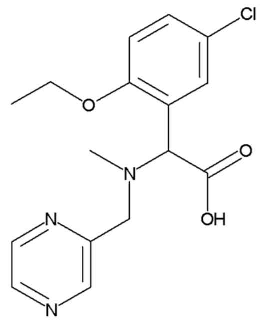

ID	73911779	335.7932	$C_{16}H_{18}ClN_3O_3$

```
Data File D:\FC9412~2\2AA-0401.D
Sample Name: FC9412671P2-A-01
Instrument 1 24/07/13 15:15:15
Column: Onyx C18 50x4.6mm | 3.75ml/min | Columns Reg Valve
Gradient: "A"->@2.2min->"B"(Hold 0.4min)->@0.2min->"A"->PostRun
PMP1, Solvent A         : 0.1%TFA, 2.5%AcN/W
PMP1, Solvent B         : 0.1%TFA/AcN
PMP1, Solvent C         : --NOT USED--
PMP1, Solvent D         : MeOH
Ionization mode              :    API-ES Positive

Signal 1: ADC1 A, ELSD
Peak RetTime Type  Width     Area      Height     Area
  #   [min]        [min]   [mV*s]      [mV]         %
----|-------|----|-------|----------|----------|--------|
   1   1.045 MM     0.0333    1.71163 8.57155e-1 100.0000
Totals :                      1.71163 8.57155e-1

Signal 2: DAD1 A, Sig=300,200 Ref=off
Peak RetTime Type  Width     Area      Height     Area
  #   [min]        [min]   [mAU*s]     [mAU]        %
----|-------|----|-------|----------|----------|--------|
   1   0.972 MM     0.0297  974.59967  546.66229 100.0000
Totals :                    974.59967  546.66229

Signal 3: MSD1 TIC, MS File
Peak RetTime Type  Width     Area      Height     Area
  #   [min]        [min]                            %
----|-------|----|-------|----------|----------|--------|
   1   0.995 MM     0.0349 7.87136e6  3.76167e6  100.0000
Totals :                   7.87136e6  3.76167e6
```

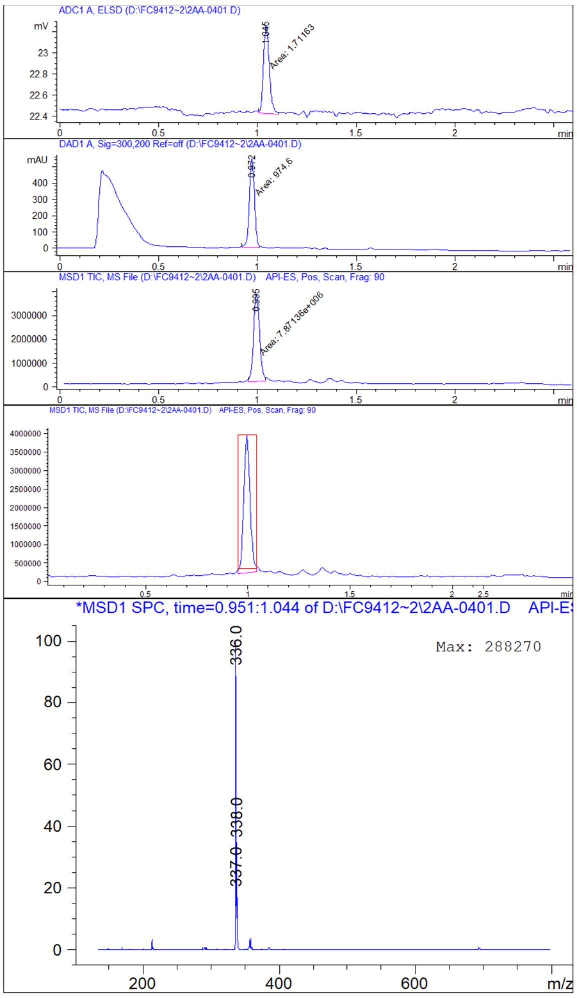

Figure S48. LC-MS spectrum of compound **24**.

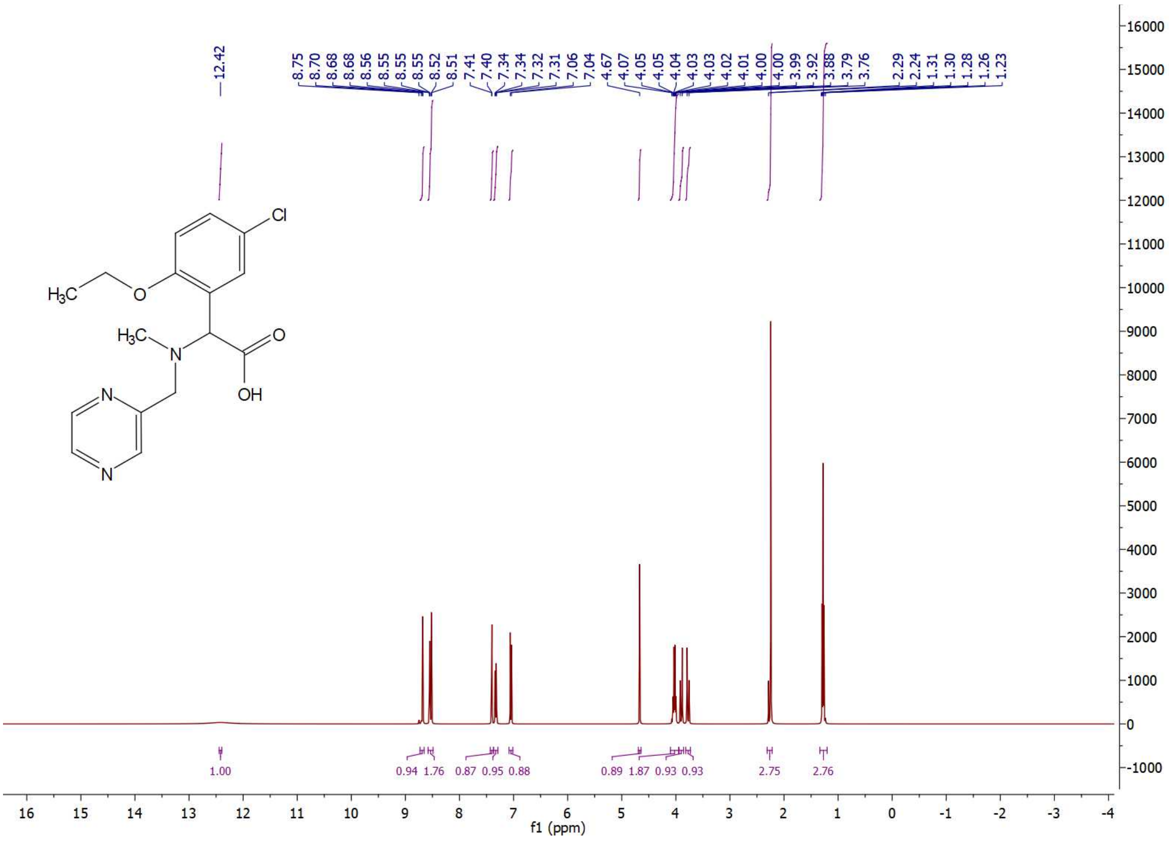

Figure S49. ^{1}H-NMR spectrum of compound **24**.

FC94419515

Chiral

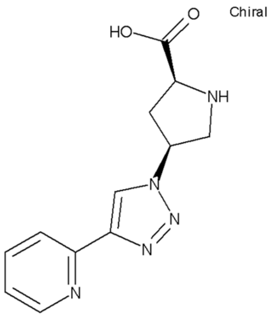

ID	45983641	259.2697	$C_{12}H_{13}N_5O_2$

```
Data File R:\HPLC\AUTO\FC944195\1GB-1401.D
Sample Name: Fc944195P1-G-02
Instrument 1 21/05/2011 12:33:21
Column: Onyx C18 50x4.6mm | 3.75ml/min | Columns Reg Valve
Gradient: "A"->@2.4min->"B"(Hold 0.2min)->@0.2min->"A"->PostRun
PMP1, Solvent A        : 0.1%TFA in Acn/H2O (2.5:97.5)
PMP1, Solvent B        : 0.1% TFA in AcN
PMP1, Solvent C        : 0.1%FA in ACN/H2O (2.5:97.5)
PMP1, Solvent D        : 0.1%FA in ACN
Ionization mode             :    API-ES Positive

Signal 1: ADC1 A, ELSD
Peak RetTime Type  Width     Area      Height     Area
  #   [min]        [min]   [mV*s]      [mV]        %
----|-------|----|-------|----------|----------|--------|
   1   0.505 PB    0.0423   55.47018   19.42817 100.0000
Totals :                    55.47018   19.42817

Signal 2: DAD1 A, Sig=300,200 Ref=off
Peak RetTime Type  Width     Area      Height     Area
  #   [min]        [min]   [mAU*s]     [mAU]       %
----|-------|----|-------|----------|----------|--------|
   1   0.432 MM    0.0462 1256.11218  453.29834  95.5373
   2   0.965 MM    0.0273   58.67499   35.82868   4.4627
Totals :                  1314.78717  489.12702

Signal 3: MSD1 TIC, MS File
Peak RetTime Type  Width     Area      Height     Area
  #   [min]        [min]                           %
----|-------|----|-------|----------|----------|--------|
   1   0.512 MM    0.0654 4.18275e6  1.06569e6  100.0000
Totals :                  4.18275e6  1.06569e6
```

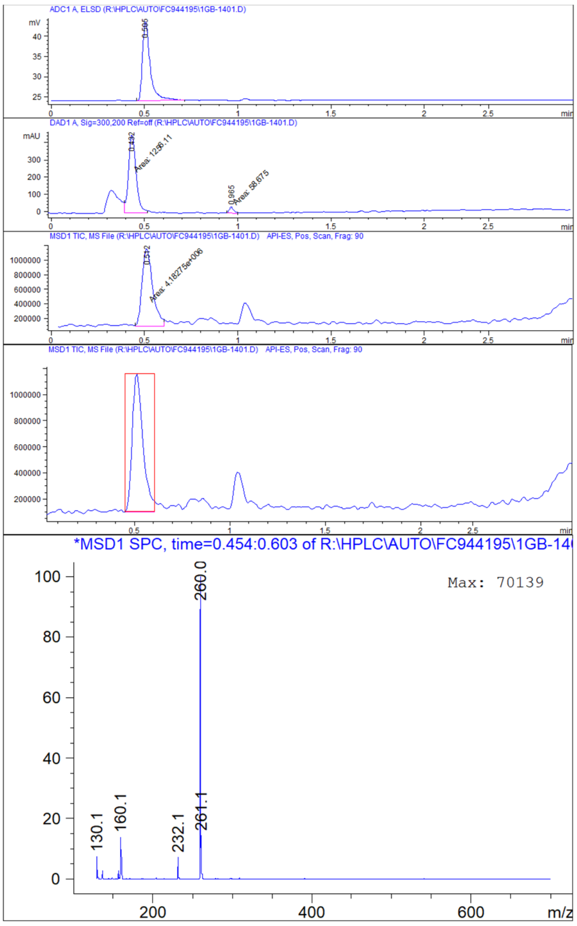

Figure S50. LC-MS spectrum of compound **25**.

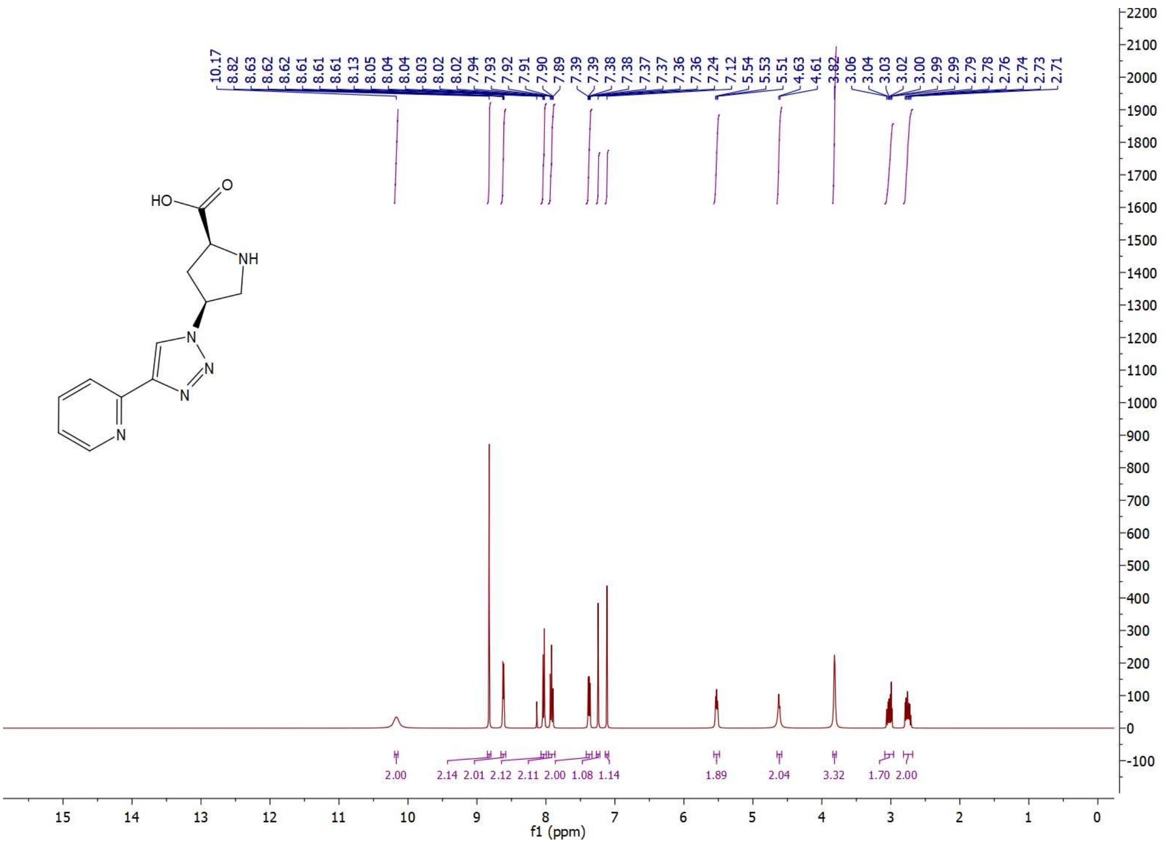

Figure S51. ^{1}H-NMR spectrum of compound **25**.

FC941270336

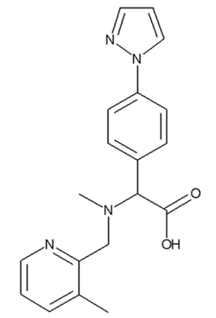

ID	93025608	336.3969	$C_{19}H_{20}N_4O_2$

```
Data File D:\DATA\456\2DE-3401.D
Sample Name: FC9412703P2-D-05
Instrument 1 27/07/2013 06:25:24
Column: Onyx C18 50x4.6mm | 3.75ml/min | Columns Reg Valve
Gradient: "A"->@2.0min->"B"(Hold 0.4min)->@0.2min->"A"->PostRun
PMP1, Solvent A         : 0.1%TFA, 2.5%AcN/W
PMP1, Solvent B         : 0.1%TFA/AcN
PMP1, Solvent C         : --NOT USED--
PMP1, Solvent D         : MeOH
Ionization mode               :    API-ES Positive

Signal 1: ADC1 A, ELSD
Peak RetTime Type  Width     Area       Height     Area
  #   [min]         [min]   [mV*s]      [mV]        %
----|-------|----|-------|----------|----------|--------|
   1   1.112 BP    0.0392     2.72832    1.08963 100.0000
Totals :                      2.72832    1.08963


Signal 2: DAD1 A, Sig=300,200 Ref=off
Peak RetTime Type  Width     Area       Height     Area
  #   [min]         [min]   [mAU*s]     [mAU]       %
----|-------|----|-------|----------|----------|--------|
   1   1.049 BB    0.0463 2621.05493  890.28906 100.0000
Totals :                  2621.05493  890.28906


Signal 3: MSD1 TIC, MS File
Peak RetTime Type  Width     Area       Height     Area
  #   [min]         [min]                           %
----|-------|----|-------|----------|----------|--------|
   1   1.066 BP    0.0457 9.78137e6  3.33974e6  100.0000
Totals :                  9.78137e6  3.33974e6
```

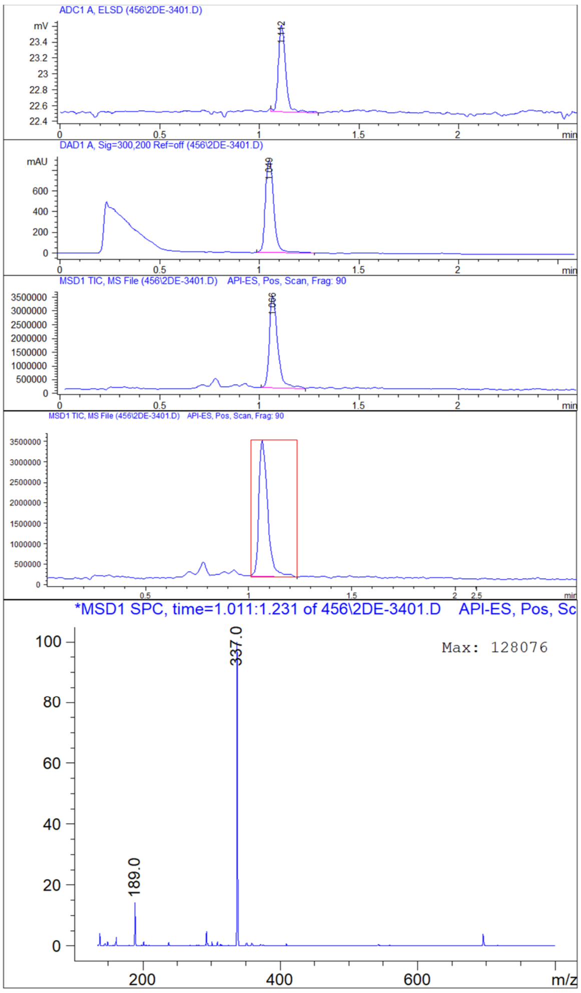

Figure S52. LC-MS spectrum of compound **26**.

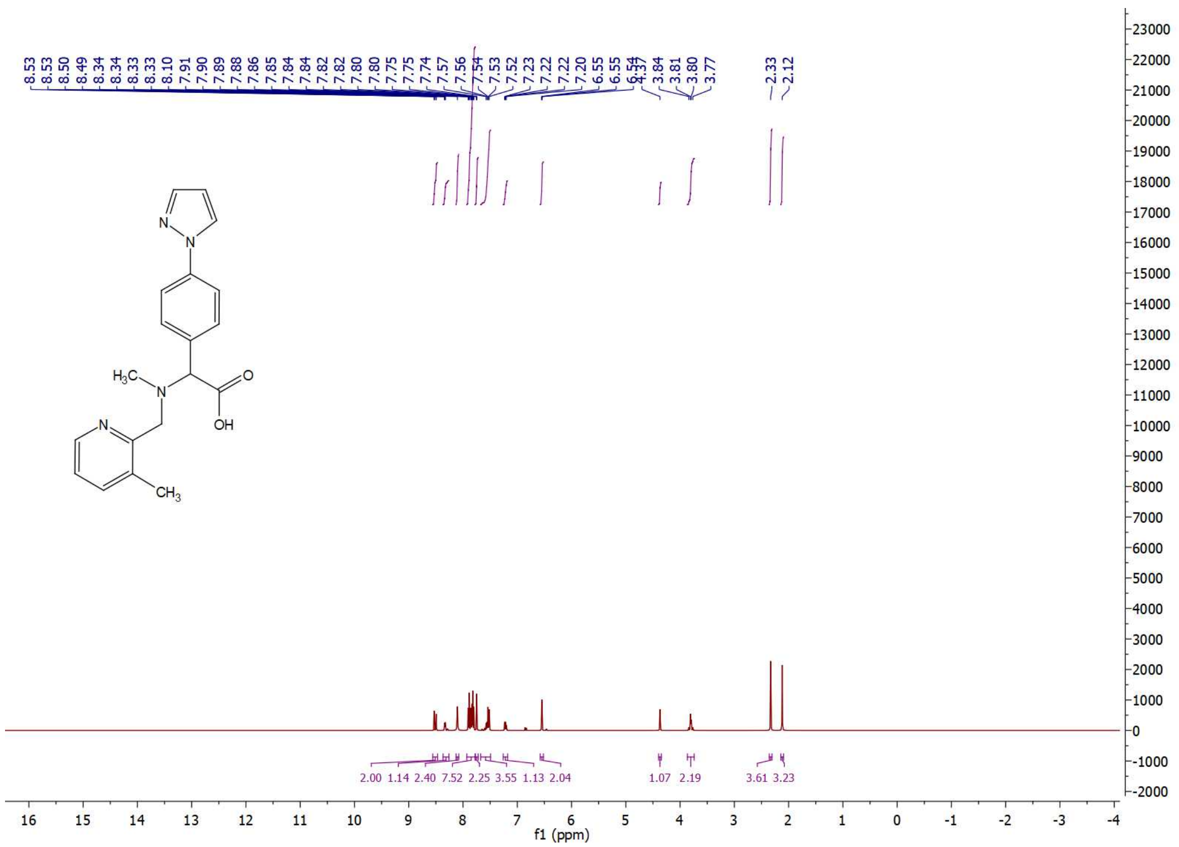

Figure S53. ^{1}H-NMR spectrum of compound **26**.

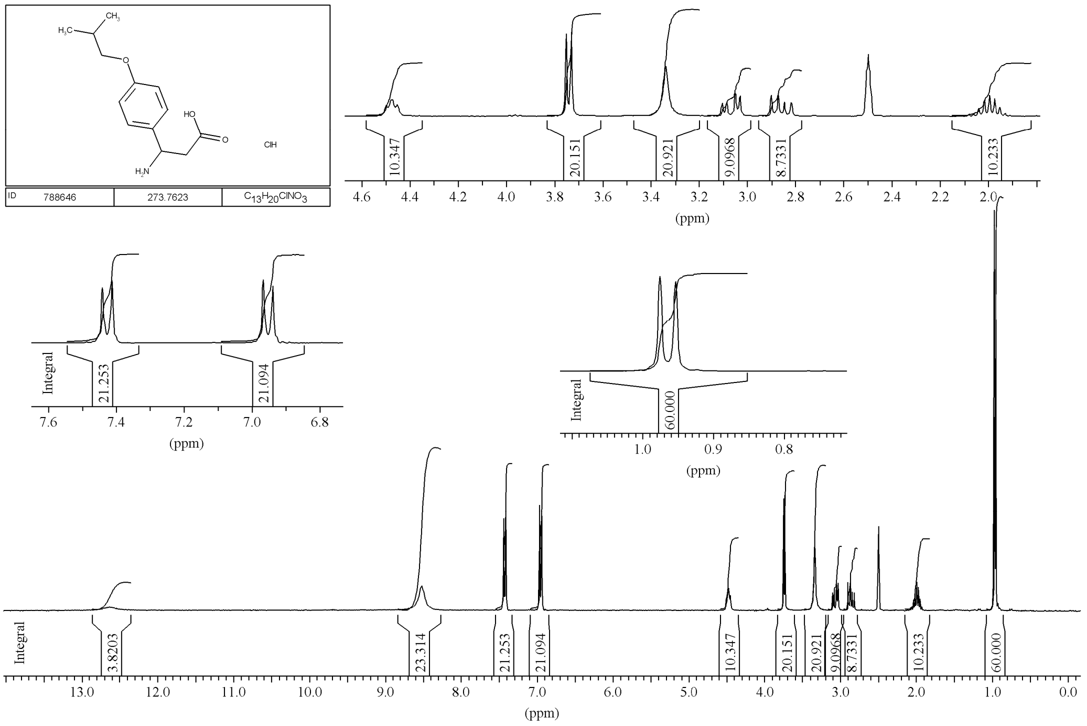

Figure S54. ^{1}H-NMR spectrum of compound **27**.

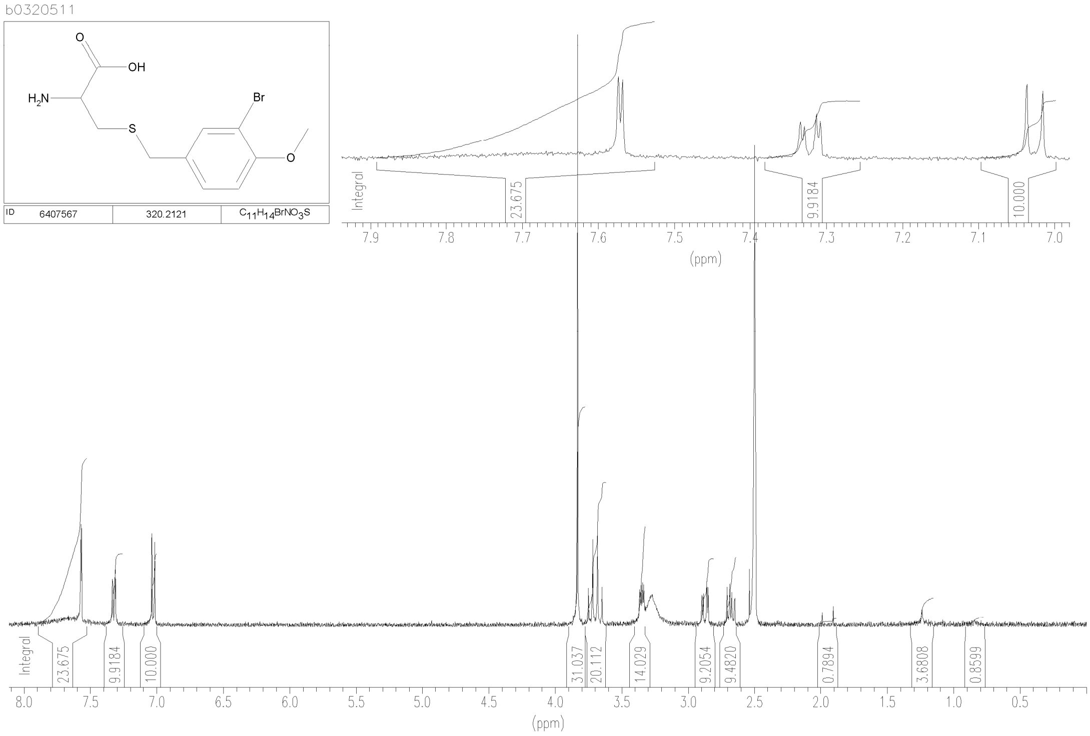

Figure S55. ^{1}H-NMR spectrum of compound **28**.

MaxPeak: 100.00%
Ret_Time: 0.867 min

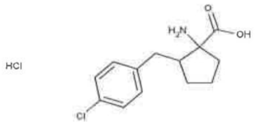

Mol Wt 290.19
Exact Mass 253.11

#	Time	Area%
1	0.867	100.00

RT 0.903

RT 0.897

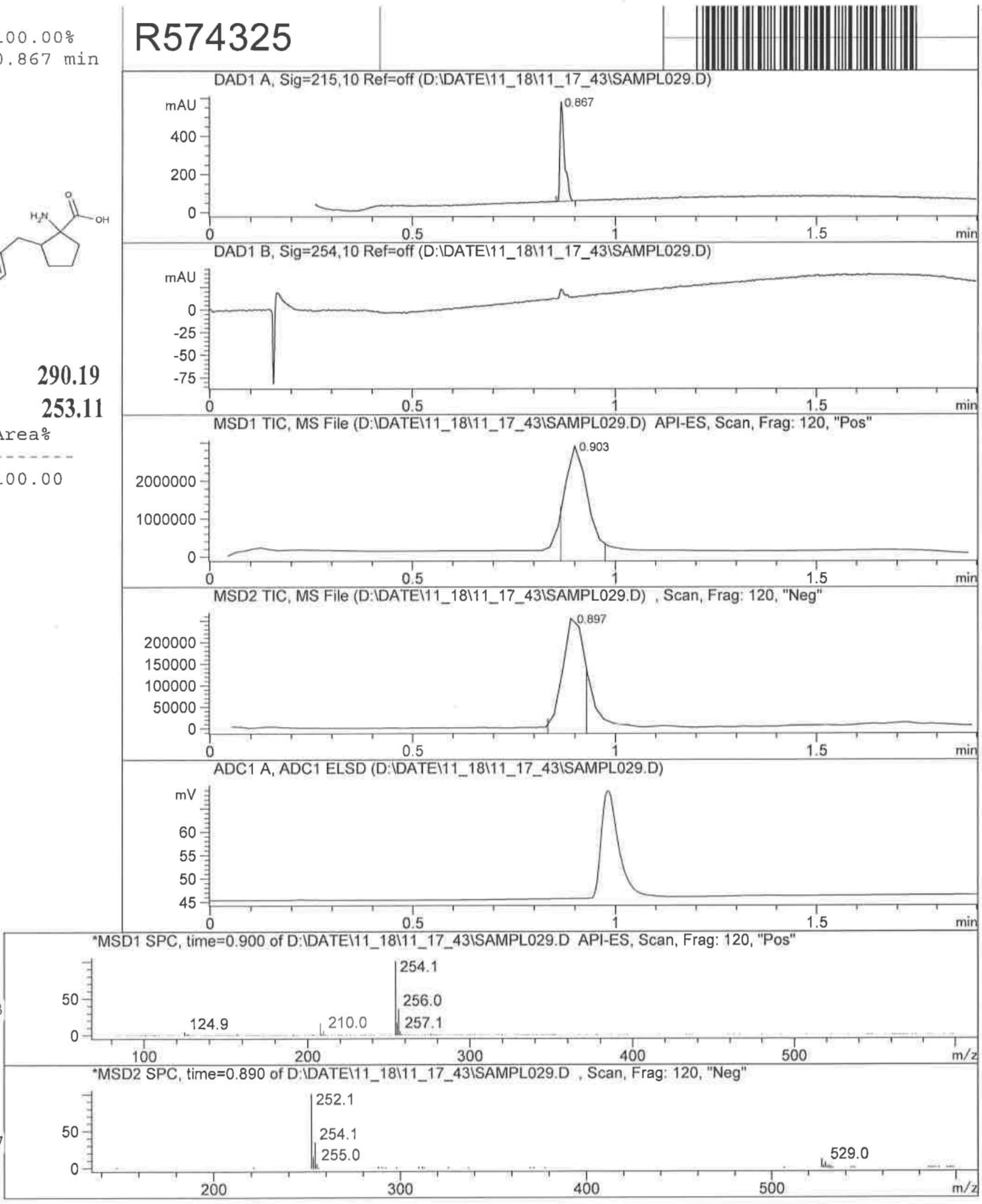

Inj.Date 11/18/2015 L P2-D-01 SL Acq. Method C:\HPCHEM\-> ->

Figure S56. LC-MS spectrum of compound **32**.

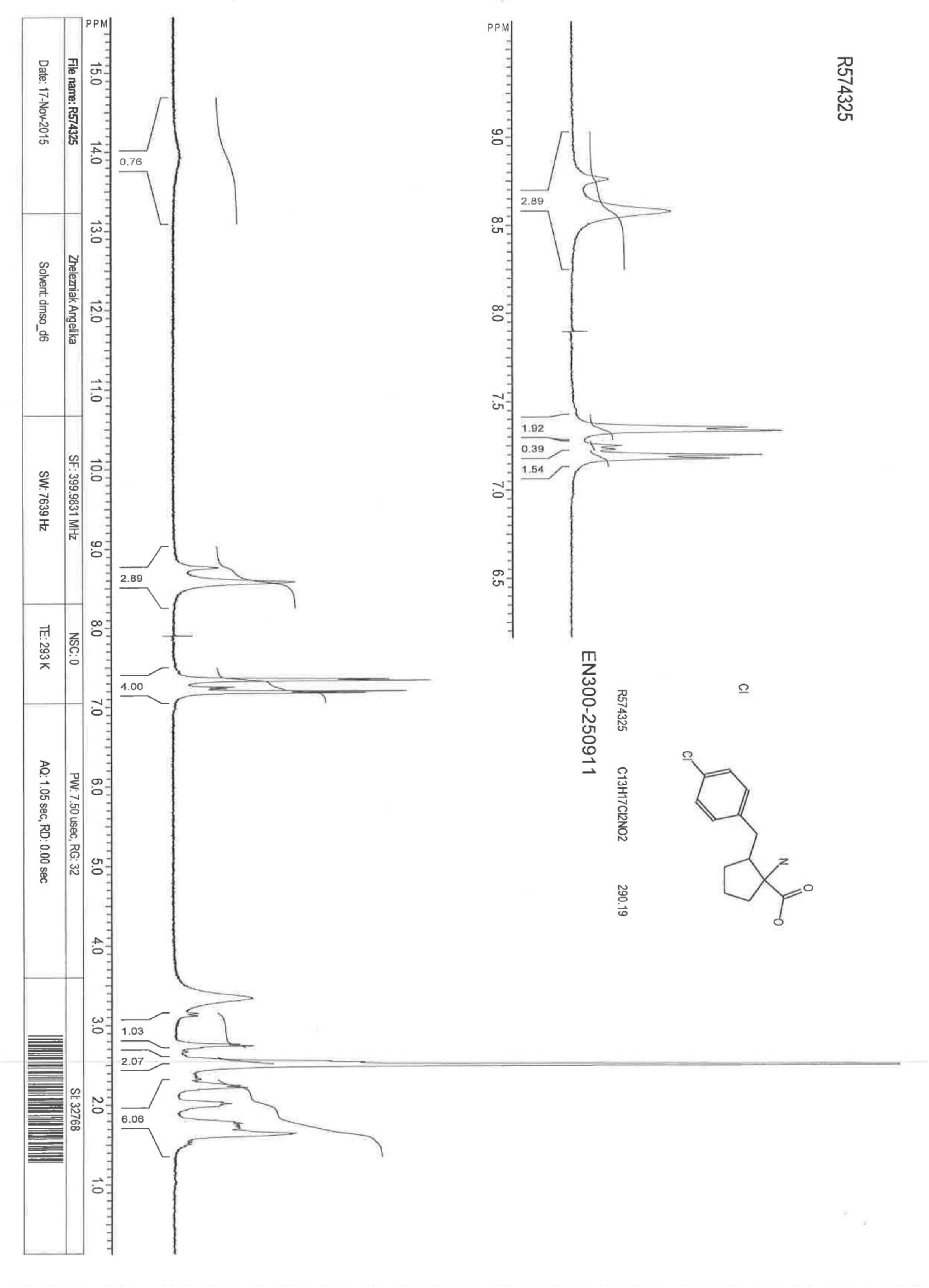

Figure S57. ^{1}H-NMR spectrum of compound **32**.

MaxPeak: 98.32%
Ret_Time: 0.823 min

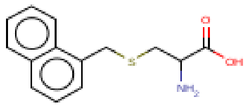

Mol Wt 261.339
Exact Mass 261.1

#	Time	Area%
1	0.823	98.32
2	1.032	1.68

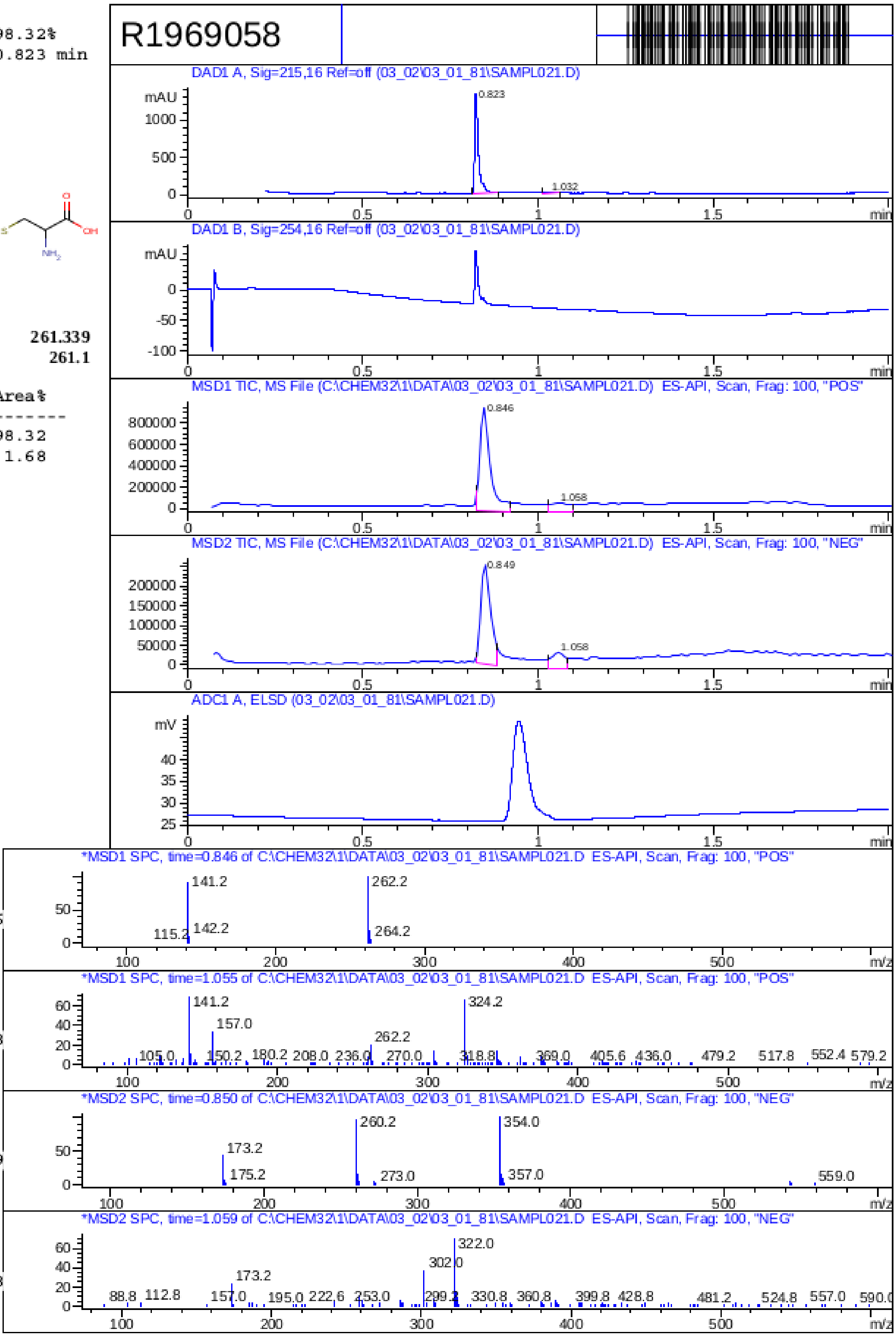

RT 0.846

RT 1.058

RT 0.849

RT 1.058

Inj.Date 3/1/2012 N P2-C-03 '4' Acq. Method C:\CHEM32\-> ->

Figure S58. LC-MS spectrum of compound **33**.

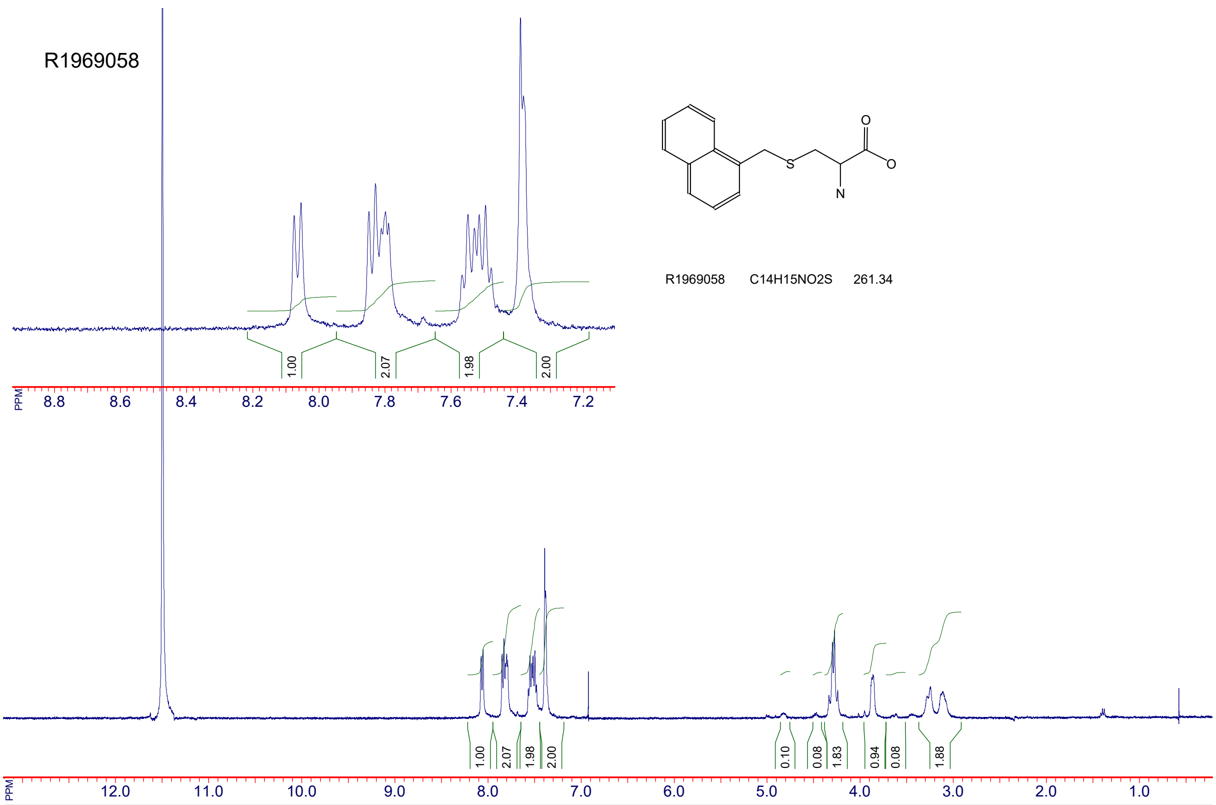

File name: R1969058	Operator: Reutova	SF: 399.9578 MHz	NSC: 0	PW: 7.50 usec, RG: 32	SI: 32768
Date: 02-Mar-2012	Solvent: cf3cood	SW: 5597 Hz	TE: 293 K	AQ: 1.61 sec, RD: 0.00 sec	*R1969058*

Figure S59. ^{1}H-NMR spectrum of compound **33**.

MaxPeak: 99.02%
Ret_Time: 0.676 min

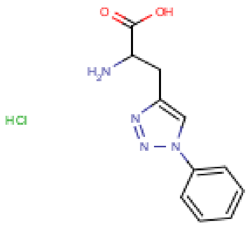

Mol Wt 268.7
Exact Mass 232.1

#	Time	Area%
1	0.639	0.98
2	0.676	99.02

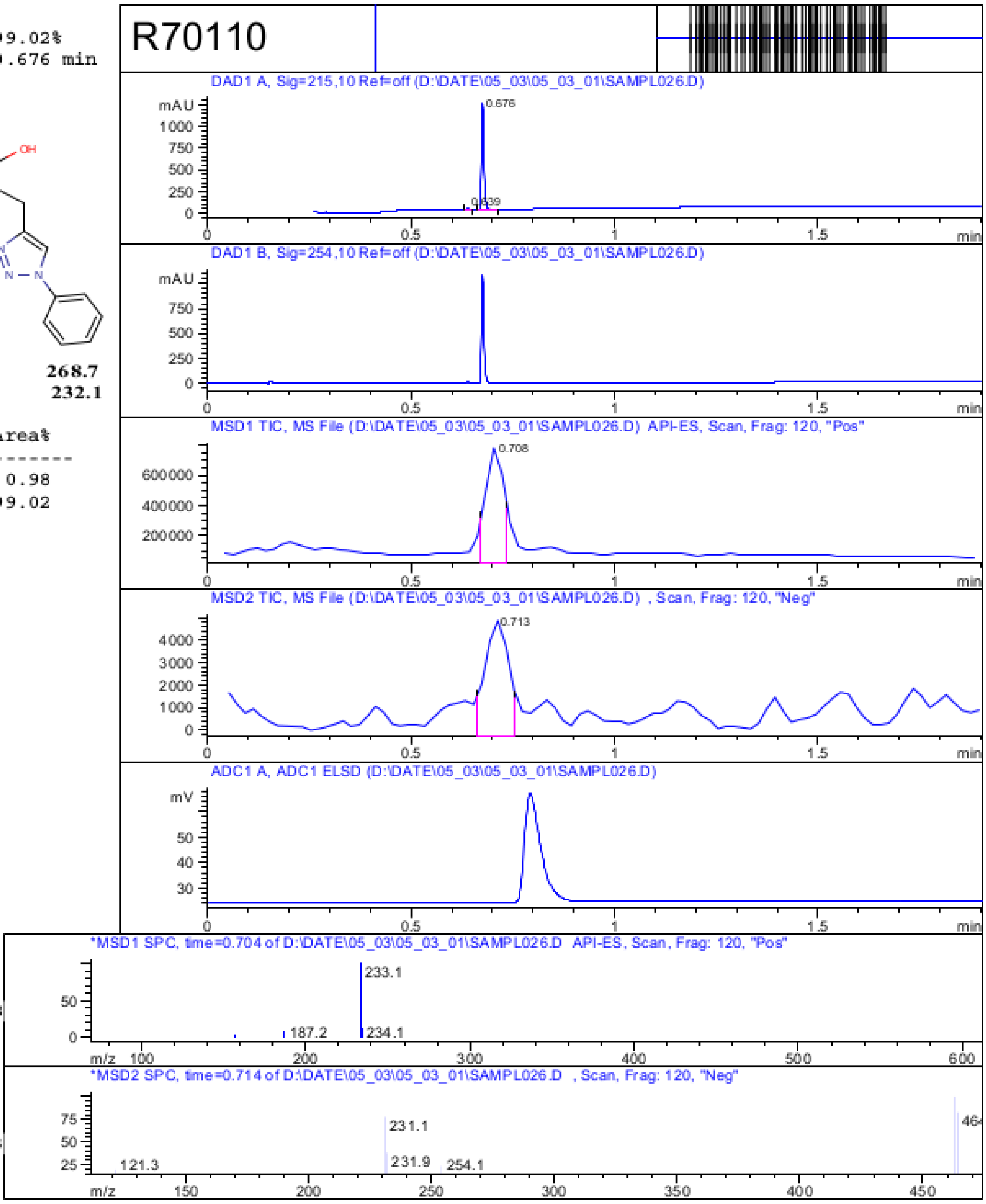

RT 0.708

RT 0.713

Inj.Date 5/4/2013 L P2-C-02 VL Acq. Method C:\HPCHEM\-> ->

Figure S60. LC-MS spectrum of compound **34**.

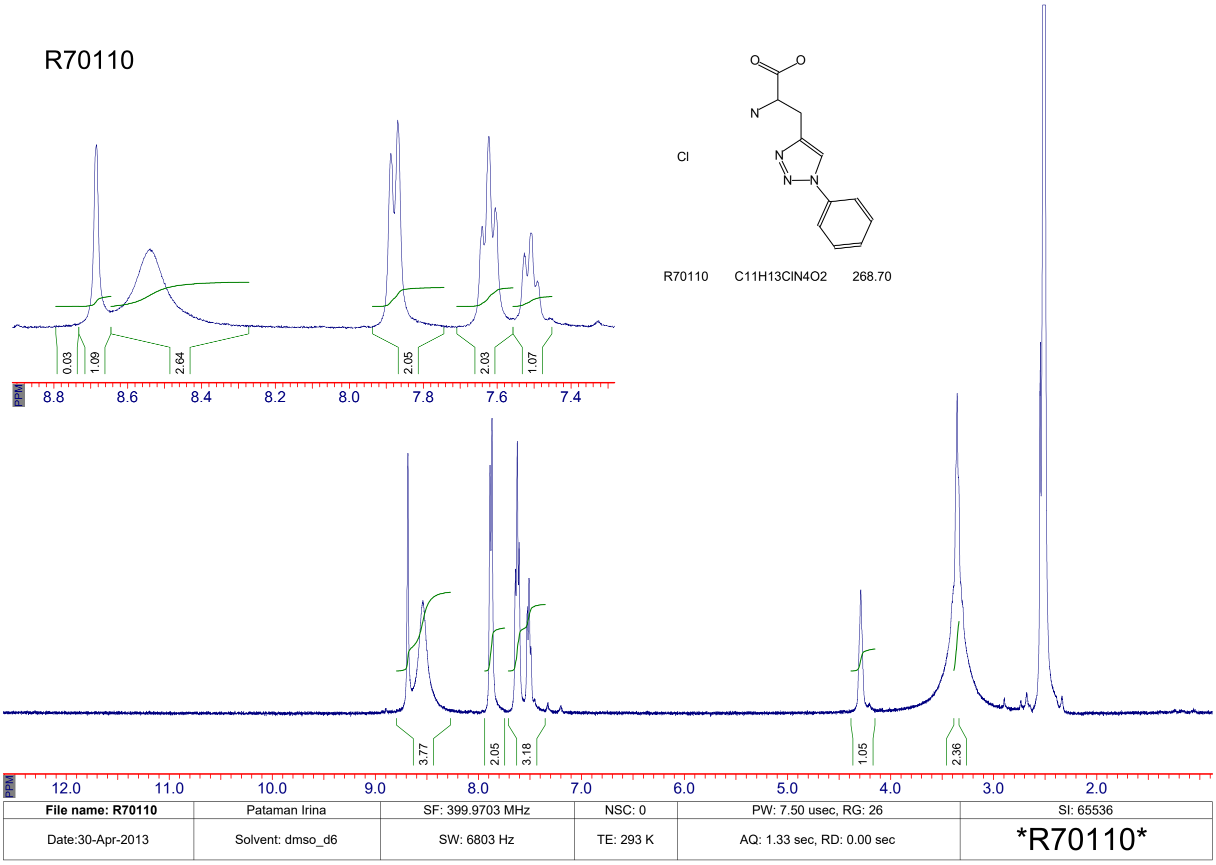

Figure S61. ^{1}H-NMR spectrum of compound **34**.

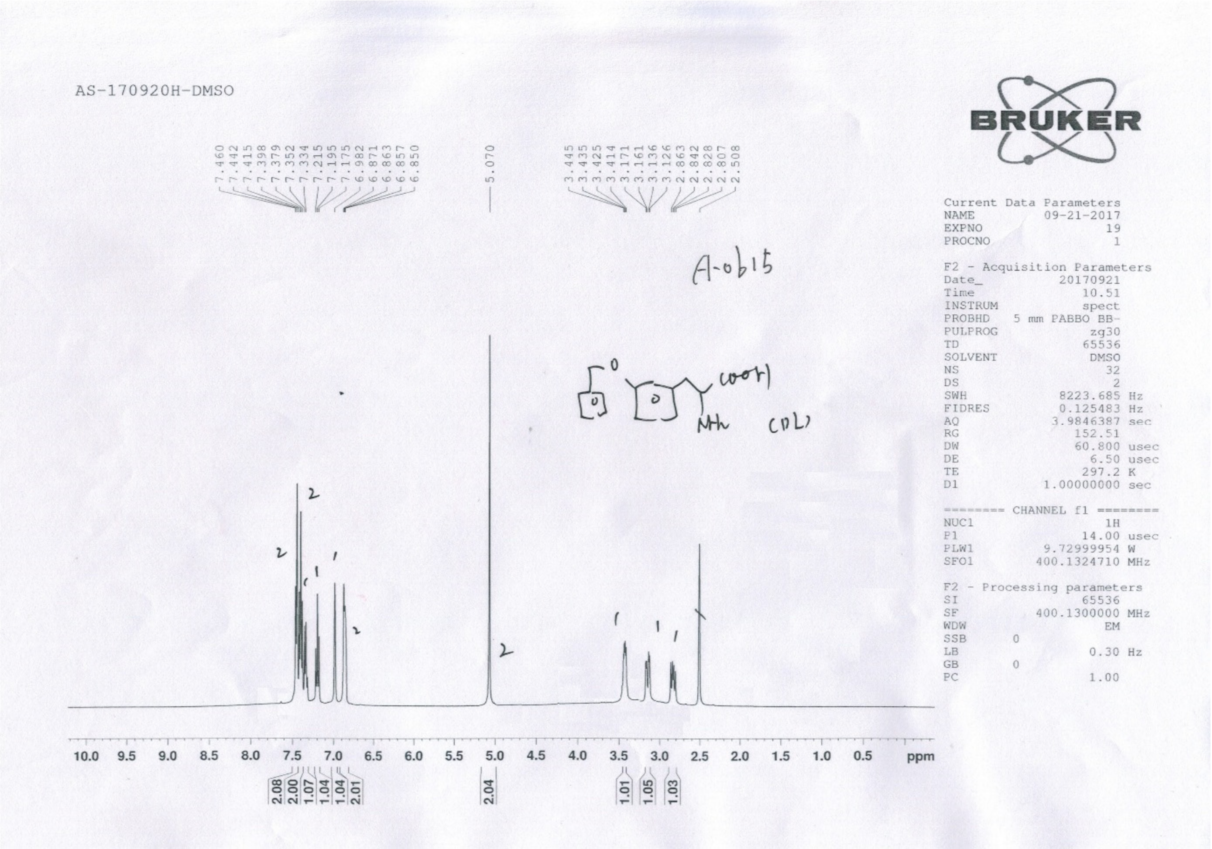

Figure S62. ^{1}H-NMR spectrum of compound **35**.

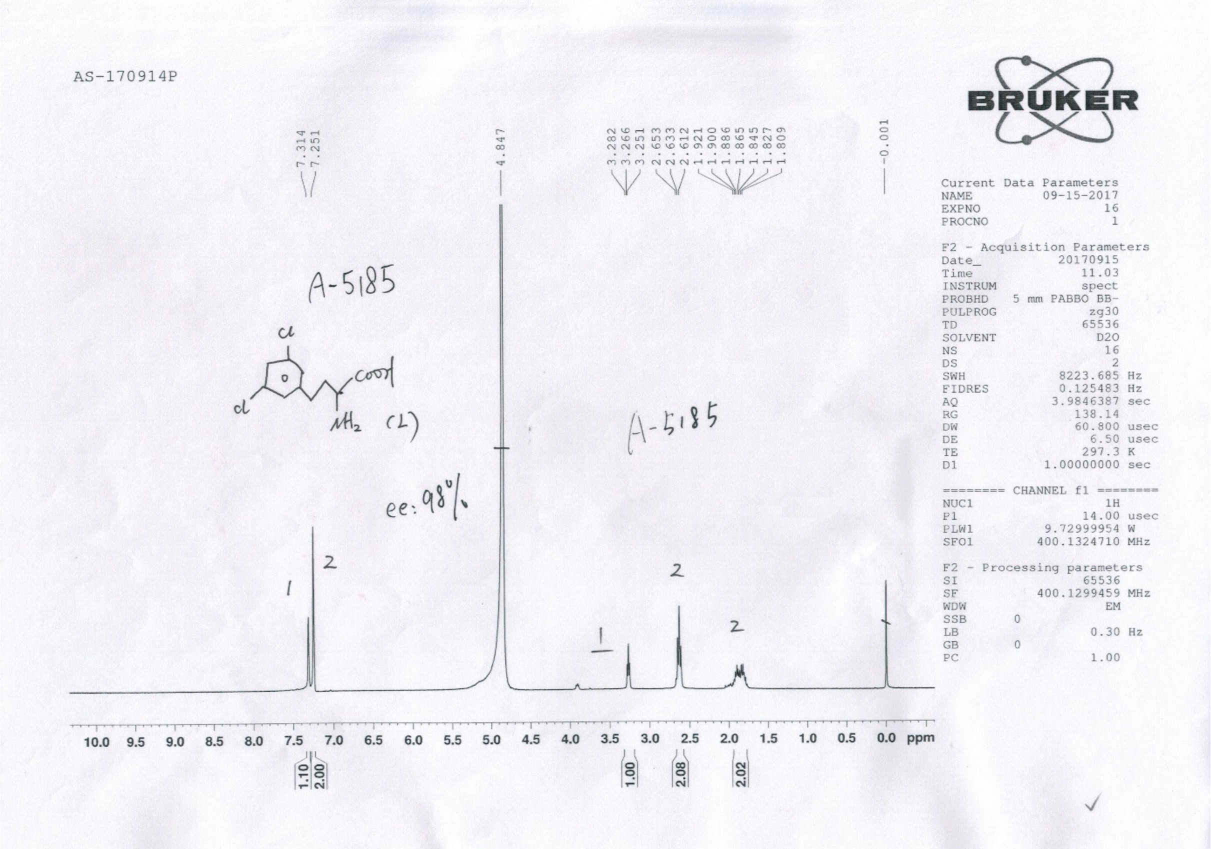

Figure S63. ^{1}H-NMR spectrum of compound **36**.

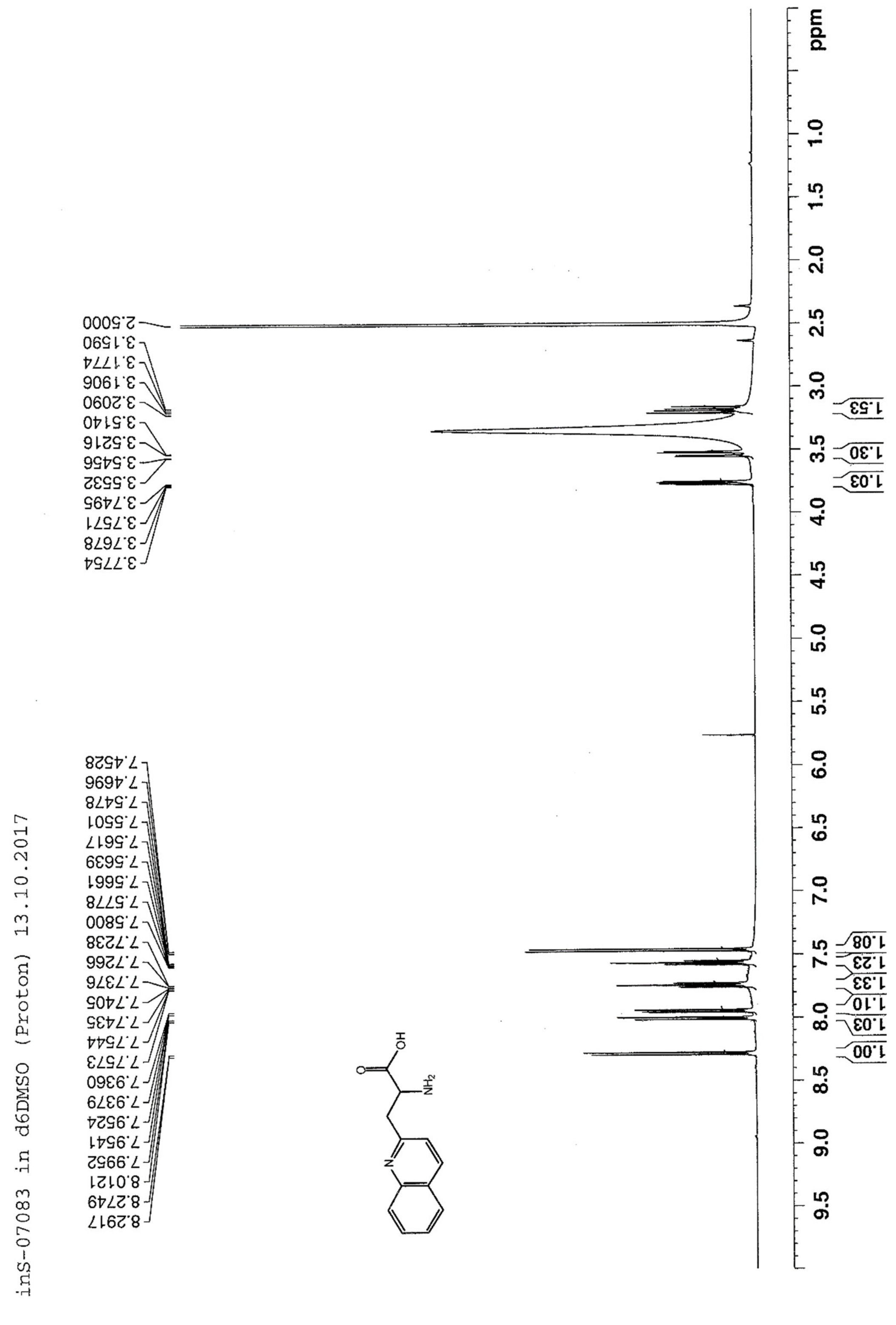

Figure S64. ^{1}H-NMR spectrum of compound **37**.

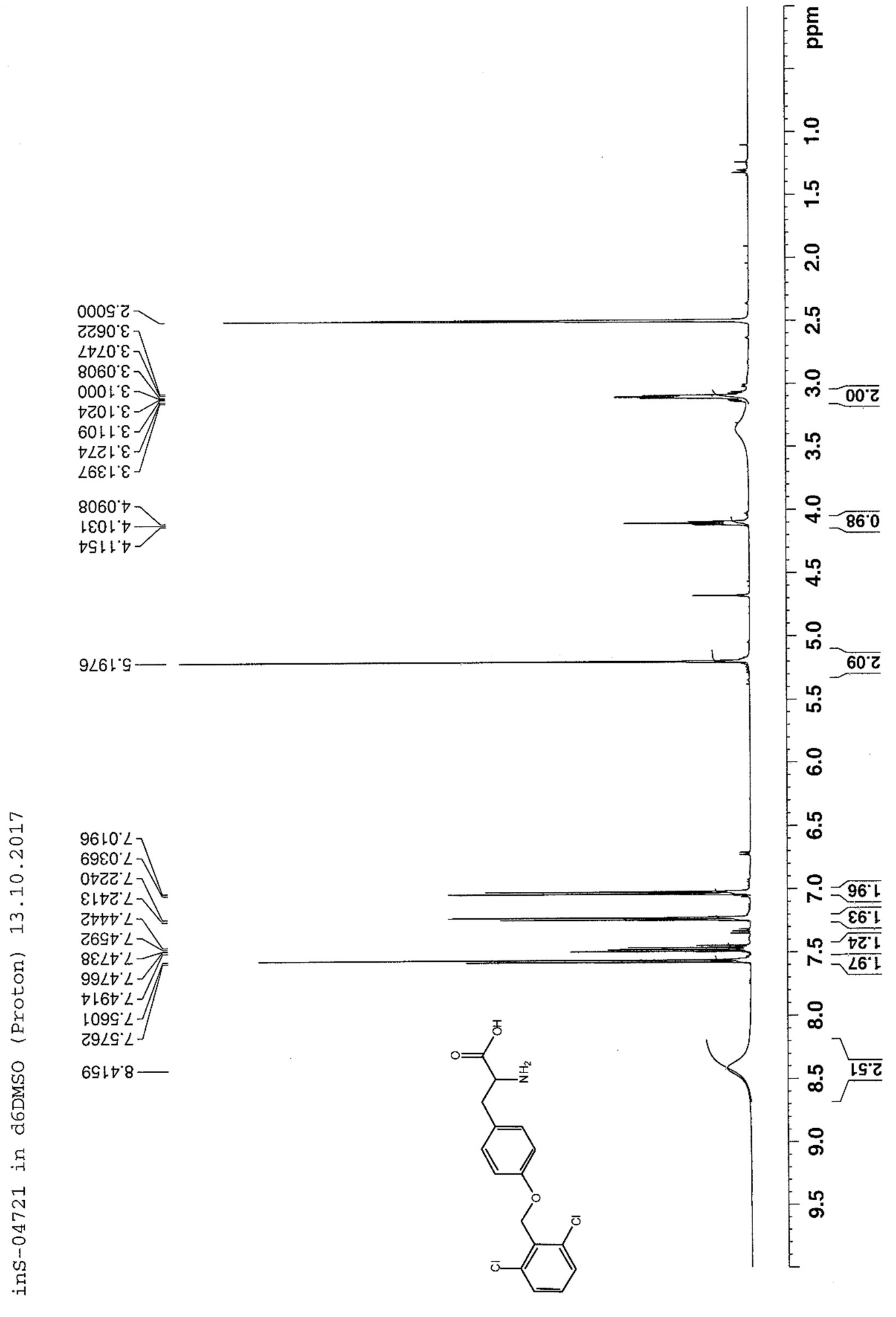

Figure S65. ^{1}H-NMR spectrum of compound **38**.

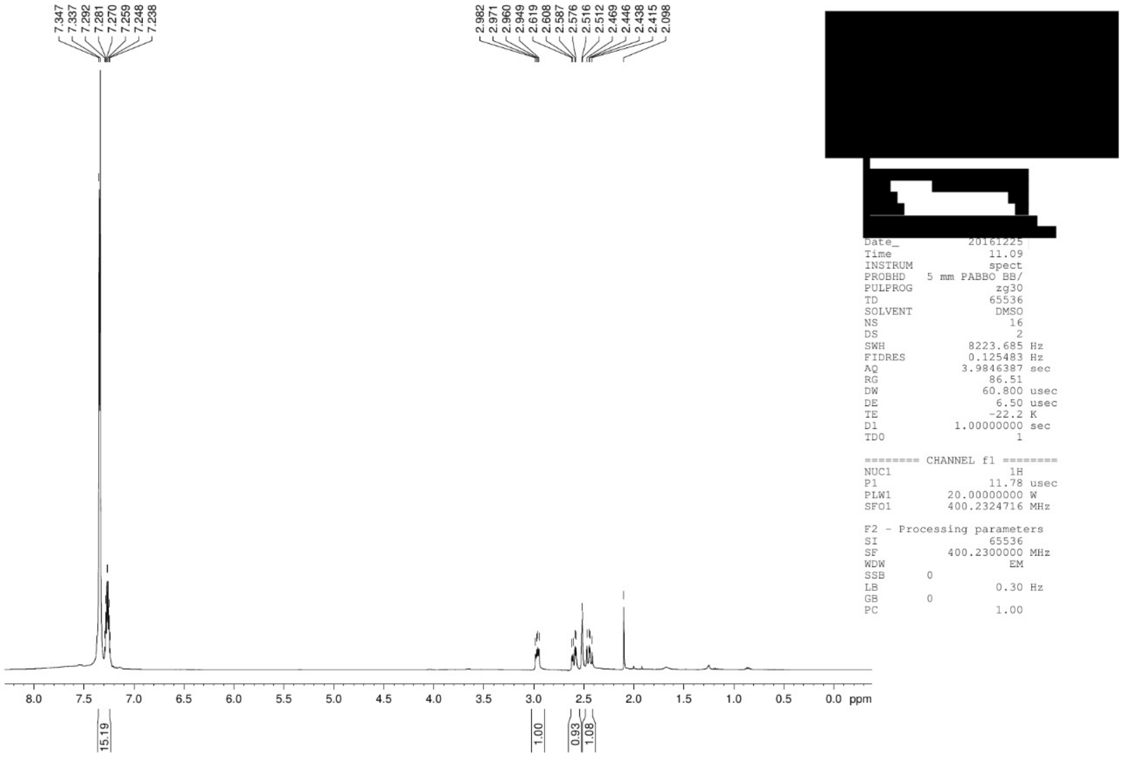

Figure S66. ^{1}H-NMR spectrum of compound **39**.

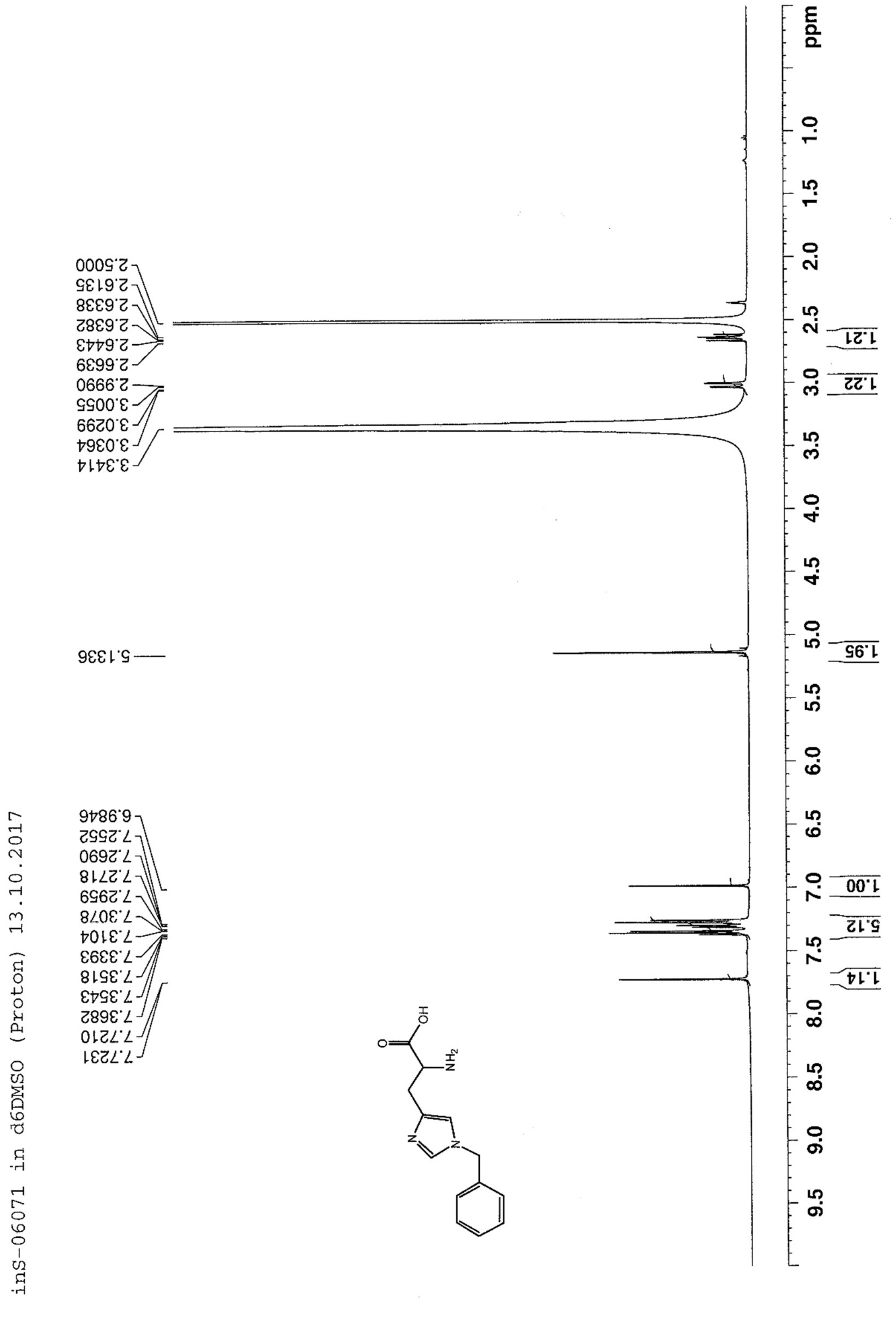

Figure S67. ^{1}H-NMR spectrum of compound **40**.

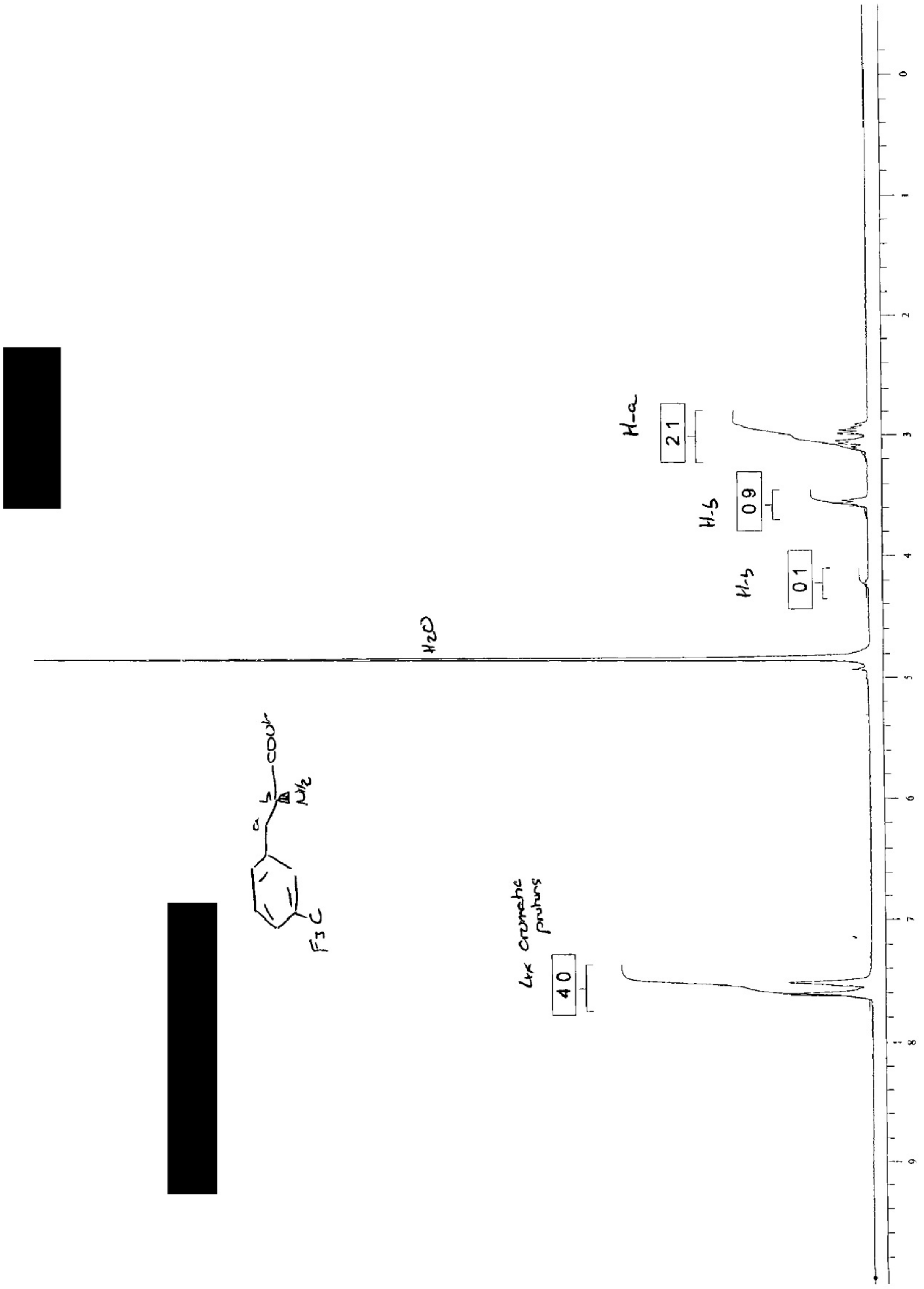

Figure S68. ^{1}H-NMR spectrum of compound **41**.

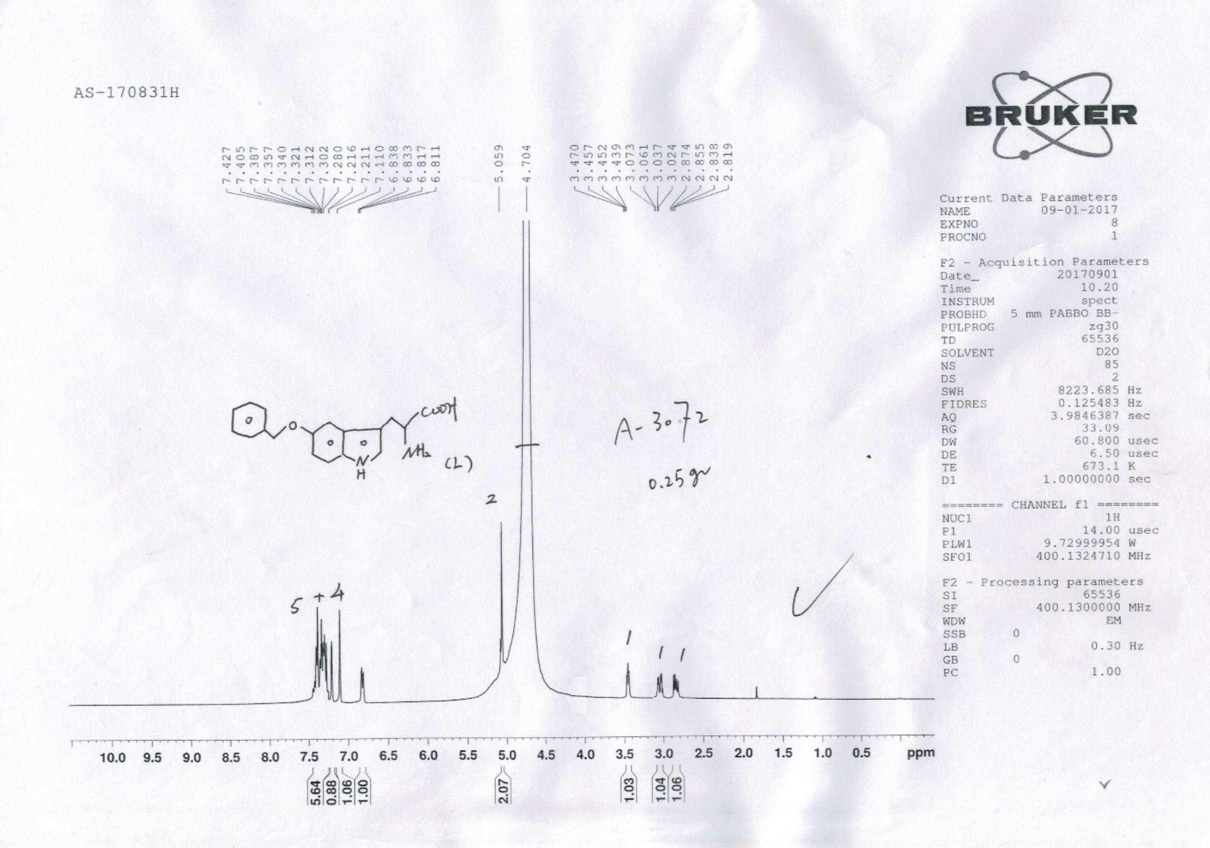

Figure S69. ^{1}H-NMR spectrum of compound **42**.
